# Supplementary material for: Bringing the uncultivated microbial majority of freshwater ecosystems into culture
Source: Nat Commun. 2025 Aug 26;16:7971. doi: 10.1038/s41467-025-63266-9 (PMC12381374; doi:10.1038/s41467-025-63266-9)
Supplement: Supplementary file 1 — Supplementary Information [file 41467_2025_63266_MOESM1_ESM.pdf]

Supplementary Information for

Bringing the uncultivated microbial majority of freshwater ecosystems into culture

Michaela M. Salcher[1]*, Paul Layoun[1,2], Clafy Fernandes[1,2], Maria-Cecilia Chiriac[1], Paul-Adrian Bulzu[1], Rohit Ghai[1], Tanja Shabarova[1], Vojtech Lanta[3], Cristiana Callieri[4], Bettina Sonntag[5], Thomas Posch[6], Fabio Lepori[7], Petr Znachor[1], Markus Haber[1]

Corresponding author: michaelasalcher@gmail.com

This PDF file includes:

Supplementary Notes

Supplementary Figures 1 to 21

Supplementary References

Supplementary Notes

16S rRNA classification of metagenomes

We sequenced metagenomes of the free-living microbial fraction (5-0.22 µm) of the same water samples as used for cultivation (n = 67) to determine the community composition by taxonomic assignment using two different methods: Taxonomic classification based on 59 single copy marker genes with SingleM[1] and 16S rRNA gene sequences, the most commonly used marker genes for microbial taxonomists. Results from SingleM classification and 16S rRNA gene sequence classification were significantly correlated ($R^2 = 0.9725$, $p < 0.0001$, linear regression, Supplementary Fig. 8, Supplementary Data 4, 6, 8). A detailed taxonomic overview of SingleM classification is given in the main text, while the taxonomic overview of 16S rRNA classification is as follows: A total of 2,735 genus/lineage-like taxa were identified in the 67 metagenomes, however, only 127 taxa accounted for >1% in any of the of metagenomic 16S rRNA datasets and made up the bulk of all prokaryotes (average of 87% of all reads). The remaining taxa were rare and occurred only sporadically (Supplementary Figs. 9, 10, Supplementary Data 9). This analysis is largely consistent with results from SingleM, where the most abundant 157 taxa made up on average 84% of all reads. The most abundant taxa in metagenomes were affiliated with the phyla *Actinomycetota* (acI or hgc_1 in SILVA, represented by *Nanopelagicus*, *Planktophila*, and other *Nanopelagicales*[2]; and uncultivated acIV classified as uncultivated *Microtrichales*, *Microtrichaceae*, *Iamiaceae*, and *Illumatobacteraceae* in SILVA), *Pseudomonadota* (*Fonsibacter* [reclassified to *Fontibacterium]*, *Methylopumilus*, *Polynucleobacter*, *Limnohabitans*, *Methylotenera*), *Bacteroidota* (*Fluviicola*, *Sediminibacterium*, *Flavobacterium*, NS11-12), *Cyanobacteriota* (*Cyanobium*), *Planctomycetota* (CL500-3), *Chloroflexota* (CL500-11), and *Verrucomicrobiota*. Our culture collection contained 19.2% of these abundant freshwater microbial lineages, most notably 10 lineages within the top 25 most abundant and prevalent taxa. If translated to relative abundances, we were successful in obtaining isolates that accounted for up to 71.7% of the microbial community in the epilimnion (48.4% on average) and up to 45.4% in the hypolimnion (24.9% on average).

Metagenomic fragment recruitment of cultures, MAGs and reference genomes

We used the 87 genomes from cultures, 1,294 medium-high quality MAGs (>50% completeness, <5% contamination) gained from the same water samples and dereplicated at the species level (95% identity) (Supplementary Data 12), and 324 genomes of their closest relatives obtained from public databases (Supplementary Data 13) for metagenomic fragment recruitment. Read mapping of our 67 lake metagenomes resulted in 129 highly abundant species spanning 65 genera (>5 × genome coverage per Gb of metagenome, Fig. 4, Supplementary Figs. 14, 15, Supplementary Data 16). Five species of the genus *Fontibacterium* (including the two new species isolated here) were by far the most abundant prokaryotes in the epilimnion (up to 117 × coverage), followed by *Verrucolacustris* gen. nov. (UBA3015) sp945874125 (*Verrucomicrobiota*; up to 52.6 ×), MAG-120802 sp947503395 (*Actinomycetota*; 50.8 ×), *Hahnella lacustris* gen. nov. (UBA2463, *Pseudomonadota*; 39.1 ×), three species of *Nanopelagicus* (25.5 to 38.1 ×), two Synechococcus_D (33 to 48.7 ×), and *Methylopumilus universalis* (31.8 ×). Notably, our culture collection contains all but MAG-120802 and *Nanopelagicus* sp. of these top abundant species. The hypolimnion was mainly dominated by the so far uncultivated species '*Ca*. Nitrosopumilus limneticus' (Archaea, max. 56 × coverage) and BD1 sp003670425 (*Chloroflexota*, max. 54 × coverage), followed by uncultivated *Nitrotoga*

(*Pseudomonadota*; max. 33.9 ×), *Nitrosarchaeum* (*Archaea*; 24.9 ×), and *Verrucomicrobiota* (VGWG01 and UBA953; max 21.1 and 22.6 ×, respectively) and taxa that appear to be common in both water strata (*Synechococcus*_D *lacustris*, different *Fontibacterium*, *Nanopelagicus* and *Planktophila*). Other abundant taxa from our culture collection (*Methylotenera, Lacustribacter* gen. nov.*, Mycobacterium, Sphingorhabdus, Hydrogenophaga, Rhodoluna, Limnohabitans, Fimbriicoccus* gen. nov.) were present at max. 2-17 × coverage. We further mapped 250 publicly available metagenomes from six continents and two seasonally resolved time-series (Lake Mendota, USA, 2008-2012; *n* = 94[3], Římov Reservoir, Czechia, 2015-2019; *n* = 81[4-6]) to the 47 dereplicated species from our genome collection. The Římov Reservoir was also one of the habitats used for isolating strains in 2019. Most species could be detected in multiple metagenomes; however, we also observed biogeographic differences, e.g., a conspicuous absence of several species in lakes in Africa, Australia, and South America (Supplementary Figs. 15, 16, Supplementary Data 17). Nevertheless, the presence of species from our culture collection in multiple samples confirms a global presence and high relevance in freshwater habitats.

Description of novel taxa according to SeqCode

Family *Acidimicrobilacustridaceae*

Etymology

[A.ci.di.mi.cro.bi.la.cus.tri.da'ce.ae] **N.L. masc. n.** *Acidimicrobilacustris*, referring to the type genus Acidimicrobilacustris; *-aceae*, ending to denote a family; **N.L. fem. pl. n.** *Acidimicrobilacustridaceae*, the Acidimicrobilacustris family

Nomenclatural type

Genus *Acidimicrobilacustris*

Description

A family of *Acidimicrobiales* also known as "acIV-D Actinobacteria" or "uncultured Microtrichales" from 16S rRNA gene analyses and as f__UBA8139 in GTDB, that is commonly found in freshwater environments. Consisting of the genus *Acidimicrobilacustris* and two species, *Acidimicrobilacustris thunensis* (GCA_965194345.1) and *Acidimicrobilacustris europaeus* (GCA_965194515.1), both isolated from freshwater lakes. Type species is *Acidimicrobilacustris thunensis* TE-7 (GCA_965194345.1). The closest cultivated relative is *Rhabdothermincola salaria* (*Iamiaceae*, GCF_021246445.1), with an average amino acid identity of 66.55% and an average nucleotide identity of 71.1%. As this related taxon is also included in family UBA8139 in GTDB and not in *Iamiaceae*, we propose the new family *Acidimicrobilacustridaceae*. Current GTDB (R220) classification: d__Bacteria; p__Actinomycetota; c__Acidimicrobiia; o__Acidimicrobiales; f__UBA8139.

Classification

Bacteria » *Actinomycetota* » *Acidimicrobiia* » *Acidimicrobiales* » *Acidimicrobilacustridaceae*

Registry URL

https://seqco.de/i:48714

Genus *Acidimicrobilacustris*

Etymology

[A.ci.di.mi.cro.bi.la.cus'tris] **N.L. neut. n.** *Acidimicrobium*, the genus Acidimicrobium, in reference to the order Acidimicrobiales; **L. masc. adj.** *lacustris*, of a lake, pond, pool; **N.L. masc. n.** *Acidimicrobilacustris*, a freshwater genus of Acidimicrobiales

Nomenclatural type

Species *Acidimicrobilacustris europaeus*^{Ts}

Description

Type genus of *Acidimicrobilacustridaceae* also known as “acIV-D Actinobacteria” or “uncultured Microtrichales” from 16S rRNA gene analyses and as g__F1-20-MAGs160 in GTDB, that is commonly found in freshwater environments. Consisting of two species, *Acidimicrobilacustris thunensis* (GCA_965194345.1) and *Acidimicrobilacustris europaeus* (GCA_965194515.1), both isolated from freshwater lakes. Type species is *Acidimicrobilacustris thunensis* TE-7 (GCA_965194345.1). The closest cultivated relative is *Rhabdothermincola salaria* (GCF_021246445.1), with an average amino acid identity of 66.55% and an average nucleotide identity of 71.1%. Current GTDB (R220) classification: d__Bacteria; p__Actinomycetota; c__Acidimicrobiia; o__Acidimicrobiales; f__UBA8139; g__F1-20-MAGs160.

Classification

Bacteria » *Actinomycetota* » *Acidimicrobiia* » *Acidimicrobiales* » *Acidimicrobilacustridaceae* » *Acidimicrobilacustris*

Registry URL

https://seqco.de/i:48715

Species *Acidimicrobilacustris europaeus*^{Ts}

Etymology

[e.u.ro.pae'us] **N.L. masc. adj.** *europaeus*, of/from Europe; named after the main occurrence in European lakes

Nomenclatural type

NCBI Assembly: GCA_965194515.1 ^{Ts}

Reference Strain

Strain sc|0039530: KE-4

Description

Type strain is *Acidimicrobilacustris europaeus* KE-4 (GCA_965194515.1), isolated from 5 m depth from Klíčava Reservoir, Czechia (date: 2019-04-25), *via* high-throughput dilution to extinction cultivation. KE-4 has a genome size of 2.35 Mbp with a genomic GC content of 58.2%, contains 3 rRNA genes and 44 tRNAs. The genome is complete, consisting of a circular chromosome. The genome contains genes encoding rhodopsins but lacks the biosynthetic pathway for retinal biosynthesis. No genes for flagella or pilus assembly and chemotaxis were annotated. Pathways for taurine degradation, assimilatory sulphate reduction and methane/alkanesulfonate oxidation and biosynthesis for all amino acids except histidine and threonine were predicted. Further, pathways for riboflavin, pyridoxal, NAD, coenzyme A, biotin and heme were identified. The closest cultivated relatives are *Rhabdothermincola salaria* (GCF_021246445.1) with an average amino acid identity of 64.98% and an average nucleotide identity of 69.8% and another newly proposed species, *Acidimicrobilacustris thunensis* TE-7 (GCA_965194345.1), with an AAI of 73.1% and an ANI of 72%. Current GTDB classification (R220): d__Bacteria; p__Actinomycetota; c__Acidimicrobiia; o__Acidimicrobiales; f__UBA8139; g__F1-20-MAGs160; s__F1-20-MAGs160 sp903827085.

Classification

Bacteria » *Actinomycetota* » *Acidimicrobiia* » *Acidimicrobiales* » *Acidimicrobilacustridaceae* » *Acidimicrobilacustris* » *Acidimicrobilacustris europaeus*^{Ts}

Registry URL

https://seqco.de/i:48713

Species *Acidimicrobilacustris europaeus*^{Ts}

Etymology

[e.u.ro.pae'us] **N.L. masc. adj.** *europaeus*, of/from Europe; named after the main occurrence in European lakes

Nomenclatural type

NCBI Assembly: GCA_965194515.1 [Ts]

Reference Strain

Strain sc|0039530: KE-4

Description

Type strain is *Acidimicrobilacustris europaeus* KE-4 (GCA_965194515.1), isolated from 5 m depth from Klíčava Reservoir, Czechia (date: 2019-04-25), *via* high-throughput dilution to extinction cultivation. KE-4 has a genome size of 2.35 Mbp with a genomic GC content of 58.2%, contains 3 rRNA genes and 44 tRNAs. The genome is complete, consisting of a circular chromosome. The genome contains genes encoding rhodopsins but lacks the biosynthetic pathway for retinal biosynthesis. No genes for flagella or pilus assembly and chemotaxis were annotated. Pathways for taurine degradation, assimilatory sulphate reduction and methane/alkanesulfonate oxidation and biosynthesis for all amino acids except histidine and threonine were predicted. Further, pathways for riboflavin, pyridoxal, NAD, coenzyme A, biotin and heme were identified. The closest cultivated relatives are *Rhabdothermincola salaria* (GCF_021246445.1) with an average amino acid identity of 64.98% and an average nucleotide identity of 69.8% and another newly proposed species, *Acidimicrobilacustris thunensis* TE-7 (GCA_965194345.1), with an AAI of 73.1% and an ANI of 72%. Current GTDB classification (R220): d__Bacteria; p__Actinomycetota; c__Acidimicrobiia; o__Acidimicrobiales; f__UBA8139; g__F1-20-MAGs160; s__F1-20-MAGs160 sp903827085.

Classification

Bacteria » *Actinomycetota* » *Acidimicrobiia* » *Acidimicrobiales* » *Acidimicrobilacustridaceae* » *Acidimicrobilacustris* » *Acidimicrobilacustris europaeus*[Ts]

Registry URL

https://seqco.de/i:48713

Species *Rhodoluna miladensis*

Etymology

[mi.la.den'sis] **N.L. fem. adj.** *miladensis*, pertaining to Lake Milada (Czechia), the isolation source of the type strain.

Nomenclatural type

NCBI Assembly: GCA_965234705.1 [Ts]

Reference Strain

Strain sc|0038944: MiE-23b

Description

Type strain is *Rhodoluna miladensis* MiE-23b (GCA_965234705.1), isolated from 5 m depth from Lake Milada, Czechia (date: 2019-10-15), *via* high-throughput dilution to extinction cultivation. MiE-23b has a genome size of 1.28 Mbp with a genomic GC content of 50.2%, contains 3 rRNA genes and 40 tRNAs. The genome is complete, consisting of a circular chromosome. The genome contains genes encoding rhodopsins but lacks the biosynthetic pathway for retinal biosynthesis. No genes for flagella or pilus assembly and chemotaxis were annotated. Pathways for the biosynthesis of all amino acids except for methionine and histidine were predicted. Further, pathways for riboflavin, pyridoxal, coenzyme A, and menaquinone were identified. The closest cultivated relative is *Rhodoluna lacicola* MWH-Ta8 (GCF_000699505), with an average amino acid identity of 65.06% and average nucleotide identity of 67.72%. Current GTDB classification (R220): d__Bacteria; p__Actinomycetota; c__Actinomycetia; o__Actinomycetales; f__Microbacteriaceae; g__Rhodoluna; s__.

Classification

Bacteria » *Actinomycetota* » *Actinomycetes* » *Micrococcales* » *Microbacteriaceae* » *Rhodoluna* » *Rhodoluna miladensis*

Registry URL

https://seqco.de/i:48729

Species *Mycobacterium aquicola*

Etymology

[a.qui'co.la] **L. fem. n.** *aqua*, water; **L. masc. suff.** *-cola*, inhabitant; **N.L. masc. n.** *aquicola*, inhabitant of water, referring to the isolation source from a freshwater lake.

Nomenclatural type

NCBI Assembly: GCA_965234515.1 Ts

Reference Strain

Strain sc|0038945: MiE-22

Description

Type strain is *Mycobacterium aquicola* MiE-22 (GCA_965234515.1), isolated from 5 m depth from Lake Milada, Czechia (date: 2019-10-15), *via* high-throughput dilution to extinction cultivation. MiE-22 has a genome size of 3.4 Mbp with a genomic GC content of 66.3%, contains 3 rRNA genes and 57 tRNAs. The genome is a high-quality draft consisting of 3 contigs. The genome contains genes encoding rhodopsins. No genes for flagella or pilus assembly and chemotaxis were annotated. Pathways for taurine degradation, assimilatory sulphate reduction and the biosynthesis of all amino acids except for histidine and aspartate were predicted. Further, pathways for many vitamins and cofactors (riboflavin, pyridoxal, NAD, coenzyme A, biotin, tetrahydrofolate, heme, cobalamin and menaquinone) were identified. The closest cultivated relative is *Mycobacterium massilipolynesiensis* M26 (GCF_001494595.1), with an average amino acid identity of 71.31% and average nucleotide identity of 76.47%. Current GTDB classification (R220): d__Bacteria; p__Actinomycetota; c__Actinomycetia; o__Mycobacteriales; f__Mycobacteriaceae; g__Mycobacterium; s__Mycobacterium sp945897705.

Classification

Bacteria » *Actinomycetota* » *Actinomycetes* » *Mycobacteriales* » *Mycobacteriaceae* » *Mycobacterium* » *Mycobacterium aquicola*

Registry URL

https://seqco.de/i:48708

Species *Planktophila warneckei*

Etymology

[war.ne.cke'i] **N.L. masc. gen. n.** *warneckei*, of Warnecke, named after the deceased German scientist Falk Warnecke, who intensively worked on the ecology of freshwater Planktophila

Nomenclatural type

NCBI Assembly: GCA_965234675.1 Ts

Reference Strain

Strain sc|0038946: RE-8

Description

Type strain is *Planktophila warneckei* RE-8 (GCA_965234675.1), isolated from 0.5 m depth from the Římov reservoir, Czechia (date: 2019-04-23), *via* high-throughput dilution to extinction cultivation. RE-8 has a genome size of 1.4 Mbp with a genomic GC content of 46.2%, contains 3 rRNA genes and 38 tRNAs. The genome is complete, consisting of a circular chromosome. The genome contains genes encoding rhodopsins. No genes for flagella or pilus assembly and chemotaxis were annotated. Pathways for the biosynthesis of all amino acids except for histidine were predicted. Further, pathways for riboflavin, pyridoxal, coenzyme A, and menaquinone biosynthesis were identified. The closest cultivated relative is Actinobacteria bacterium IMCC26103 (GCA_002284875.1), with an average amino acid identity of 72.2% and average

nucleotide identity of 70.96%. Current GTDB classification (R220): d__Bacteria; p__Actinomycetota; c__Actinomycetia; o__Nanopelagicales; f__Nanopelagicaceae; g__Planktophila; s__.

Classification

Bacteria » *Actinomycetota* » *Actinomycetes* » *Nanopelagicales* » *Nanopelagicaceae* » *Planktophila* » *Planktophila warneckei*

Registry URL

https://seqco.de/i:48741

Species *Planktophila turicensis*

Etymology

[tu.ri.cen'sis] **N.L. neut. n.** *Turicum*, the Latin name of Zurich; **N.L. fem. adj.** *turicensis*, referring to Turicum, the Latin name of Zurich, pertaining to Lake Zurich, the isolation source of the type strain

Nomenclatural type

NCBI Assembly: GCA_965234405.1 ^{Ts}

Reference Strain

Strain sc|0038793: ZE-9

Description

Type species is *Planktophila turicensis* ZE-9 (GCA_965234405.1), isolated from 5 m depth from Lake Zurich, Switzerland (date: 2019-11-13), *via* high-throughput dilution to extinction cultivation. ZE-9 has a genome size of 1.4 Mbp with a genomic GC content of 48.5%, contains 3 rRNA genes and 39 tRNAs. The genome is complete, consisting of a circular chromosome. The genome contains genes encoding rhodopsins. No genes for flagella or pilus assembly and chemotaxis were annotated. Pathways for taurine degradation and the biosynthesis of all amino acids except for histidine were predicted. Further, pathways for riboflavin, pyridoxal, coenzyme A, NAD, and menaquinone biosynthesis were identified. The closest cultivated relative is '*Candidatus* Planktophila sulfonica' MMS-IA-56 (GCF_002288185.1), with an average nucleotide identity of 94.22%. Current GTDB classification (R220): d__Bacteria; p__Actinomycetota; c__Actinomycetia; o__Nanopelagicales; f__Nanopelagicaceae; g__Planktophila; s__.

Classification

Bacteria » *Actinomycetota* » *Actinomycetes* » *Nanopelagicales* » *Nanopelagicaceae* » *Planktophila* » *Planktophila turicensis*

Registry URL

https://seqco.de/i:49118

Species *Planktophila grossartii*

Etymology

[gros.sar.ti'i] **N.L. masc. gen. n.** *grossartii*, named after the German scientist Hans-Peter Grossart, who greatly contributed to the ecological characterization of freshwater Actinomycetota.

Nomenclatural type

NCBI Assembly: GCA_965234365.1 ^{Ts}

Reference Strain

Strain sc|0038786: RE-3

Description

Type species is *Planktophila grossartii* RE-3 (GCA_965234365.1), isolated from 0.5 m depth from the Římov reservoir, Czechia (date: 2019-04-23), *via* high-throughput dilution to extinction cultivation. RE-3 has a genome size of 1.4 Mbp with a genomic GC content of 48.8 %, contains 3 rRNA genes and 38 tRNAs. The genome is complete, consisting of a circular chromosome. The

genome contains genes encoding rhodopsins. No genes for flagella or pilus assembly and chemotaxis were annotated. Pathways for taurine degradation and the biosynthesis of all amino acids except for histidine were predicted. Further, pathways for riboflavin, pyridoxal, coenzyme A, NAD, and menaquinone biosynthesis were identified. The closest cultivated relative is Actinobacteria bacterium IMCC25003 (GCA_002284855.1) with an average nucleotide identity of 90.33%. Current GTDB classification (R220): d__Bacteria; p__Actinomycetota; c__Actinomycetia; o__Nanopelagicales; f__Nanopelagicaceae; g__Planktophila; s__Planktophila sp009701085.

Classification

Bacteria » *Actinomycetota* » *Actinomycetes* » *Nanopelagicales* » *Nanopelagicaceae* » *Planktophila* » *Planktophila grossartii*

Registry URL

https://seqco.de/i:49103

Genus *Fimbriicoccus*

Etymology

[Fim.bri.i.coc'cus] **L. fem. n.** *fimbria*, fibres, threads, fringe, and in biology, fimbriae; **N.L. masc. n.** *coccus*, coccus; **N.L. masc. n.** *Fimbriicoccus*, a coccus with fimbriae

Nomenclatural type

Species *Fimbriicoccus planktonicus*[Ts]

Description

Genus of *Fimbriimonadaceae* that is commonly found in freshwater environments. Type species is *Fimbriicoccus planktonicus* MsE-15 (GCA_965234685.1). The closest cultivated relative is *Fimbriimonas ginsengisoli* Gsoil 348 (GCF_000724625.1), with an average amino acid identity of 55.26% and an average nucleotide identity of 66.05%. Current GTDB classification (R220): d__Bacteria; p__Armatimonadota; c__Fimbriimonadia; o__Fimbriimonadales; f__Fimbriimonadaceae; g__Fimbriimonas.

Classification

Bacteria » *Armatimonadota* » *Fimbriimonadia* » *Fimbriimonadales* » *Fimbriimonadaceae* » *Fimbriicoccus*

Registry URL

https://seqco.de/i:49105

Species *Fimbriicoccus planktonicus*[Ts]

Etymology

[plan.kto'ni.cus] **N.L. masc. adj.** *planktonicus*, living in the plankton, planktonic; from Gr. masc. adj. planktos, wandering

Nomenclatural type

NCBI Assembly: GCA_965234685.1 [Ts]

Reference Strain

Strain sc|0038787: MsE-15

Description

Type species is *Fimbriicoccus planktonicus* MsE-15 (GCA_965234685.1), isolated from 5 m depth from Lake Most, Czechia (date: 2019-07-30), *via* high-throughput dilution to extinction cultivation. MsE-15 has a genome size of 3.29 Mbp with a genomic GC content of 56.5 %, contains 3 rRNA genes and 48 tRNAs. The genome is complete, consisting of a circular chromosome. No genes for flagella assembly and chemotaxis were annotated. Pathways for the biosynthesis of all amino acids except for alanine, proline, and histidine were predicted. Further, pathways for riboflavin, pyridoxal, NAD, coenzyme A, THF, and menaquinone biosynthesis were identified. The closest cultivated relative is *Fimbriimonas ginsengisoli* Gsoil 348 (GCF_000724625.1) with an average amino acid identity of 55.26% and an average nucleotide

identity of 66.05%. Current GTDB classification (R220): d__Bacteria; p__Armatimonadota; c__Fimbriimonadia; o__Fimbriimonadales; f__Fimbriimonadaceae; g__Fimbriimonas; s__Fimbriimonas sp945882415.

Classification

Bacteria » *Armatimonadota* » *Fimbriimonadia* » *Fimbriimonadales* » *Fimbriimonadaceae* » *Fimbriicoccus* » *Fimbriicoccus planktonicus*[Ts]

Registry URL

https://seqco.de/i:49104

Species *Leadbetterella lacustris*

Etymology

[la.cus'tris] **N.L. fem. adj.** *lacustris*, of a lake; referring to the habitat from where the strain was isolated.

Nomenclatural type

NCBI Assembly: GCA_965234335.1 [Ts]

Reference Strain

Strain sc|0038947: RE-19

Description

Type strain is *Leadbetterella lacustris* RE-19 (GCA_965234335.1), isolated from 0.5 m depth from the Římov reservoir, Czechia (date: 2019-08-05), *via* high-throughput dilution to extinction cultivation. RE-19 has a genome size of 5 Mbp with a genomic GC content of 37.48 %, contains 9 rRNA genes and 36 tRNAs. The genome is a high-quality draft consisting of 36 contigs. The genome contains genes encoding rhodopsins. Genes for gliding motility (*gldBDHJKLMN*, *sprA*) were annotated in the genome. Pathways for assimilatory sulphate reduction, methane/alkanesulfonate oxidation and the biosynthesis of all amino acids were predicted. Further, pathways for riboflavin, NAD, coenzyme A, THF, menaquinone, and heme biosynthesis were identified. The closest cultivated relative is *Lacihabitans soyangensis* KCTC23259 (GCF_024343775.1), with an average amino acid identity of 85.5% and average nucleotide identity of 82.97%. *Lacihabitans* is grouped with the genus *Leadbetterella* in GTDB. Current GTDB classification (R220): d__Bacteria; p__Bacteroidota; c__Bacteroidia; o__Cytophagales; f__Spirosomaceae; g__Leadbetterella; s__Leadbetterella flu*via*le_A.

Classification

Bacteria » *Bacteroidota* » *Cytophagia* » *Cytophagales* » *Spirosomataceae* » *Leadbetterella* » *Leadbetterella lacustris*

Registry URL

https://seqco.de/i:48724

Species *Flavobacterium neuenschwanderi*

Etymology

[ne.u.en.schwan.der'i] **L. masc. gen. n.** *neuenschwanderi*, of Neuenschwander, named after the Swiss scientist Stefan Neuenschwander, who studied freshwater Flavobacteria in Lake Zurich.

Nomenclatural type

NCBI Assembly: GCA_965234425.1 [Ts]

Reference Strain

Strain sc|0038948: GE-10

Description

Type strain is *Flavobacterium neuenschwanderi* GE-10 (GCA_965234425.1), isolated from 5 m depth from Greifensee, Switzerland (date: 2019-04-03), *via* high-throughput dilution to extinction cultivation. GE-10 has a genome size of 3.56 Mbp with a genomic GC content of 33.1%, contains 4 rRNA genes and 38 tRNAs. The genome is a high-quality draft consisting of 44 contigs. The genome contains genes encoding rhodopsins. Genes for gliding motility (*gldBDHJKLMN*, *sprA*)

were annotated in the genome. Pathways for assimilatory sulfate reduction and the biosynthesis of all amino acids were predicted. Further, pathways for riboflavin, NAD, coenzyme A, biotin, THF, menaquinone, and heme were identified. The closest cultivated relative is *Flavobacterium psychrotolerans* RB1R5 (GCF_003097635.1), with an average amino acid identity of 74.81% and average nucleotide identity of 74.37%. Current GTDB classification (R220): d__Bacteria; p__Bacteroidota; c__Bacteroidia; o__Flavobacteriales; f__Flavobacteriaceae; g__Flavobacterium; s__.

Classification

Bacteria » Bacteroidota » Flavobacteriia » Flavobacteriales » Flavobacteriaceae » Flavobacterium » Flavobacterium neuenschwanderi

Registry URL

https://seqco.de/i:48730

Species *Flavobacterium rarum*

Etymology

[ra'rum] **L. neut. adj.** *rarum*, sporadic or uncommon, referring to the sporadic isolation and recovery from disparate sources of members of this species

Nomenclatural type

NCBI Assembly: GCA_965234445.1 Ts

Reference Strain

Strain sc|0038949: TH-M1

Description

Type strain is *Flavobacterium rarum* TH-M1 (GCA_965234445.1), isolated from 180 m from Lake Thun, Switzerland (date: 2019-04-08), *via* high-throughput dilution to extinction cultivation. TH-M1 has a genome size of 4.26 Mbp with a genomic GC content of 33.4%, contains 6 rRNA genes and 51 tRNAs. The genome is a high-quality draft consisting of 29 contigs. The genome contains genes encoding rhodopsins. Genes for gliding motility (*gldBDHJKLMN*, *sprA*) were annotated in the genome. Pathways for taurine degradation, assimilatory sulfate reduction and the biosynthesis of all amino acids were predicted. Further, pathways for thiamine, riboflavin, NAD, pantothenate, coenzyme A, biotin, THF, menaquinone, and heme were identified. The closest cultivated relative is *Flavobacterium praedii* IMCC34515 (GCF_026810365.1), with an average amino acid identity of 85.51% and average nucleotide identity of 83.7%. Current GTDB classification (R220): d__Bacteria; p__Bacteroidota; c__Bacteroidia; o__Flavobacteriales; f__Flavobacteriaceae; g__Flavobacterium; s__.

Classification

Bacteria » Bacteroidota » Flavobacteriia » Flavobacteriales » Flavobacteriaceae » Flavobacterium » Flavobacterium rarum

Registry URL

https://seqco.de/i:48736

Species *Caulobacter lacus*

Etymology

[la'cus] **L. gen. n.** *lacus*, of a lake, referring to the habitat from which the type strain was isolated.

Nomenclatural type

NCBI Assembly: GCA_965234345.1 Ts

Reference Strain

Strain sc|0038950: MiH-16

Description

Type strain is *Caulobacter lacus* MiH-16 (GCA_965234345.1), isolated from 15 m depth from Lake Milada, Czechia (date: 2019-07-23), *via* high-throughput dilution to extinction cultivation. MiH-16 has a genome size of 3.6 Mbp with a genomic GC content of 67.6%, contains 3 rRNA genes and 47 tRNAs. The genome is a high-quality draft consisting of 5 contigs. The genome contains genes encoding rhodopsins. Genes for flagellar assembly and chemotaxis were annotated in the genome. Pathways for taurine degradation, assimilatory sulfate reduction and methane/alkanesulfonate oxidation and the biosynthesis of all amino acids were predicted. Further, pathways for thiamine, riboflavin, NAD, coenzyme A, pimeloyl-ACP, THF, and heme biosynthesis were identified. The closest cultivated relative is *Caulobacter* sp. Root1455 (GCF_001426905.1), with an average amino acid identity of 64% and average nucleotide identity of 74.7%. Current GTDB classification (R220): d__Bacteria; p__Pseudomonadota; c__Alphaproteobacteria; o__Caulobacterales; f__Caulobacteraceae; g__Caulobacter; s__Caulobacter sp903858185.

Classification

Bacteria » *Pseudomonadota* » *Alphaproteobacteria* » *Caulobacterales* » *Caulobacteraceae* » *Caulobacter* » *Caulobacter lacus*

Registry URL

https://seqco.de/i:48721

Species *Aquidulcibacter rimovensis*

Etymology

[ri.mo.ven'sis] **N.L. masc. adj.** *rimovensis*, pertaining to the Římov Reservoir (Czechia), the isolation source of the type strain.

Nomenclatural type

NCBI Assembly: GCA_965234525.1 [Ts]

Reference Strain

Strain sc|0038951: RH-10

Description

Type strain is *Aquidulcibacter rimovensis* RH-10 (GCA_965234525.1), isolated from 30 m depth from Římov Reservoir, Czechia (date: 2019-08-05), *via* high-throughput dilution to extinction cultivation. RH-10 has a genome size of 3.3 Mbp with a genomic GC content of 55.4%, contains 3 rRNA genes and 42 tRNAs. The genome is a high-quality draft consisting of 2 contigs. The genome contains genes encoding anoxygenic aerobic phototrophy (*pufABLM*). Genes for flagellar assembly and chemotaxis were annotated in the genome. Pathways for taurine degradation, assimilatory sulfate reduction and methane/alkanesulfonate oxidation and the biosynthesis of all amino acids were predicted. Further, pathways for riboflavin, NAD, coenzyme A, pimeloyl-ACP, THF, and heme biosynthesis were identified. The closest cultivated relatives are *Aquidulcibacter paucihalophilus* TH1-2 (GCF_002105465.1) with an average amino acid identity of 87.3% and average nucleotide identity of 80.2% and another newly proposed species, *Aquidulcibacter miladensis* MiH-15 (GCA_965234355.1), with an AAI of 93.2% and an ANI of 89.4%. Current GTDB classification (R220): d__Bacteria; p__Pseudomonadota; c__Alphaproteobacteria; o__Caulobacterales; f__TH1-2; g__Aquidulcibacter; s__Aquidulcibacter sp027532555.

Classification

Bacteria » *Pseudomonadota* » *Alphaproteobacteria* » *Caulobacterales* » *Caulobacteraceae* » *Aquidulcibacter* » *Aquidulcibacter rimovensis*

Registry URL

https://seqco.de/i:48737

Species *Aquidulcibacter miladensis*

Etymology

[mi.la.den'sis] **N.L. masc. adj.** *miladensis*, pertaining to Lake Milada (Czechia), the isolation source of the type strain.

Nomenclatural type

NCBI Assembly: GCA_965234355.1 [Ts]

Reference Strain

Strain sc|0038952: MiH-15

Description

Type strain is *Aquidulcibacter miladensis* MiH-15 (GCA_965234355.1), isolated from 15 m depth from Lake Milada, Czechia (date: 2019-07-23), *via* high-throughput dilution to extinction cultivation. MiH-15 has a genome size of 3.2 Mbp with a genomic GC content of 55.7%, contains 3 rRNA genes and 41 tRNAs. The genome is a high-quality draft consisting of 8 contigs. The genome contains genes encoding anoxygenic aerobic phototrophy (*pufABLM*). Genes for flagellar assembly and chemotaxis were annotated in the genome. Pathways for taurine degradation, assimilatory sulfate reduction and methane/alkanesulfonate oxidation and the biosynthesis of all amino acids were predicted. Further, pathways for riboflavin, NAD, coenzyme A, pimeloyl-ACP, THF, and heme biosynthesis were identified. The closest cultivated relatives are *Aquidulcibacter paucihalophilus* TH1-2 (GCF_002105465.1) with an average amino acid identity of 87.1% and average nucleotide identity of 80.5% and another newly proposed species, *Aquidulcibacter rimovensis* RH-10 (GCA_965234525.1), with an AAI of 93.2% and an ANI of 89.4%. Current GTDB classification (R220): d__Bacteria; p__Pseudomonadota; c__Alphaproteobacteria; o__Caulobacterales; f__TH1-2; g__Aquidulcibacter; s__Aquidulcibacter sp945891505.

Classification

Bacteria » *Pseudomonadota* » *Alphaproteobacteria* » *Caulobacterales* » *Caulobacteraceae* » *Aquidulcibacter* » *Aquidulcibacter miladensis*

Registry URL

https://seqco.de/i:48727

Species *Fontibacterium abundans*

Etymology

[a.bun'dans] **L. neut. part. adj.** *abundans*, abundant, referring to high global abundances.

Nomenclatural type

NCBI Assembly: GCA_965235095.1 [Ts]

Reference Strain

Strain sc|0038953: MiE-29

Description

Type strain is *Fontibacterium abundans* MiE-29 (GCA_965235095.1), isolated from 5 m depth from Lake Milada, Czechia (date: 2019-10-15), *via* high-throughput dilution to extinction cultivation. MiE-29 has a genome size of 1.1 Mbp with a genomic GC content of 29.4%, contains 3 rRNA genes and 31 tRNAs. The genome is a high-quality draft consisting of 2 contigs. The genome contains genes encoding rhodopsins and the biosynthetic pathway for retinal biosynthesis. No genes for flagella or pilus assembly and chemotaxis were annotated. Pathways

for glycolate oxidation and the biosynthesis of 16 amino acids were predicted. Further, pathways for riboflavin, NAD, coenzyme A, and heme biosynthesis were identified. The closest cultivated relatives are *Fontibacterium commune*, syn. '*Candidatus* Fonsibacter ubiquis' LSUCC0530 (GCF_002688585.1; later reclassified to '*Ca.* Allofontibacter communis'), with an average amino acid identity of 86.5% and average nucleotide identity of 85.2% and another newly proposed species, *Fontibacterium medardense* ME-17 (GCA_965235075.1), with an AAI of 92.7% and an ANI of 91.1%. Current GTDB classification (R220): d__Bacteria; p__Pseudomonadota; c__Alphaproteobacteria; o__Pelagibacterales; f__Pelagibacteraceae; g__Fonsibacter; s__.

Classification

Bacteria » *Pseudomonadota* » *Alphaproteobacteria* » "Pelagibacterales" » "Pelagibacteraceae" » *Fontibacterium* » *Fontibacterium abundans*

Registry URL

https://seqco.de/i:48693

Species *Fontibacterium medardense*

Etymology

[me.dar.den'se] **L. neut. adj.** *medardense*, pertaining to Lake Medard (Czechia), the isolation source of the type strain.

Nomenclatural type

NCBI Assembly: GCA_965235075.1 [Ts]

Reference Strain

Strain sc|0038954: ME-17

Description

Type strain is *Fontibacterium medardense* ME-17 (GCA_965235075.1), isolated from 5 m depth from Lake Medard, Czechia (date: 2019-10-22), *via* high-throughput dilution to extinction cultivation. ME-17 has a genome size of 1.1 Mbp with a genomic GC content of 29.6%, contains 3 rRNA genes and 31 tRNAs. The genome is complete, consisting of a circular chromosome. The genome contains genes encoding rhodopsins and the biosynthetic pathway for retinal biosynthesis. No genes for flagella or pilus assembly and chemotaxis were annotated. Pathways for glycolate oxidation and the biosynthesis of 16 amino acids were predicted. Further, pathways for riboflavin, NAD, coenzyme A, and heme biosynthesis were identified. The closest cultivated relatives are '*Candidatus* Fonsibacter ubiquis' LSUCC0530 (GCF_002688585.1; later reclassified to '*Ca.* Allofontibacter communis'), with an average amino acid identity of 87.2% and average nucleotide identity of 85.2% and another newly proposed species, *Fontibacterium abundans* MiE-29 (GCA_965235095.1), with an AAI of 92.7% and an ANI of 91.1%. Current GTDB classification (R220): d__Bacteria; p__Pseudomonadota; c__Alphaproteobacteria; o__Pelagibacterales; f__Pelagibacteraceae; g__Fonsibacter; s__Fonsibacter sp018882565.

Classification

Bacteria » *Pseudomonadota* » *Alphaproteobacteria* » "Pelagibacterales" » "Pelagibacteraceae" » *Fontibacterium* » *Fontibacterium medardense*

Registry URL

https://seqco.de/i:48726

Species *Rhabdaerophilum aquaticum*

Etymology

[a.qua.ti'cum] **L. neut. adj.** *aquaticum*, living or found in the water

Nomenclatural type

NCBI Assembly: GCA_965234495.1 [Ts]

Reference Strain

Strain sc|0038955: MsE-M23

Description

Type strain is *Rhabdoaerophilum aquaticum* MsE-M23 (GCA_965234495.1), isolated from 5 m depth from Lake Most, Czechia (date: 2019-07-30), *via* high-throughput dilution to extinction cultivation. MsE-M23 has a genome size of 34 Mbp with a genomic GC content of 58.6%, contains 3 rRNA genes and 46 tRNAs. The genome is complete, consisting of a circular chromosome and one circular plasmid. Genes for flagellar assembly and chemotaxis were annotated in the genome. Pathways for cyanate and urea degradation, thiosulfate oxidation (Sox pathway), glycolate oxidation and the biosynthesis of all amino acids were predicted. Further, pathways for thiamine, riboflavin, NAD, coenzyme A, THF, and heme biosynthesis were identified. The closest cultivated relative is *Rhabdaerophilum calidifontis* SYSU G02060 (GCF_008641065.1), with an average amino acid identity of 66.3% and average nucleotide identity of 70.3%. Current GTDB classification (R220): d__Bacteria; p__Pseudomonadota; c__Alphaproteobacteria; o__Rhizobiales; f__Beijerinckiaceae; g__Rhabdaerophilum; s__.

Classification

Bacteria » *Pseudomonadota* » *Alphaproteobacteria* » *Hyphomicrobiales* » *Rhabdaerophilaceae* » *Rhabdaerophilum* » *Rhabdaerophilum aquaticum*

Registry URL

https://seqco.de/i:48703

Species *Tabrizicola rara*

Etymology

[ra'ra] **L. fem. adj.** *rara*, sporadic or uncommon, referring to the sporadic isolation and recovery from disparate sources of members of this species

Nomenclatural type

NCBI Assembly: GCA_965234505.1 ^{Ts}

Reference Strain

Strain sc|0038956: LH-M10

Description

Type strain is *Tabrizicola rara* LH-M10 (GCA_965234505.1), isolated from 50 m depth from Lake Lugano, Switzerland (date: 2019-11-05), *via* high-throughput dilution to extinction cultivation. LH-M10 has a genome size of 4.2 Mbp with a genomic GC content of 62.8%, contains 4 rRNA genes and 48 tRNAs. The genome is a high-quality draft consisting of 22 contigs. The genome contains the complete Calvin cycle for carbon fixation via RuBisCO. Genes for flagellar assembly were annotated in the genome. Pathways for urea and taurine degradation, glycolate oxidation and the biosynthesis of all amino acids were predicted. Further, pathways for riboflavin, coenzyme A, cobalamin, and heme biosynthesis were identified. The closest cultivated relative is *Rhodobacter thermarum* YIM 73036 (GCF_003574395.1; Basonym: *Tabrizicola thermarum* Khan et al. 2019), with an average amino acid identity of 75.7% and average nucleotide identity of 77.1%. Current GTDB classification (R220): d__Bacteria; p__Pseudomonadota; c__Alphaproteobacteria; o__Rhodobacterales; f__Rhodobacteraceae; g__Tabrizicola; s__Tabrizicola sp903917595.

Classification

Bacteria » *Pseudomonadota* » *Alphaproteobacteria* » *Rhodobacterales* » *Paracoccaceae* » *Tabrizicola* » *Tabrizicola rara*

Registry URL

https://seqco.de/i:48735

Genus *Allotabrizicola*

Etymology

[A.llo.ta.bri.zi'co.la] **Gr. masc. pron.** *allos*, another, other, different; **N.L. fem. n.** *Tabrizicola*, a bacterial genus; **N.L. fem. n.** *Allotabrizicola*, another Tabrizicola genus

Nomenclatural type

Species *Allotabrizicola aquatica*[Ts]

Description

Type species is *Allotabrizicola aquatica* RE-M30 (GCA_965194375.1). The closest cultivated relative is *Tabrizicola oligotrophica* KMS-5 (GCF_011008935.1) with an average amino acid identity of 79.2% and average nucleotide identity of 79.5%. As the closest relative is classified as Tabrizicola_A in GTDB, we propose a new genus that encompasses both species. The type species of the genus *Tabrizicola* (*Tabrizicola aquatica*, GCF_002900975.1), has average amino acid identities of 68.4-69.2% and average nucleotide identities of 73.7-74.8% to *Tabrizicola oligotrophica* KMS-5 (GCF_011008935.1) and *Allotabrizicola aquatica* RE-M30. Current GTDB classification (R220): d__Bacteria; p__Pseudomonadota; c__Alphaproteobacteria; o__Rhodobacterales; f__Rhodobacteraceae; g__Tabrizicola_A.

Classification

Bacteria » *Pseudomonadota* » *Alphaproteobacteria* » *Rhodobacterales* » *Paracoccaceae* » *Allotabrizicola*

Registry URL

https://seqco.de/i:48698

Species *Allotabrizicola aquatica*[Ts]

Etymology

[a.qua.ti'ca] **L. fem. adj.** *aquatica*, living or found in the water.

Nomenclatural type

NCBI Assembly: GCA_965194375.1 [Ts]

Reference Strain

Strain sc|0038957: RE-M30

Description

Type strain is *Allotabrizicola aquatica* RE-M30 (GCA_965194375.1), isolated from 0.5 m depth from the Římov Reservoir, Czechia (date: 2019-08-05), *via* high-throughput dilution to extinction cultivation. RE-M30 has a genome size of 3.6 Mbp with a genomic GC content of 61.9%, contains 6 rRNA genes and 49 tRNAs. The genome is a high-quality draft consisting of 20 contigs. The genome contains genes encoding anoxygenic aerobic phototrophy (*pufABLM*). Genes for flagellar assembly were annotated in the genome. Pathways for urea and taurine degradation, glycolate oxidation and the biosynthesis of all amino acids were predicted. Further, pathways for riboflavin, pantothenate, coenzyme A, and heme biosynthesis were identified. The closest cultivated relative is *Tabrizicola oligotrophica* KMS-5 (GCF_011008935.1), with an average amino acid identity of 79.2% and average nucleotide identity of 79.5%. Current GTDB classification (R220): d__Bacteria; p__Pseudomonadota; c__Alphaproteobacteria; o__Rhodobacterales; f__Rhodobacteraceae; g__Tabrizicola_A; s__.

Classification

Bacteria » *Pseudomonadota* » *Alphaproteobacteria* » *Rhodobacterales* » *Paracoccaceae* » *Allotabrizicola* » *Allotabrizicola aquatica*[Ts]

Registry URL

https://seqco.de/i:48697

Species *Sphingorhabdus rara*

Etymology

[ra'ra] **L. fem. adj.** *rara*, sporadic or uncommon, referring to the sporadic isolation and recovery from disparate sources of members of this species

Nomenclatural type

NCBI Assembly: GCA_965234565.1 [Ts]

Reference Strain

Strain sc|0038958: RE-M21a

Description

Type strain is *Sphingorhabdus rara* RE-M21a (GCA_965234565.1), isolated from 0.5 m depth from the Římov Reservoir, Czechia (date: 2019-08-05), *via* high-throughput dilution to extinction cultivation. RE-M21a has a genome size of 3.2 Mbp with a genomic GC content of 57.5%, contains 3 rRNA genes and 45 tRNAs. The genome is a high-quality draft consisting of 4 contigs. The genome contains genes encoding rhodopsins and the biosynthetic pathway for retinal biosynthesis. No genes for flagellar or pilus assembly and chemotaxis were annotated. Pathways for taurine degradation, assimilatory sulfate reduction and methane/alkanesulfonate oxidation and the biosynthesis of all amino acids were predicted. Further, pathways for thiamine, riboflavin, pantothenate, NAD, coenzyme A, THF, and heme biosynthesis were identified. The closest cultivated relative is *Sphingorhabdus pulchriflava* GY_G (GCF_003367235.1), with an average amino acid identity of 76.8% and average nucleotide identity of 75.4%. Current GTDB classification (R220): d__Bacteria; p__Pseudomonadota; c__Alphaproteobacteria; o__Sphingomonadales; f__Sphingomonadaceae; g__Sphingorhabdus_B; s__Sphingorhabdus_B sp021298455.

Classification

Bacteria » *Pseudomonadota* » *Alphaproteobacteria* » *Sphingomonadales* » *Sphingomonadaceae* » *Sphingorhabdus* » *Sphingorhabdus rara*

Registry URL

https://seqco.de/i:48734

Species *Sphingorhabdus communis*

Etymology

[com.mu'nis] **L. fem. adj.** *communis*, common, referring to a widespread distribution in freshwater lakes.

Nomenclatural type

NCBI Assembly: GCA_965234695.1 [Ts]

Reference Strain

Strain sc|0038959: GE-11

Description

Type strain is *Sphingorhabdus communis* GE-11 (GCA_965234695.1), isolated from 5 m depth from Lake Greifensee, Switzerland (date: 2019-04-03), *via* high-throughput dilution to extinction cultivation. GE-11 has a genome size of 2.4 Mbp with a genomic GC content of 54.9%, contains 3 rRNA genes and 42 tRNAs. The genome is complete, consisting of a circular chromosome. The genome contains genes encoding rhodopsins and the biosynthetic pathway for retinal biosynthesis. No genes for flagellar or pilus assembly and chemotaxis were annotated. Pathways for assimilatory sulfate reduction and methane/alkanesulfonate oxidation and the biosynthesis of all amino acids were predicted. Further, pathways for riboflavin, NAD, coenzyme A, THF, and heme biosynthesis were identified. The closest cultivated relative is *Sphingorhabdus wooponensis* 03SU3-P (GCF_003933235.1), with an average amino acid identity of 87.4% and average nucleotide identity of 80.5%. Current GTDB classification (R220): d__Bacteria; p__Pseudomonadota; c__Alphaproteobacteria; o__Sphingomonadales; f__Sphingomonadaceae; g__Sphingorhabdus_B; s__Sphingorhabdus_B sp903821685.

Classification

Bacteria » *Pseudomonadota* » *Alphaproteobacteria* » *Sphingomonadales* » *Sphingomonadaceae* » *Sphingorhabdus* » *Sphingorhabdus communis*

Registry URL

https://seqco.de/i:48711

Species *Polynucleobacter hoetzingerianus*

Etymology

[hoet.zin.ge.ri.a'nus] **N.L. masc. adj.** *hoetzingerianus*, named after the Austrian scientist Mattias Hoetzinger, who characterized several species of the genus Polynucleobacter

Nomenclatural type

NCBI Assembly: GCA_965234555.1 [Ts]

Reference Strain

Strain sc|0038960: RE-M21

Description

Type strain is *Polynucleobacter hoetzingerianus* RE-M21 (GCA_965234555.1), isolated from 0.5 m depth from the Římov Reservoir, Czechia (date: 2019-08-05), *via* high-throughput dilution to extinction cultivation. RE-M21 has a genome size of 1.8 Mbp with a genomic GC content of 46.6%, contains 3 rRNA genes and 40 tRNAs. The genome is complete, consisting of a circular chromosome. The genome contains genes encoding anoxygenic aerobic phototrophy (*pufABLM*). No genes for flagellar assembly and chemotaxis were annotated. Pathways for cyanate degradation, thiosulfate oxidation (Sox pathway), glycolate oxidation and the biosynthesis of all amino acids except for aspartate were predicted. Further, pathways for thiamine, riboflavin, NAD, pantothenate, coenzyme A, biotin, THF, ubiquinone, and heme biosynthesis were identified. The closest cultivated relative is *Polynucleobacter sp.* MWH-UH24A (GCF_018687475.1), with an average amino acid identity of 82.7% and average nucleotide identity of 74.9%. Current GTDB classification (R220): d__Bacteria; p__Pseudomonadota; c__Gammaproteobacteria; o__Burkholderiales; f__Burkholderiaceae; g__Polynucleobacter; s__Polynucleobacter sp027486235.

Classification

Bacteria » *Pseudomonadota* » *Betaproteobacteria* » *Burkholderiales* » *Burkholderiaceae* » *Polynucleobacter* » *Polynucleobacter hoetzingerianus*

Registry URL

https://seqco.de/i:48717

Species *Polynucleobacter hahnii*

Etymology

[hah.ni'i] **L. masc. gen. n.** *hahnii*, of Hahn, named after the scientist Martin W. Hahn, who isolated and described many species of the genus Polynucleobacter.

Nomenclatural type

NCBI Assembly: GCA_965234415.1 [Ts]

Reference Strain

Strain sc|0038961: ZE-4

Description

Type strain is *Polynucleobacter hahnii* ZE-4 (GCA_965234415.1), isolated from 5 m depth from Lake Zurich, Switzerland (date: 2019-04-03), *via* high-throughput dilution to extinction cultivation. ZE-4 has a genome size of 2.3 Mbp with a genomic GC content of 43.7%, contains 3 rRNA genes and 38 tRNAs. The genome is complete, consisting of a circular chromosome. The genome contains genes encoding anoxygenic aerobic phototrophy (*pufABLM*). No genes for flagellar assembly and chemotaxis were annotated. Pathways for nitrate reduction, thiosulfate oxidation (Sox pathway), glycolate oxidation and the biosynthesis of all amino acids except for aspartate were predicted. Further, pathways for thiamine, riboflavin, NAD, pantothenate, coenzyme A, biotin, THF, ubiquinone, and heme biosynthesis were identified. The closest cultivated relative is *Polynucleobacter* sp. IMCC 30228 (GCF_021395205.1), with an average amino acid identity of 86.6% and average nucleotide identity of 82.8%. Current GTDB classification (R220): d__Bacteria; p__Pseudomonadota; c__Gammaproteobacteria; o__Burkholderiales; f__Burkholderiaceae; g__Polynucleobacter; s__Polynucleobacter sp903944725.

Classification

Bacteria » *Pseudomonadota* » *Betaproteobacteria* » *Burkholderiales* » *Burkholderiaceae* » *Polynucleobacter* » *Polynucleobacter hahnii*

Registry URL

https://seqco.de/i:48716

Genus *Pernthalerella*

Etymology

[Pern.tha.le.re'lla] **N.L. fem. dim. n.** *Pernthalerella*, Named after Austrian scientist Jakob Pernthaler, who greatly contributed to the field of aquatic microbial ecology.

Nomenclatural type

Species *Pernthalerella aquatica*^{Ts}

Description

The genus contains three species, *Pernthalerella aquatica* (GCA_965194445.1), *Pernthalerella lacunae* (GCA_965194545.1), and *Pernthalerella communis* (GCA_965194595.1) that were isolated from freshwater lakes. Type species is *Pernthalerella aquatica* GE-M3 (GCA_965194445.1). The closest cultivated relative is *Paucimonas lemoignei* DSM 7445 (GCF_004342585.1) with average amino acid identities of 64-64.5% and average nucleotide identities of 69.1-71%. SILVA classification of 16S rRNA genes is Bacteria/Proteobacteria/Gammaproteobacteria/Burkholderiales/Oxalobacteraceae/Noviherbaspirillum_3. Current GTDB classification (R220): d__Bacteria; p__Pseudomonadota; c__Gammaproteobacteria; o__Burkholderiales; f__Burkholderiaceae; g__SYFN01.

Classification

Bacteria » *Pseudomonadota* » *Betaproteobacteria* » *Burkholderiales* » *Burkholderiaceae* » *Pernthalerella*

Registry URL

https://seqco.de/i:49109

Species *Pernthalerella aquatica*^{Ts}

Etymology

[a.qua.ti'ca] **L. fem. adj.** *aquatica*, living or found in the water.

Nomenclatural type

NCBI Assembly: GCA_965194445.1 ^{Ts}

Reference Strain

Strain sc|0038788: GE-M3

Description

Type species is *Pernthalerella aquatica* GE-M3 (GCA_965194445.1), isolated from 5 m depth from Greifensee, Switzerland (date: 2019-04-03), *via* high-throughput dilution to extinction cultivation. GE-M3 has a genome size of 3.1 Mbp with a genomic GC content of 53.8%, contains 3 rRNA genes and 41 tRNAs. The genome is a high-quality draft consisting of 7 contigs. The genome contains genes encoding anoxygenic aerobic phototrophy (*pufABLM*) and the complete Calvin cycle for carbon fixation via RuBisCO. Genes for flagellar and pilus assembly were annotated. Pathways for urea degradation, glycolate oxidation and the biosynthesis of all amino acids were predicted. Further, pathways for thiamine, riboflavin, NAD, pantothenate, coenzyme A, biotin, THF, and heme biosynthesis were identified. The closest cultivated relative is *Paucimonas lemoignei* DSM 7445 (GCF_004342585.1) with an average amino acid identity of 64.5% and average nucleotide identity of 71.1% and two other newly proposed species, *Pernthalerella lacunae* MaE-M21 (GCA_965194545.1) and *Pernthalerella communis* MsE-6 (GCA_965194595.1), with AAI of 72.1-73.1% and ANI of 71.9-72.5%. Current GTDB classification (R220): d__Bacteria; p__Pseudomonadota; c__Gammaproteobacteria; o__Burkholderiales; f__Burkholderiaceae; g__SYFN01; s__.

Classification

Bacteria » *Pseudomonadota* » *Betaproteobacteria* » *Burkholderiales* » *Burkholderiaceae* » *Pernthalerella* » *Pernthalerella aquatica*^{Ts}

Registry URL

https://seqco.de/i:49110

Species *Pernthalerella lacunae*

Etymology

[la.cu'na.e] **L. fem. gen. n.** *lacunae*, of a ditch, pit, hole, pool, or pond, referring to the isolation source of the type from a freshwater lake.

Nomenclatural type

NCBI Assembly: GCA_965194545.1 ^{Ts}

Reference Strain

Strain sc|0038789: MaE-M21

Description

Type species is *Pernthalerella lacunae* MaE-M21 (GCA_965194545.1), isolated from 5 m depth from Lake Maggiore, Italy (date: 2019-11-04), *via* high-throughput dilution to extinction cultivation. MaE-M21 has a genome size of 2.8 Mbp with a genomic GC content of 54.9%, contains 3 rRNA genes and 45 tRNAs. The genome is a high-quality draft consisting of 7 contigs. The genome contains genes encoding anoxygenic aerobic phototrophy (*pufABLM*). Genes for flagellar and pilus assembly were annotated. Pathways for benzene degradation, glycolate oxidation and the biosynthesis of all amino acids were predicted. Further, pathways for thiamine, riboflavin, NAD, pantothenate, coenzyme A, biotin, THF, and heme biosynthesis were identified. The closest cultivated relative is *Paucimonas lemoignei* DSM 7445 (GCF_004342585.1) with an average amino acid identity of 64.16% and average nucleotide identity of 70.49% and two other newly proposed species, *Pernthalerella aquatica* GE-M3 (GCA_965194445.1) and *Pernthalerella communis* MsE-6 (GCA_965194595.1), with AAI of 72.1-73.1% and ANI of 71.9-72.5%. Current GTDB classification (R220): d__Bacteria; p__Pseudomonadota; c__Gammaproteobacteria; o__Burkholderiales; f__Burkholderiaceae; g__SYFN01; s__SYFN01 sp021299035.

Classification

Bacteria » *Pseudomonadota* » *Betaproteobacteria* » *Burkholderiales* » *Burkholderiaceae* » *Pernthalerella* » *Pernthalerella lacunae*

Registry URL

https://seqco.de/i:49111

Species *Pernthalerella communis*

Etymology

[com.mu'nis] **L. fem. adj.** *communis*, common

Nomenclatural type

NCBI Assembly: GCA_965194595.1 ^{Ts}

Reference Strain

Strain sc|0038790: MsE-6

Description

Type species is *Pernthalerella communis* MsE-6 (GCA_965194595.1), isolated from 5 m depth from Lake Most, Czechia (date: 2019-04-30), *via* high-throughput dilution to extinction cultivation. MsE-6 has a genome size of 3 Mbp with a genomic GC content of 47.7%, contains 3 rRNA genes and 37 tRNAs. The genome is a high-quality draft consisting of 27 contigs. The genome contains genes encoding anoxygenic aerobic phototrophy (*pufABLM*) and the complete Calvin cycle for carbon fixation via RuBisCO. Genes for flagellar and pilus assembly were

annotated. Pathways for salicylate degradation, glycolate oxidation and the biosynthesis of all amino acids were predicted. Further, pathways for thiamine, riboflavin, NAD, pantothenate, coenzyme A, biotin, THF, and heme biosynthesis were identified. The closest cultivated relative is *Paucimonas lemoignei* DSM 7445 (GCF_004342585.1) with an average amino acid identity of 64.02% and average nucleotide identity of 69.11% and two other newly proposed species, *Pernthalerella lacunae* MaE-M21 (GCA_965194545.1) and *Pernthalerella aquatica* GE-M3 (GCA_965194445.1), with AAI of 72.1-73.1% and ANI of 71.9-72.5%. Current GTDB classification (R220): d__Bacteria; p__Pseudomonadota; c__Gammaproteobacteria; o__Burkholderiales; f__Burkholderiaceae; g__SYFN01; s__SYFN01 sp903846425.

Classification
: *Bacteria* » *Pseudomonadota* » *Betaproteobacteria* » *Burkholderiales* » *Burkholderiaceae* » *Pernthalerella* » *Pernthalerella communis*

Registry URL
: https://seqco.de/i:49112

Genus *Hahnella*

Etymology
: [Hah.ne'lla] **N.L. fem. dim. n.** *Hahnella*, named after the scientist Martin W. Hahn, a pioneer in cultivating important freshwater bacteria; he also isolated the first strains of this genus (MWH-UniP1) and closely related taxa.

Nomenclatural type
: Species *Hahnella aquatica*^{Ts}

Description
: Description: A genus of abundant freshwater bacteria, commonly known from metagenomic and 16S rRNA amplicon studies, often referred to MWH-UniP1 group or betaVI in 16S rRNA based studies (PMCID: PMC3063352, 31919153). It contains two species: *Hahnella aquatica* (MiE-11, GCA_965194505.1) and *Hahnella lacustris* (MsE-M47, GCA_965194655.1), both isolated from freshwater lakes. Type species is *Hahnella aquatica* MiE-11 (GCA_965194505.1). The closest cultivated relative is the undescribed *Burkholderiales* bacterium LSUCC0115 (GCA_009646425.1), with an average amino acid identity of 67-67.3% and average nucleotide identity of 70.6-71.7%. The closest validly described relative is *Limnobacter thiooxidans* CS-K2 (GCF_036323495.1), with AAIs of 53.6-54.4% and ANIs of 66.4-66.9%. Current GTDB classification (R220): d__Bacteria; p__Pseudomonadota; c__Gammaproteobacteria; o__Burkholderiales; f__Burkholderiaceae_A; g__UBA2463.

Classification
: *Bacteria* » *Pseudomonadota* » *Betaproteobacteria* » *Burkholderiales* » *Burkholderiaceae* » *Hahnella*

Registry URL
: https://seqco.de/i:49113

Species *Hahnella aquatica*^{Ts}

Etymology
: [a.qua.ti'ca] **L. fem. adj.** *aquatica*, living or found in the water.

Nomenclatural type
: NCBI Assembly: GCA_965194505.1 ^{Ts}

Reference Strain
: Strain sc|0039566: MiE-11

Description

Type species is *Hahnella aquatica* MiE-11 (GCA_965194505.1), isolated from 5 m depth from Lake Milada, Czechia (date: 2019-07-23), *via* high-throughput dilution to extinction cultivation. MiE-11 has a genome size of 1.8 Mbp with a genomic GC content of 61.8%, contains 3 rRNA genes and 43 tRNAs. The genome is complete, consisting of a circular chromosome. The genome contains genes encoding anoxygenic aerobic phototrophy (*pufABLM*). Genes for flagellar assembly were annotated. Pathways for thiosulfate oxidation (Sox pathway), glycolate oxidation, and the biosynthesis of all amino acids were predicted. Further, pathways for thiamine, riboflavin, NAD, pantothenate, coenzyme A, THF, and heme biosynthesis were identified. The closest cultivated relatives are the undescribed *Burkholderiales* bacterium LSUCC0115 (GCA_009646425.1), with an average amino acid identity of 67.3% and average nucleotide identity of 71.7% and another newly proposed species, *Hahnella lacustris* MsE-M47 (GCA_965194655.1), with an AAI of 65.8% and an ANI of 70.4%. The closest validly described relative is *Limnobacter thiooxidans* CS-K2 (GCF_036323495.1) with an AAI of 54.41% and an ANI of 66.9%. Current GTDB classification (R220): d__Bacteria; p__Pseudomonadota; c__Gammaproteobacteria; o__Burkholderiales; f__Burkholderiaceae_A; g__UBA2463; s__.

Classification

Bacteria » *Pseudomonadota* » *Betaproteobacteria* » *Burkholderiales* » *Burkholderiaceae* » *Hahnella* » *Hahnella aquatica*[Ts]

Registry URL

https://seqco.de/i:49114

Species *Hahnella lacustris*

Etymology

[la.cus'tris] **N.L. fem. adj.** *lacustris*, pertaining to a lake

Nomenclatural type

NCBI Assembly: GCA_965194655.1 [Ts]

Reference Strain

Strain sc|0038792: MsE-M47

Description

Type species is *Hahnella lacustris* MsE-M47 (GCA_965194655.1), isolated from 5 m depth from Lake Most, Czechia (date: 2019-10-01), *via* high-throughput dilution to extinction cultivation. MsE-M47 has a genome size of 2.2 Mbp with a genomic GC content of 59.2%, contains 3 rRNA genes and 42 tRNAs. The genome is complete, consisting of a circular chromosome. The genome is complete, consisting of a circular chromosome. The genome contains genes encoding anoxygenic aerobic phototrophy (*pufABLM*) and the complete Calvin cycle for carbon fixation via RuBisCO. Genes for flagellar assembly and chemotaxis were annotated. Pathways for taurine degradation, thiosulfate oxidation (Sox pathway), glycolate oxidation, and the biosynthesis of all amino acids were predicted. Further, pathways for riboflavin, NAD, pantothenate, coenzyme A, biotin, THF, and heme biosynthesis were identified. The closest cultivated relative is the undescribed *Burkholderiales* bacterium LSUCC0115 (GCA_009646425.1), with an average amino acid identity of 67% and average nucleotide identity of 70.6% and another newly proposed species, *Hahnella aquatica* MiE-11 (GCA_965194505.1), with an AAI of 65.8% and an ANI of 70.4%. The closest validly described relative is *Limnobacter thiooxidans* CS-K2 (GCF_036323495.1) with an AAI of 53.55% and an ANI of 66.44%. Current GTDB classification (R220): d__Bacteria; p__Pseudomonadota; c__Gammaproteobacteria; o__Burkholderiales; f__Burkholderiaceae_A; g__UBA2463; s__UBA2463 sp945862985.

Classification

Bacteria » *Pseudomonadota* » *Betaproteobacteria* » *Burkholderiales* » *Burkholderiaceae* » *Hahnella* » *Hahnella lacustris*

Registry URL

https://seqco.de/i:49117

Genus *Lacustribacter*

Etymology

[La.cus.tri.bac'ter] **N.L. masc. adj.** *lacustris*, belonging to a lake; **N.L. masc. n.** *bacter*, rod; **N.L. masc. n.** *Lacustribacter*, A rod-shaped bacterium living in freshwaters.

Nomenclatural type

Species *Lacustribacter communis*^{Ts}

Description

A genus of abundant freshwater bacteria, commonly known from metagenomic and 16S rRNA amplicon studies, often referred to MWH-UniP1 group or betaVI in 16S rRNA based studies. Type species is *Lacustribacter communis* MsE-M52 (GCA_965194335.1). The closest cultivated relatives are the undescribed *Burkholderiales* bacterium LSUCC0115 (GCA_009646425.1), with an average amino acid identity of 61.9% and average nucleotide identity of 67.9% and the newly established genus *Hahnella,* with 60.4-61.7% AAI and 67.2-67.9% ANI. The closest validly described relative is *Limnobacter thiooxidans* CS-K2 (GCF_036323495.1) with an AAI of 53.95% and an ANI of 66.33%. Current GTDB classification (R220): d__Bacteria; p__Pseudomonadota; c__Gammaproteobacteria; o__Burkholderiales; f__Burkholderiaceae; g__UBA954.

Classification

Bacteria » *Pseudomonadota* » *Betaproteobacteria* » *Burkholderiales* » *Burkholderiaceae* » *Lacustribacter*

Registry URL

https://seqco.de/i:48710

Species *Lacustribacter communis*^{Ts}

Etymology

[com.mu'nis] **L. masc. adj.** *communis*, common; referring to a wide distribution in freshwater lakes.

Nomenclatural type

NCBI Assembly: GCA_965194335.1 ^{Ts}

Reference Strain

Strain sc|0038962: MsE-M52

Description

Type strain is *Lacustribacter communis* MsE-M52 (GCA_965194335.1), isolated from 5 m depth from Lake Most, Czechia (date: 2019-10-01), *via* high-throughput dilution to extinction cultivation. MsE-M52 has a genome size of 2.1 Mbp with a genomic GC content of 53.3%, contains 3 rRNA genes and 42 tRNAs. The genome is complete, consisting of a circular chromosome. The genome contains genes encoding anoxygenic aerobic phototrophy (*pufABLM*). Genes for flagellar assembly were annotated. Pathways for urea and taurine degradation, thiosulfate oxidation (Sox pathway), glycolate oxidation, and the biosynthesis of all amino acids except for aspartate were predicted. Further, pathways for thiamine, riboflavin, NAD, pantothenate, coenzyme A, THF, and heme biosynthesis were identified. The closest cultivated relatives are the undescribed *Burkholderiales* bacterium LSUCC0115 (GCA_009646425.1), with an average amino acid identity of 61.9% and average nucleotide identity of 67.9% and the newly established genus *Hahnella,* with 60.4-61.7% AAI and 67.2-67.9% ANI. The closest validly described relative is *Limnobacter thiooxidans* CS-K2 (GCF_036323495.1) with an AAI of 53.95% and an ANI of 66.33%. Current GTDB classification (R220): d__Bacteria; p__Pseudomonadota; c__Gammaproteobacteria; o__Burkholderiales; f__Burkholderiaceae; g__UBA954; s__UBA954 sp002293155.

Classification

Bacteria » *Pseudomonadota* » *Betaproteobacteria* » *Burkholderiales* » *Burkholderiaceae* » *Lacustribacter* » *Lacustribacter communis*[Ts]

Registry URL

https://seqco.de/i:48709

Species *Hydrogenophaga miladensis*

Etymology

[mi.la.den'sis] **N.L. fem. adj.** *miladensis*, pertaining to Lake Milada (Czechia), the isolation source of the type strain.

Nomenclatural type

NCBI Assembly: GCA_965234585.1 [Ts]

Reference Strain

Strain sc|0038963: MiE-M28

Description

Type strain is *Hydrogenophaga miladensis* MiE-M28 (GCA_965234585.1), isolated from 5 m depth from Lake Milada, Czechia (date: 2019-10-15), *via* high-throughput dilution to extinction cultivation. MiE-M28 has a genome size of 2.4 Mbp with a genomic GC content of 60.6%, contains 6 rRNA genes and 43 tRNAs. The genome is complete, consisting of a circular chromosome. The genome contains genes encoding anoxygenic aerobic phototrophy (*pufABLM*) and the complete Calvin cycle for carbon fixation via RuBisCO. Genes for flagellar and pilus assembly were annotated. Pathways for thiosulfate oxidation (Sox pathway), glycolate and methane/alkanesulfonate oxidation and the biosynthesis of all amino acids were predicted. Further, pathways for thiamine, riboflavin, NAD, coenzyme A, pimeloyl-ACP, and heme biosynthesis were identified. The closest cultivated relative is *Hydrogenophaga intermedia* MER 62 (GCF_001571205.1), with an average nucleotide identity of 74.2%. Current GTDB classification (R220): d__Bacteria; p__Pseudomonadota; c__Gammaproteobacteria; o__Burkholderiales; f__Burkholderiaceae_B; g__Hydrogenophaga; s__Hydrogenophaga intermedia_C.

Classification

Bacteria » *Pseudomonadota* » *Betaproteobacteria* » *Burkholderiales* » *Comamonadaceae* » *Hydrogenophaga* » *Hydrogenophaga miladensis*

Registry URL

https://seqco.de/i:48728

Species *Limnohabitans rimovensis*

Etymology

[ri.mo.ven'sis] **N.L. masc. adj.** *rimovensis*, pertaining to the Římov Reservoir, Czech Republic, the isolation source of the species.

Nomenclatural type

NCBI Assembly: GCA_965234775.1 [Ts]

Reference Strain

Strain sc|0038964: RE-1

Description

Type strain is *Limnohabitans rimovensis* RE-1 (GCA_965234775.1), isolated from 0.5 m depth from the Římov Reservoir, Czechia (date: 2019-04-23), *via* high-throughput dilution to extinction cultivation. RE-1 has a genome size of 3.5 Mbp with a genomic GC content of 58.7%, contains 3 rRNA genes and 43 tRNAs. The genome is a high-quality draft consisting of 12 contigs. The genome contains genes encoding anoxygenic aerobic phototrophy (*pufABLM*). Genes for flagellar and pilus assembly were annotated. Pathways for urea degradation, thiosulfate oxidation (Sox pathway), methane/alkanesulfonate and glycolate oxidation, benzoate, salicylate, and phthalate degradation, and the biosynthesis of all amino acids except for aspartate were predicted. Further,

pathways for thiamine, riboflavin, pantothenate, coenzyme A, THF, and heme biosynthesis were identified. The closest cultivated relative is *Limnohabitans* sp. JirII-31 (GCF_002778315.1), with an average amino acid identity of 65.1% and average nucleotide identity of 72.4%. Current GTDB classification (R220): d__Bacteria; p__Pseudomonadota; c__Gammaproteobacteria; o__Burkholderiales; f__Burkholderiaceae_B; g__Limnohabitans; s__.

Classification

Bacteria » *Pseudomonadota* » *Betaproteobacteria* » *Burkholderiales* » *Comamonadaceae* » *Limnohabitans* » *Limnohabitans rimovensis*

Registry URL

https://seqco.de/i:48738

Species *Limnohabitans simekii*

Etymology

[si.me.ki'i] **N.L. masc. gen. n.** *simekii*, From Simek, named after the Czech scientist Karel Šimek, who first recognized the importance of the genus Limnohabitans and was involved in the description of the genus.

Nomenclatural type

NCBI Assembly: GCA_965234735.1 [Ts]

Reference Strain

Strain sc|0038785: MiE-M12

Description

Type strain is *Limnohabitans simekii* MiE-M12 (GCA_965234735.1), isolated from 5 m depth from Lake Milada, Czechia (date: 2019-04-16), *via* high-throughput dilution to extinction cultivation. MiE-M12 has a genome size of 2.7 Mbp with a genomic GC content of 55.8 %, contains 3 rRNA genes and 37 tRNAs. The genome is a high-quality draft consisting of 9 contigs. The genome contains genes encoding anoxygenic aerobic phototrophy (*pufABLM*) and the complete Calvin cycle for carbon fixation via RuBisCO. Genes for flagellar and pilus assembly were annotated. Pathways for urea degradation, thiosulfate oxidation (Sox pathway), glycolate oxidation, and the biosynthesis of all amino acids were predicted. Further, pathways for thiamine, riboflavin, coenzyme A, biotin, THF, and heme biosynthesis were identified. The closest cultivated relative is *Limnohabitans* sp. Jir61 (GCF_003063545.1) with an average amino acid identity of 66.2% and average nucleotide identity of 72.6%. Current GTDB classification (R220): d__Bacteria; p__Pseudomonadota; c__Gammaproteobacteria; o__Burkholderiales; f__Burkholderiaceae_B; g__Limnohabitans; s__Limnohabitans sp937891045.

Classification

Bacteria » *Pseudomonadota* » *Betaproteobacteria* » *Burkholderiales* » *Comamonadaceae* » *Limnohabitans* » *Limnohabitans simekii*

Registry URL

https://seqco.de/i:44274

Species *Limnohabitans kasalickyi*

Etymology

[ka.sa.li.cky'i] **N.L. masc. gen. n.** *kasalickyi*, named after the Czech scientist Vojtěch (Vojta) Kasalický, who isolated many strains of the genus Limnohabitans and was involved in the description of the genus.

Nomenclatural type

NCBI Assembly: GCA_965234325.1 [Ts]

Reference Strain

Strain sc|0038965: MaE-M4

Description

Type strain is *Limnohabitans kasalickyi* MaE-M4 (GCA_965234325.1), isolated from 5 m depth from Lake Maggiore, Italy (date: 2019-04-09), *via* high-throughput dilution to extinction cultivation. MaE-M4 has a genome size of 3.7 Mbp with a genomic GC content of 56.3%, contains 6 rRNA genes and 44 tRNAs. The genome is a high-quality draft consisting of 16 contigs. The genome contains genes encoding anoxygenic aerobic phototrophy (*pufABLM*). Genes for flagellar and pilus assembly were annotated. Pathways for cyanate, urea, and taurine degradation, thiosulfate oxidation (Sox pathway), methane/alkanesulfonate and glycolate oxidation, and the biosynthesis of all amino acids except for aspartate were predicted. Further, pathways for thiamine, riboflavin, pantothenate, coenzyme A, pimeloyl-ACP, THF, and heme biosynthesis were identified. The closest cultivated relative is *Limnohabitans* sp. Jir61 (GCF_003063545.1), with an average amino acid identity of 77.9% and average nucleotide identity of 80.1%. Current GTDB classification (R220): d__Bacteria; p__Pseudomonadota; c__Gammaproteobacteria; o__Burkholderiales; f__Burkholderiaceae_B; g__Limnohabitans; s__Limnohabitans sp002778325.

Classification

Bacteria » *Pseudomonadota* » *Betaproteobacteria* » *Burkholderiales* » *Comamonadaceae* » *Limnohabitans* » *Limnohabitans kasalickyi*

Registry URL

https://seqco.de/i:48719

Genus *Allorhodoferax*

Etymology

[A.llo.rho.do.fe'rax] **Gr. masc. pron.** *allos*, another, other, different; **N.L. masc. n.** *rhodoferax*, a bacterial genus name; **N.L. masc. n.** *Allorhodoferax*, another Rhodoferax.

Nomenclatural type

Species *Allorhodoferax aquaticus*^{Ts}

Description

The genus contains two species, *Allorhodoferax aquaticus* (GCA_965194715.1) and *Allorhodoferax lacus* (GCA_965194635.1), both isolated from freshwater lakes. Type species is *Allorhodoferax aquaticus* MsE-M22 (GCA_965194715.1). The genus is classified as d__Bacteria; p__Pseudomonadota; c__Gammaproteobacteria; o__Burkholderiales; f__Burkholderiaceae; Rhodoferax_C in GTDB (R220) and contains bacteria with various names such as *Curvibacter, Rhodoferax*, etc. The closest cultivated relative is *Curvibacter* sp. AEP1-3 (GCF_002163715.1), with average amino acid identities of 86.2-90.7% and average nucleotide identities of 81-9-86.6%. The type strain of the genus *Rhodoferax* (*Rhodoferax fermentans* JCM 7819; GCA_002017865.1) is classified as d__Bacteria; p__Pseudomonadota; c__Gammaproteobacteria; o__Burkholderiales; f__Burkholderiaceae; g__Rhodoferax; s__Rhodoferax fermentans, with AAIs of 66.0-66.8% and ANIs of 72.4-72.6%. We therefore suggest a reclassification of this genus (GTDB genus Rhodoferax_C) with the proposed name *Allorhodoferax*.

Classification

Bacteria » *Pseudomonadota* » *Betaproteobacteria* » *Burkholderiales* » *Comamonadaceae* » *Allorhodoferax*

Registry URL

https://seqco.de/i:49941

Species *Allorhodoferax aquaticus*^{Ts}

Etymology

[a.qua'ti.cus] **L. masc. adj.** *aquaticus*, living, growing, or found in or by the water, aquatic

Nomenclatural type

NCBI Assembly: GCA_965194715.1 ^{Ts}

Reference Strain
  [Strain sc|0038967](): MsE-M22
Description
  Type strain is *Allorhodoferax aquaticus* MsE-M22 (GCA_965194715.1), isolated from 5 m depth from Lake Most, Czechia (date: 2019-07-30), *via* high-throughput dilution to extinction cultivation. MsE-M22 has a genome size of 3.7 Mbp with a genomic GC content of 59.3%, contains 6 rRNA genes and 47 tRNAs. The genome is complete, consisting of a circular chromosome. Genes for flagellar and pilus assembly were annotated. Pathways for nitrate reduction, cyanate and urea degradation, glycolate oxidation and the biosynthesis of all amino acids except for aspartate were predicted. Further, pathways for thiamine, riboflavin, NAD, coenzyme A, THF, cobalamin, and heme biosynthesis were identified. The closest cultivated relative is *Curvibacter* sp. AEP1-3 (GCF_002163715.1), with an average amino acid identity of 90.7% and average nucleotide identity of 86.6%. Current GTDB classification (R220): d__Bacteria; p__Pseudomonadota; c__Gammaproteobacteria; o__Burkholderiales; f__Burkholderiaceae; g__Rhodoferax_C; s__Rhodoferax_C sp027488985.
Classification
  Bacteria » *Pseudomonadota* » *Betaproteobacteria* » *Burkholderiales* » *Comamonadaceae* » *Allorhodoferax* » *Allorhodoferax aquaticus*^{Ts}
Registry URL
  https://seqco.de/i:48704

Species *Allorhodoferax lacus*
Etymology
  [la'cus] **L. gen. n.** *lacus*, of a lake, referring to the habitat from which the type strain was isolated.
Nomenclatural type
  NCBI Assembly: GCA_965194635.1 ^{Ts}
Reference Strain
  Strain sc|0038966: ZE-M1
Description
  Type strain is *Allorhodoferax lacus* ZE-M1 (GCA_965194635.1), isolated from 5 m depth from Lake Zurich, Switzerland (date: 2019-04-03), *via* high-throughput dilution to extinction cultivation. ZE-M1 has a genome size of 3.7 Mbp with a genomic GC content of 60.1%, contains 6 rRNA genes and 46 tRNAs. The genome is a high-quality draft consisting of 2 contigs. Genes for flagellar and pilus assembly were annotated. Pathways for cyanate and urea degradation, glycolate and methane/alkanesulfonate oxidation and the biosynthesis of all amino acids except for aspartate were predicted. Further, pathways for thiamine, riboflavin, NAD, coenzyme A, THF, cobalamin, and heme biosynthesis were identified. The closest cultivated relative is *Curvibacter* sp. AEP1-3 (GCF_002163715.1), with an average amino acid identity of 86.2% and average nucleotide identity of 81.9%. Current GTDB classification (R220): d__Bacteria; p__Pseudomonadota; c__Gammaproteobacteria; o__Burkholderiales; f__Burkholderiaceae; g__Rhodoferax_C; s__.
Classification
  Bacteria » *Pseudomonadota* » *Betaproteobacteria* » *Burkholderiales* » *Comamonadaceae* » *Allorhodoferax* » *Allorhodoferax lacus*
Registry URL
  https://seqco.de/i:48720

Species *Zwartia planktonica*
Etymology
  [plan.kto.ni'ca] **N.L. fem. adj.** *planktonica*, living in the plankton of lakes, planktonic; from Gr. masc. adj. planktos, wandering.

Nomenclatural type

NCBI Assembly: GCA_965234315.1 Ts

Reference Strain

Strain sc|0038969: RE-10

Description

Type strain is *Zwartia planktonica* RE-10 (GCA_965234315.1), isolated from 0.5 m depth from the Římov Reservoir, Czechia (date: 2019-04-23), *via* high-throughput dilution to extinction cultivation. RE-10 has a genome size of 4.5 Mbp with a genomic GC content of 52%, contains 6 rRNA genes and 44 tRNAs. The genome is complete, consisting of a circular chromosome. No genes for flagellar or pilus assembly and chemotaxis were annotated. Pathways for cyanate, urea and taurine degradation, thiosulfate oxidation (Sox pathway), glycolate oxidation, and the biosynthesis of all amino acids were predicted. Further, pathways for thiamine, riboflavin, NAD, pantothenate, coenzyme A, THF, ubiquinone, and heme biosynthesis were identified. The closest cultivated relative is *Zwartia hollandica* LF4-65 (GCF_019923725.1), with an average amino acid identity of 71.2% and average nucleotide identity of 71.6%. Current GTDB classification (R220): d__Bacteria; p__Pseudomonadota; c__Gammaproteobacteria; o__Burkholderiales; f__Burkholderiaceae; g__Zwartia; s__Zwartia sp030832095.

Classification

Bacteria » *Pseudomonadota* » *Betaproteobacteria* » *Burkholderiales* » *Alcaligenaceae* » *Zwartia* » *Zwartia planktonica*

Registry URL

https://seqco.de/i:48732

Species *Zwartia lucis*

Etymology

[lu'cis] **L. fem. adj.** *lucis*, of light, referring to the presence of light driven proton pumps (rhodopsins) in the type strain.

Nomenclatural type

NCBI Assembly: GCA_965234455.1 Ts

Reference Strain

Strain sc|0038968: GE-14

Description

Type strain is *Zwartia lucis* GE-14 (GCA_965234455.1), isolated from 5 m depth from Greifensee, Switzerland (date: 2019-04-03), *via* high-throughput dilution to extinction cultivation. GE-14 has a genome size of 3.7 Mbp with a genomic GC content of 50.9%, contains 6 rRNA genes and 41 tRNAs. The genome is a high-quality draft consisting of 7 contigs. In contrast to other *Zwartia* sp., the genome contains genes encoding rhodopsins and the biosynthetic pathway for retinal biosynthesis. No genes for flagellar or pilus assembly and chemotaxis were annotated. Pathways for nitrate reduction, cyanate, urea and taurine degradation, thiosulfate oxidation (Sox pathway), glycolate and methane/alkanesulfonate oxidation, and the biosynthesis of all amino acids were predicted. Further, pathways for thiamine, riboflavin, pantothenate, coenzyme A, THF, ubiquinone, and heme biosynthesis were identified. The closest cultivated relative is *Zwartia panacis* strain KCTC42751 (GCF_030410395.1), with an average amino acid identity of 82.1% and average nucleotide identity of 77.6%. Current GTDB classification (R220): d__Bacteria; p__Pseudomonadota; c__Gammaproteobacteria; o__Burkholderiales; f__Burkholderiaceae; g__Zwartia; s__.

Classification

Bacteria » *Pseudomonadota* » *Betaproteobacteria* » *Burkholderiales* » *Alcaligenaceae* » *Zwartia* » *Zwartia lucis*

Registry URL

https://seqco.de/i:48725

Species *Methylotenera profunda*

Etymology

[pro.fun'da] **L. fem. adj.** *profunda*, deep; referring to the deep zone of lakes where the species is common.

Nomenclatural type

NCBI Assembly: GCA_947054625.1 [Ts]

Reference Strain

Strain sc|0038971: RH-M32

Description

Type strain is *Methylotenera profunda* RH-M32 (GCA_965601725.1), isolated from 30 m depth from the Římov Reservoir, Czechia (date: 15.08.2019), *via* high-throughput dilution to extinction cultivation. RH-M32 has a genome size of 1.7 Mbp with a genomic GC content of 47.9%, contains 6 rRNA genes and 38 tRNAs. The genome is complete, consisting of a circular chromosome. The genome contains genes encoding rhodopsins and the biosynthetic pathway for retinal biosynthesis. No genes for flagellar or pilus assembly and chemotaxis were annotated. Pathways for methanol oxidation (Xox), the RuMP and methylcitric acid (MCA) cycle for methylotrophy and the biosynthesis of all amino acids were predicted. Further, pathways for thiamine, riboflavin, NAD, coenzyme A, pimeloyl-ACP, biotin, THF, ubiquinone, and heme biosynthesis were identified. The closest cultivated relatives are *Methylotenera versatilis* 301 (GCF_000093025.1) with an average amino acid identity of 75.9% and average nucleotide identity of 72.8% and another newly proposed species, *Methylotenera hypolimnetica* RH-M31 (GCA_965601845.1), with an AAI of 94.2% and an ANI of 92%. Current GTDB classification (R220): d__Bacteria; p__Pseudomonadota; c__Gammaproteobacteria; o__Burkholderiales; f__Methylophilaceae; g__Methylotenera; s__Methylotenera oryzisoli_A.

Classification

Bacteria » *Pseudomonadota* » *Betaproteobacteria* » *Nitrosomonadales* » *Methylophilaceae* » *Methylotenera* » *Methylotenera profunda*

Registry URL

https://seqco.de/i:48733

Species *Methylotenera hypolimnetica*

Etymology

[hy.po.lim.ne.ti'ca] **Gr. prep.** *hypo*, below, under; **N.L. fem. adj.** *limnetica*, of a lake; **N.L. fem. adj.** *hypolimnetica*, from the hypolimnion of lakes.

Nomenclatural type

NCBI Assembly: GCA_947054635.1 [Ts]

Reference Strain

Strain sc|0038778: RH-M31

Description

Type strain is *Methylotenera hypolimnetica* RH-M31 (GCA_965601845.1), isolated from 30 m depth from the Římov Reservoir, Czechia (date: 15.08.2019), *via* high-throughput dilution to extinction cultivation. RH-M31 has a genome size of 1.8 Mbp with a genomic GC content of 49.2%, contains 6 rRNA genes and 38 tRNAs. The genome is complete, consisting of a circular chromosome. No genes for flagellar or pilus assembly and chemotaxis were annotated. Pathways for methanol oxidation (Xox), the RuMP and methylcitric acid (MCA) cycle for methylotrophy and the biosynthesis of all amino acids were predicted. Further, pathways for thiamine, riboflavin, NAD, coenzyme A, pimeloyl-ACP, biotin, THF, ubiquinone, and heme biosynthesis were identified. The closest cultivated relatives are *Methylotenera versatilis* 301 (GCF_000093025.1) with an average amino acid identity of 75.7% and average nucleotide identity of 72.6% and another newly proposed species, *Methylotenera profunda* RH-M32 (GCA_965601725.1), with an

AAI of 94.2% and an ANI of 92%. Current GTDB classification (R220): d__Bacteria; p__Pseudomonadota; c__Gammaproteobacteria; o__Burkholderiales; f__Methylophilaceae; g__Methylotenera; s__Methylotenera sp903951385.

Classification

Bacteria » *Pseudomonadota* » *Betaproteobacteria* » *Nitrosomonadales* » *Methylophilaceae* » *Methylotenera* » *Methylotenera hypolimnetica*

Registry URL

https://seqco.de/i:48718

Genus *Novimethylotenera*

Etymology

[No.vi.me.thy.lo.te.ne'ra] **L. adj. masc.** *novus*, new; **N.L. fem. n.** *Methylotenera*, the bacterial genus Methylotenera; **N.L. fem. n.** *Novimethylotenera*, a new Methylotenera

Nomenclatural type

Species *Novimethylotenera aquatica*[Ts]

Description

Type species is *Novimethylotenera aquatica* ME-M6 (GCA_965601485.1). Although the closest cultivated relative (*Methylotenera versatilis* 7; GCF_000799165.1) has an average amino acid identity of 80.36% and average nucleotide identity of 76.56%, the genus *Methylotenera* is known to be polyphyletic in phylogenomic trees (e.g., Salcher et al. 2019; https://doi.org/10.1038/s41396-019-0471-3) and some strains that were previously termed *Methylotenera* should be reclassified. Current GTDB classification of the genus including the closest cultivated relative (GCF_000799165.1) is d__Bacteria; p__Pseudomonadota; c__Gammaproteobacteria; o__Burkholderiales; f__Methylophilaceae; g__Methylotenera_A. We propose a reclassification of the GTDB genus Methylotenera_A to *Novimethylotenera*, gen. nov.

Classification

Bacteria » *Pseudomonadota* » *Betaproteobacteria* » *Nitrosomonadales* » *Methylophilaceae* » *Novimethylotenera*

Registry URL

https://seqco.de/i:48702

Species *Novimethylotenera aquatica*[Ts]

Etymology

[a.qua.ti'ca] **L. fem. adj.** *aquatica*, living or found in the water.

Nomenclatural type

NCBI Assembly: GCA_947054645.1 [Ts]

Reference Strain

Strain sc|0038780: ME-M6

Description

Type strain is *Novimethylotenera aquatica* ME-M6 (GCA_965601485.1), isolated from 5 m depth from Lake Medard, Czechia (date: 09.07.2019), *via* high-throughput dilution to extinction cultivation. ME-M6 has a genome size of 2.1 Mbp with a genomic GC content of 41.7%, contains 6 rRNA genes and 37 tRNAs. The genome is complete, consisting of a circular chromosome. The genome contains genes encoding rhodopsins and the biosynthetic pathway for retinal biosynthesis. No genes for flagellar assembly and chemotaxis were annotated. Pathways for methanol oxidation (Xox), the RuMP and methylcitric acid (MCA) cycle for methylotrophy and the biosynthesis of all amino acids were predicted. Further, pathways for thiamine, riboflavin, NAD, coenzyme A, pimeloyl-ACP, biotin, THF, ubiquinone, and heme biosynthesis were identified. The closest cultivated relative is *Methylotenera versatilis* 7 (GCF_000799165.1) with an average amino acid identity of 80.36% and average nucleotide identity of 76.56%. Current GTDB classification (R220): d__Bacteria; p__Pseudomonadota; c__Gammaproteobacteria;

o__Burkholderiales; f__Methylophilaceae; g__Methylotenera_A; s__Methylotenera_A oryzisoli_A.

Classification

Bacteria » *Pseudomonadota* » *Betaproteobacteria* » *Nitrosomonadales* » *Methylophilaceae* » *Novimethylotenera* » *Novimethylotenera aquatica*[Ts]

Registry URL

https://seqco.de/i:48701

Family *Verrucolacustridaceae*

Etymology

[Ver.ru.co.la.cus.tri.da'ce.ae] **N.L. masc. n.** *Verrucolacustris*, referring to the type genus Verrucolacustris; *-aceae*, ending to denote a family; **N.L. fem. pl. n.** *Verrucolacustridaceae*, the Verrucolacustris family

Nomenclatural type

Genus *Verrucolacustris*

Description

Type strain is *Verrucolacustris abundans* MiH-22 (GCA_965194535.1). In contrast to other *Methylacidiphilales* including the only described genus *Methylacidimicrobium* (*Methylacidiphilaceae,* GCF_902143385.2), no genes for C1 metabolism were identified, therefore we suggest the establishment of a new family of non-methanotrophic *Methylacidiphilales*. The closest cultivated relative is *Methylacidimicrobium cyclopophantes* 3B_2 (GCF_902143385.2), with an average amino acid identity of 49.5% and average nucleotide identity of 62.8%. Current GTDB classification (R220): d__Bacteria; p__Verrucomicrobiota; c__Verrucomicrobiae; o__Methylacidiphilales; f__UBA3015.

Classification

Bacteria » *Verrucomicrobiota* » “Methylacidiphilae” » “Methylacidiphilales” » *Verrucolacustridaceae*

Registry URL

https://seqco.de/i:48695

Genus *Verrucolacustris*

Etymology

[Ver.ru.co.la.cus'tris] **N.L. pref.** *Verruco-*, derived from the bacterial phylum Verrucomicrobiota; **L. masc. adj.** *lacustris*, of a lake; **N.L. masc. n.** *Verrucolacustris*, a freshwater genus of Verrucomicrobiota

Nomenclatural type

Species *Verrucolacustris abundans*[Ts]

Description

Type species is *Verrucolacustris abundans* MiH-22 (GCA_965194535.1). The closest cultivated relative is *Methylacidimicrobium cyclopophantes* 3B_2 (GCF_902143385.2), with an average amino acid identity of 49.5% and average nucleotide identity of 62.8%. Current GTDB classification (R220): d__Bacteria; p__Verrucomicrobiota; c__Verrucomicrobiae; o__Methylacidiphilales; f__UBA3015; g__UBA3015.

Classification

Bacteria » *Verrucomicrobiota* » “Methylacidiphilae” » “Methylacidiphilales” » *Verrucolacustridaceae* » *Verrucolacustris*

Registry URL

https://seqco.de/i:48696

Species *Verrucolacustris abundans*[Ts]

Etymology

[a.bun'dans] **L. masc. part. adj.** *abundans*, abundant; referring to high global abundances

Nomenclatural type

NCBI Assembly: GCA_965194535.1 [Ts]

Reference Strain

Strain sc|0038970: MiH-22

Description

Type strain is *Verrucolacustris abundans* MiH-22 (GCA_965194535.1), isolated from 15 m depth from Lake Milada, Czechia (date: 2019-10-15), *via* high-throughput dilution to extinction cultivation. MiH-22 has a genome size of 2.0 Mbp with a genomic GC content of 52.8%, contains 3 rRNA genes and 46 tRNAs. The genome is complete, consisting of a circular chromosome. The genome contains genes encoding rhodopsins. No genes for flagellar assembly and chemotaxis were annotated. In contrast to other *Methylacidiphilales* members, no genes for C1 metabolism (methanotrophy) were identified. Pathways for assimilatory sulfate reduction and the biosynthesis of all amino acids except for methionine, histidine and tryptophan were predicted. Further, pathways for only three vitamins (thiamine, riboflavin, and biotin biosynthesis) were identified. The closest cultivated relative is *Methylacidimicrobium cyclopophantes* 3B_2 (GCF_902143385.2), with an average amino acid identity of 49.5% and average nucleotide identity of 62.8%. Current GTDB classification (R220): d__Bacteria; p__Verrucomicrobiota; c__Verrucomicrobiae; o__Methylacidiphilales; f__UBA3015; g__UBA3015; s__UBA3015 sp030054195.

Classification

Bacteria » *Verrucomicrobiota* » “Methylacidiphilae” » “Methylacidiphilales” » *Verrucolacustridaceae* » *Verrucolacustris* » *Verrucolacustris abundans*[Ts]

Registry URL

https://seqco.de/i:48694

Supplementary Figures

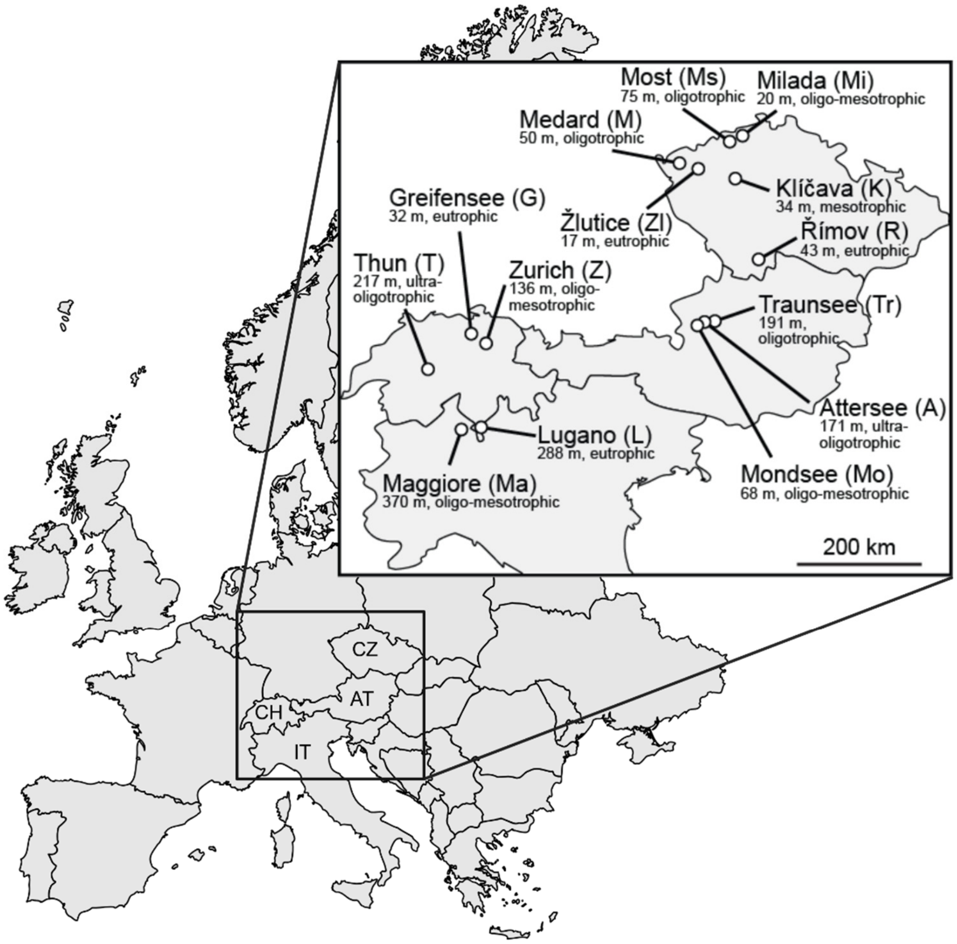

Supplementary Figure 1. Overview of the sampled lakes in the Czech Republic, Austria, Switzerland and Italy. Maximum depth and trophic state are mentioned below the lake name; more information can be found in Supplementary Data 2. Created in BioRender. Salcher, M. (2025) https://BioRender.com/xfgr7mt

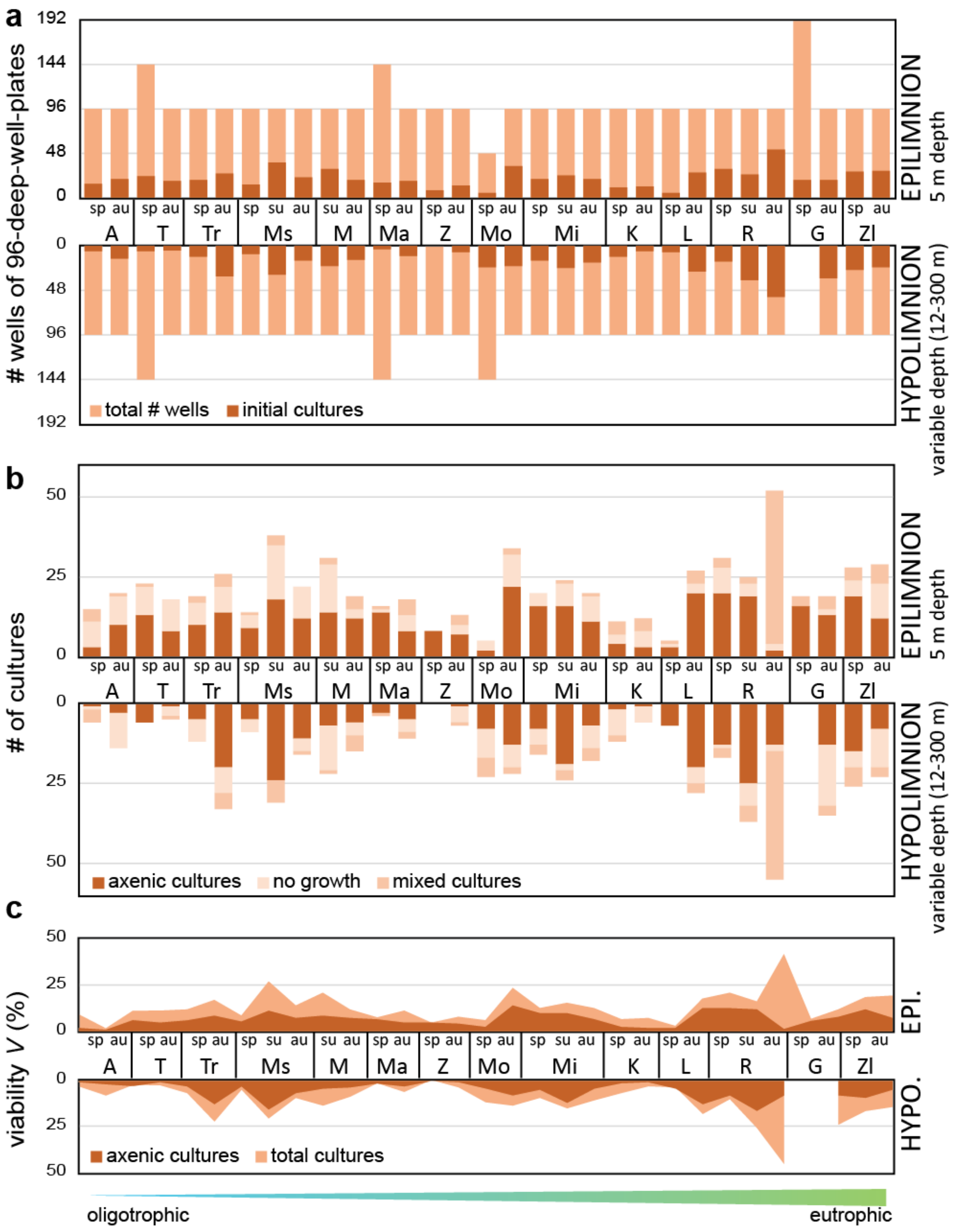

Supplementary Figure 2. Results of dilution-to extinction cultivation experiments for individual samples taken from the epi- and hypolimnion of 14 lakes. a Number of initial cultures (dark orange) and total number of wells inoculated (light orange). **b** Number of axenic cultures (dark orange), cultures with growth arrest (very light orange) and mixed cultures (light orange). **c** Viability *V* (%) of all initial cultures (light orange) and axenic cultures (dark orange). Lakes are sorted from oligo- to eutrophic. Abbreviations: A: Attersee, T: Thun, Tr: Traunsee, Ms: Most, M: Medard, Ma: Maggiore, Z: Zurich, Mo: Mondsee, Mi: Milada, K: Klíčava, L: Lugano, R: Římov, G: Greifensee, Zl: Žlutice, sp: spring; su: summer; au: autumn. EPI: epilimnion; HYPO: hypolimnion. Raw data is given in Supplementary Data 3.

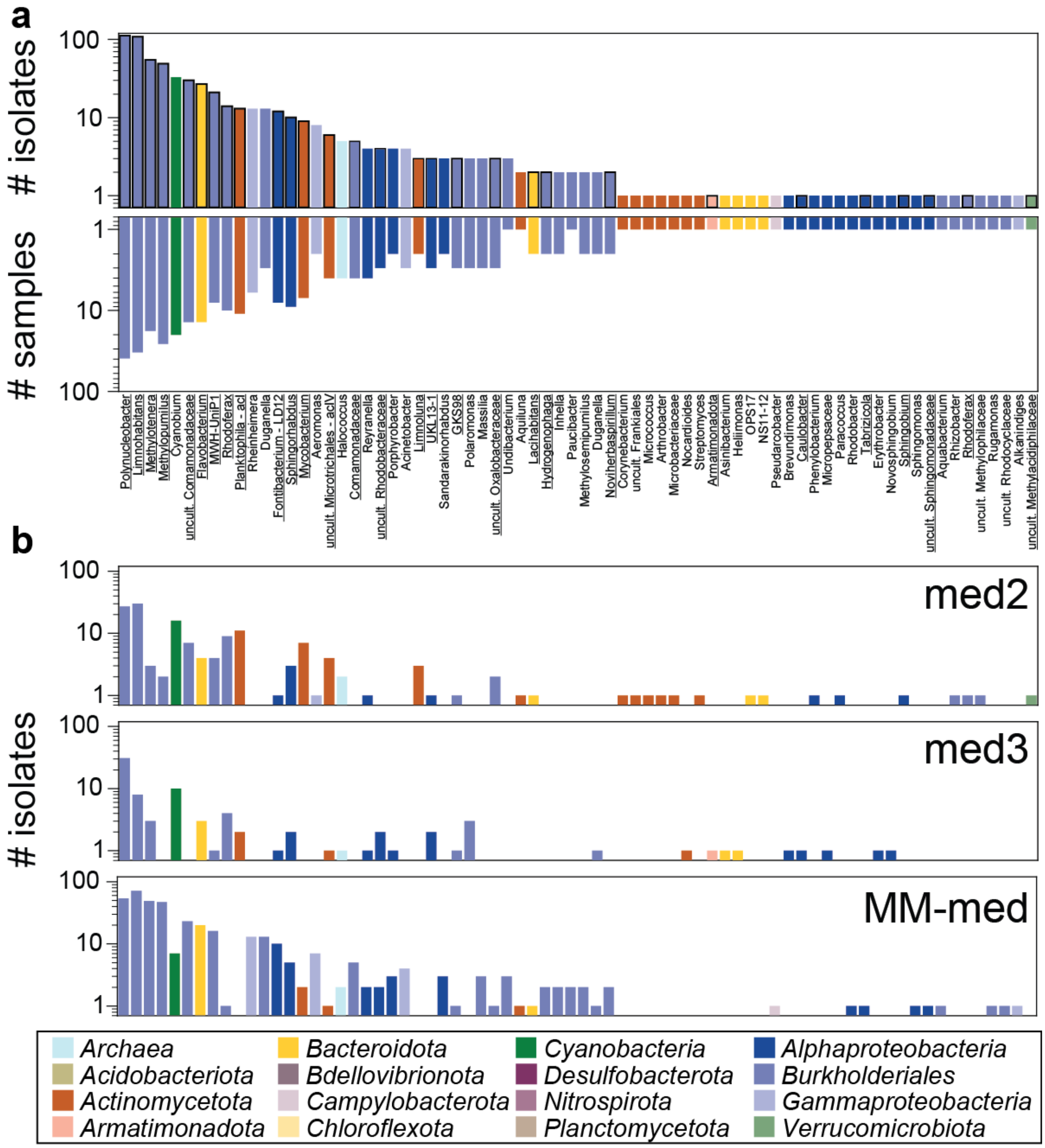

Supplementary Figure 3. Results of dilution-to extinction cultivation experiments. a Number of axenic strains sorted from high to low and number of samples where cultures were obtained from. Underlined taxa were subjected to whole-genome sequencing. Strains are colour-coded by taxonomy. **b** Taxa isolated in different media. med2, med3: media containing low concentrations of diverse carbon sources, MM-med: C1-medium containing methanol and methylamine as sole carbon source (see Supplementary Data 1 for media composition). Note log-scale of y-axes. Raw data is given in Supplementary Data 3.

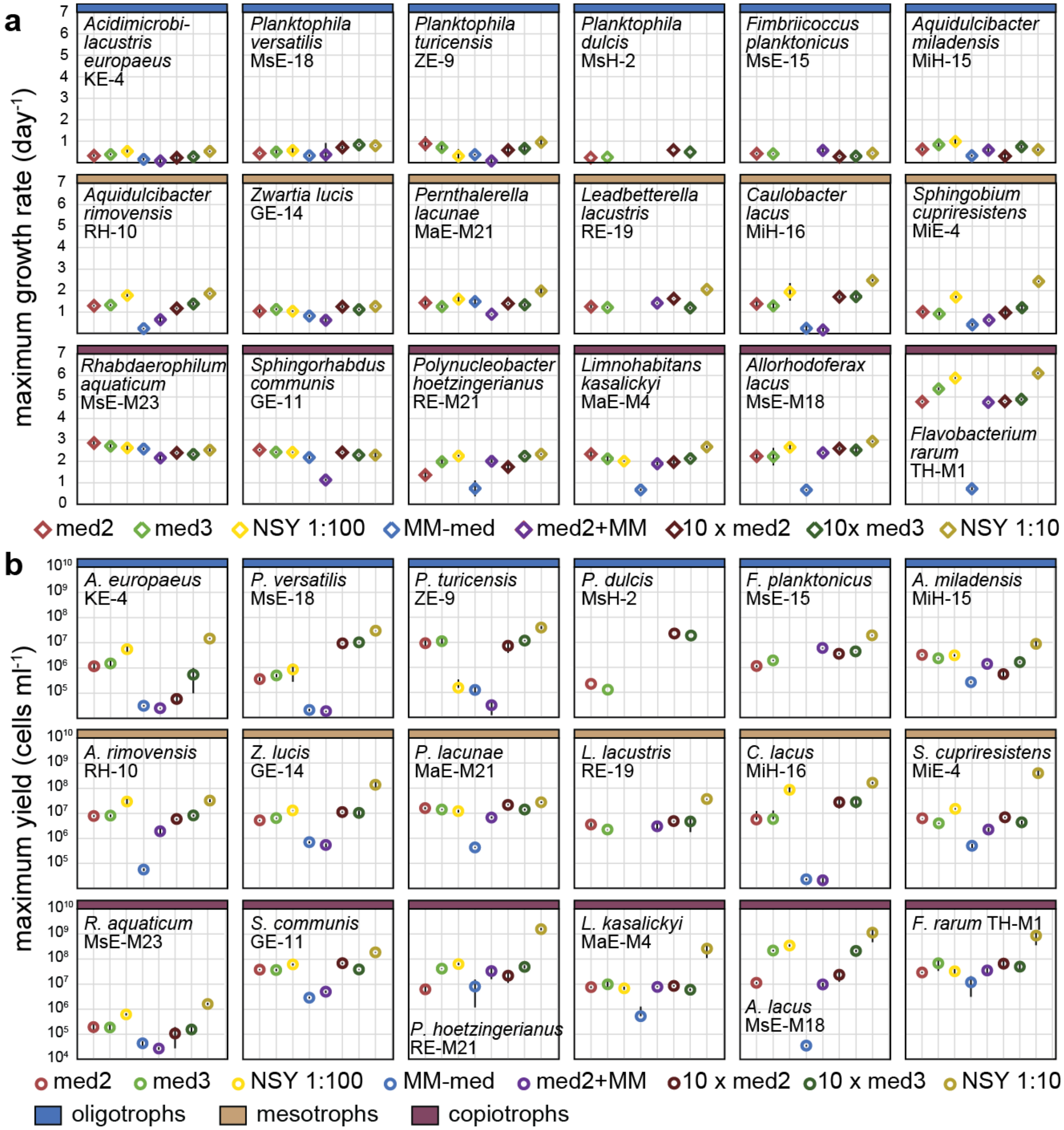

Supplementary Figure 4. Growth characteristics of selected cultures in up to eight different media. a Maximum growth rates. **b** Maximum cell yield, note the log-scale of y-axis. Shown are averages ± standard deviation of triplicates grown in up to eight different media. Grouping of strains in oligo-, meso- and copiotrophs is indicated by different colours (blue: oligotrophs; brown: mesotrophs; purple: copiotrophs). Raw data is given in Supplementary Data 5.

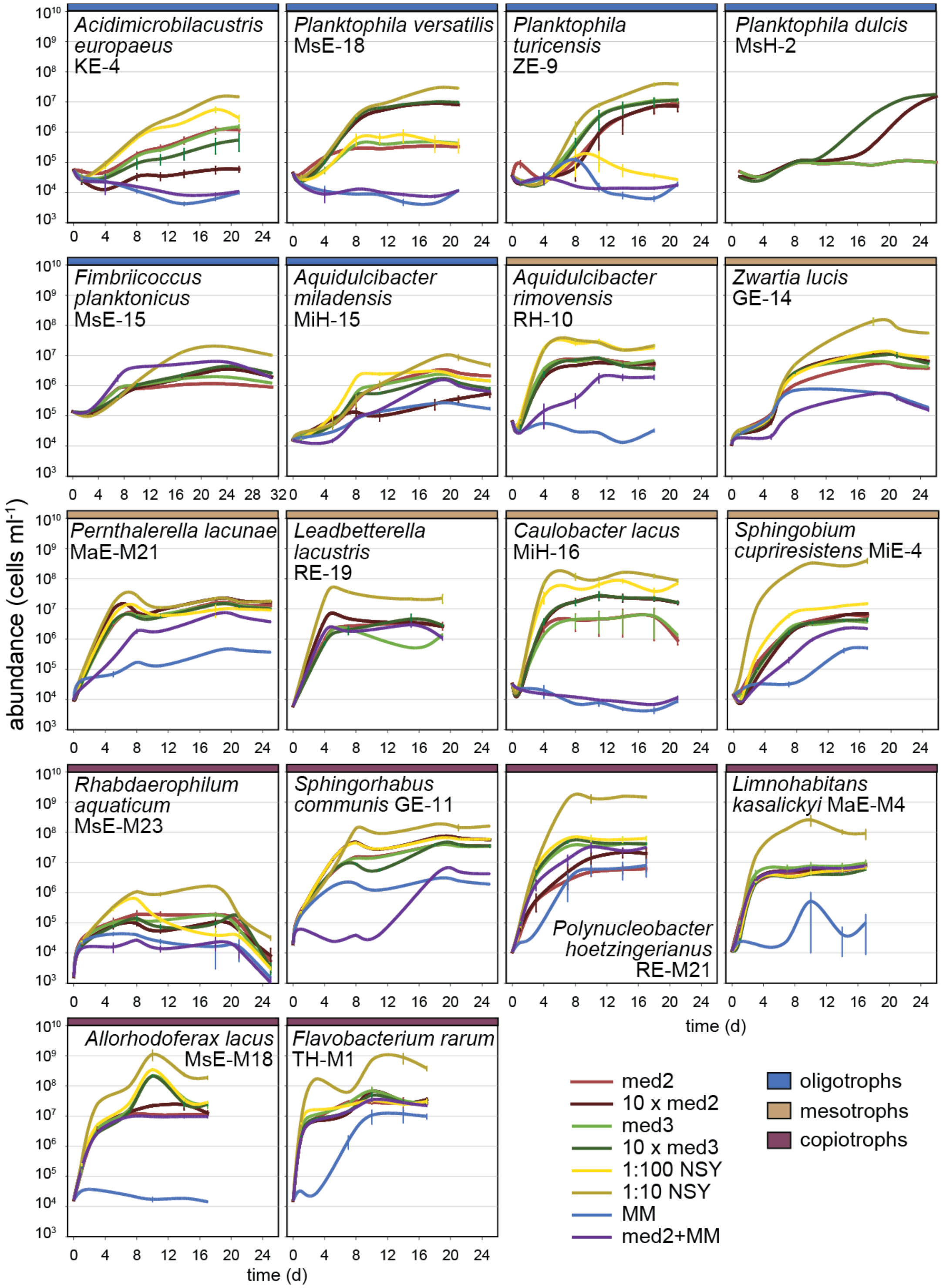

Supplementary Figure 5. Growth curves of selected cultures in up to eight different media. Shown are averages ± standard deviation of triplicates. Note log-scale of y-axis. Grouping of strains in oligo-, meso- and copiotrophs is indicated by different colours (blue: oligotrophs; brown: mesotrophs; purple: copiotrophs). Raw data is given in Supplementary Data 5.

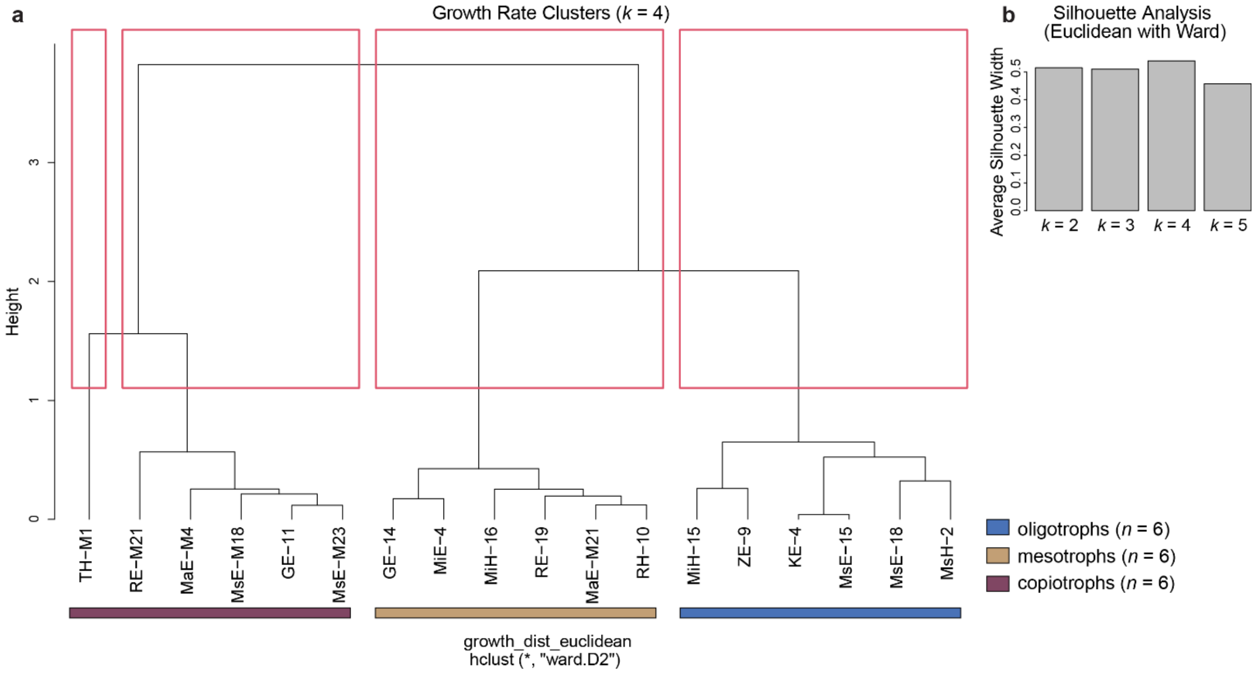

Supplementary Figure 6. Hierarchical clustering of maximum growth rates of different strains. a Hierarchical clustering dendrogram based on maximum growth rates of different strains. Clustering was performed using Euclidean distance and Ward's minimum variance linkage method (ward.D2). Red rectangles delineate the clusters identified based on the silhouette analysis. Strains are colour-coded at the base of the dendrogram according to trophic classifications (blue: oligotrophs; brown: mesotrophs; purple: copiotrophs). **b** Bar plot showing the average silhouette width for clusters ($k = 2$ to 5) with $k = 4$ corresponding to the highest average width. Raw data can be found in Supplementary Data 5.

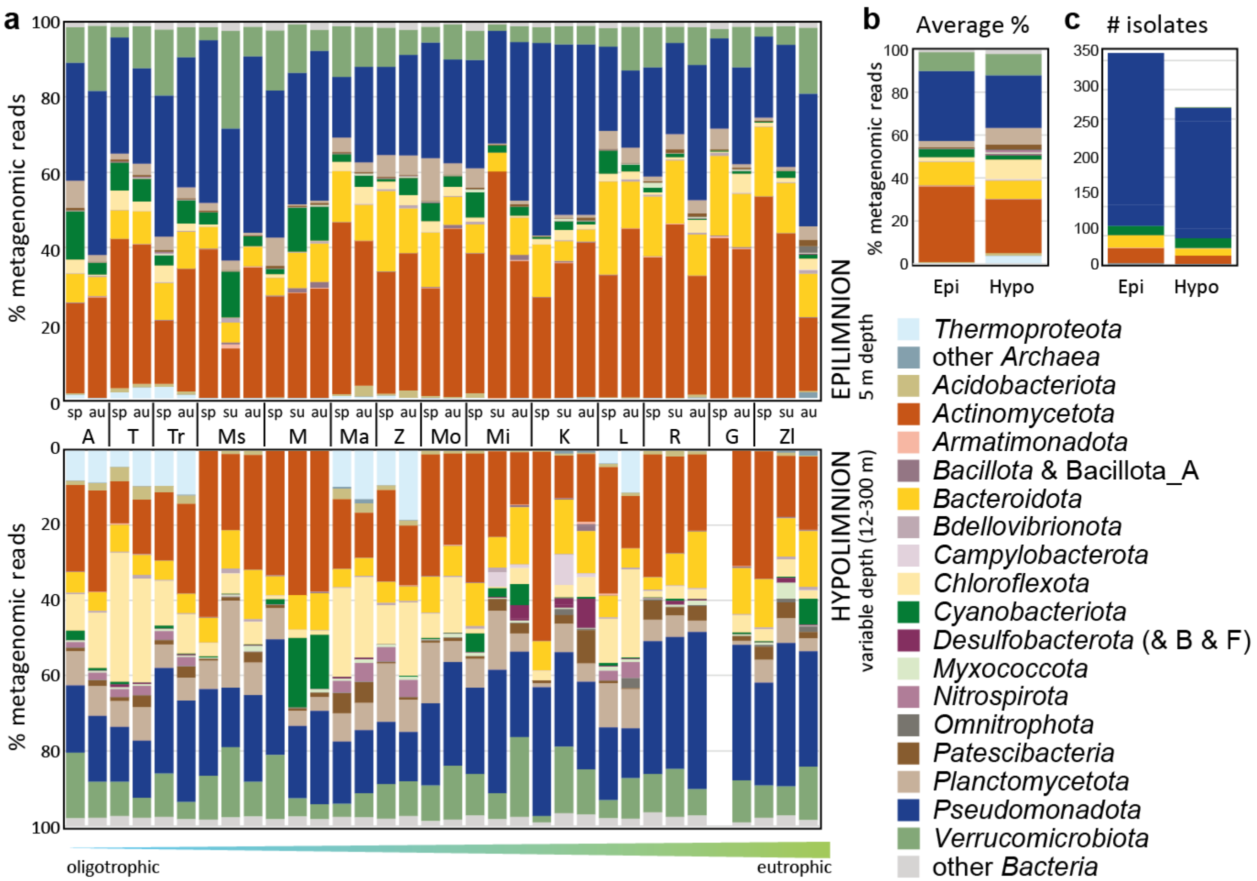

Supplementary Figure 7. Taxonomic classification of metagenomic reads using 59 single copy genes (SingleM). a Taxonomy of individual samples at the phylum level for the epi- and hypolimnion. Taxa with <1 % in any sample are summed up in others. Lakes are sorted from oligo- to eutrophic. Abbreviations: A: Attersee, T: Thun, Tr: Traunsee, Ms: Most, M: Medard, Ma: Maggiore, Z: Zurich, Mo: Mondsee, Mi: Milada, K: Klíčava, L: Lugano, R: Římov, G: Greifensee, Zl: Žlutice, sp: spring; su: summer; au: autumn. **b** Average contribution of individual phyla to epi- (Epi) and hypolimnetic (Hypo) samples. **c** Number of axenic strains gained from epi- and hypolimnetic samples. Raw data is given in Supplementary Data 6.

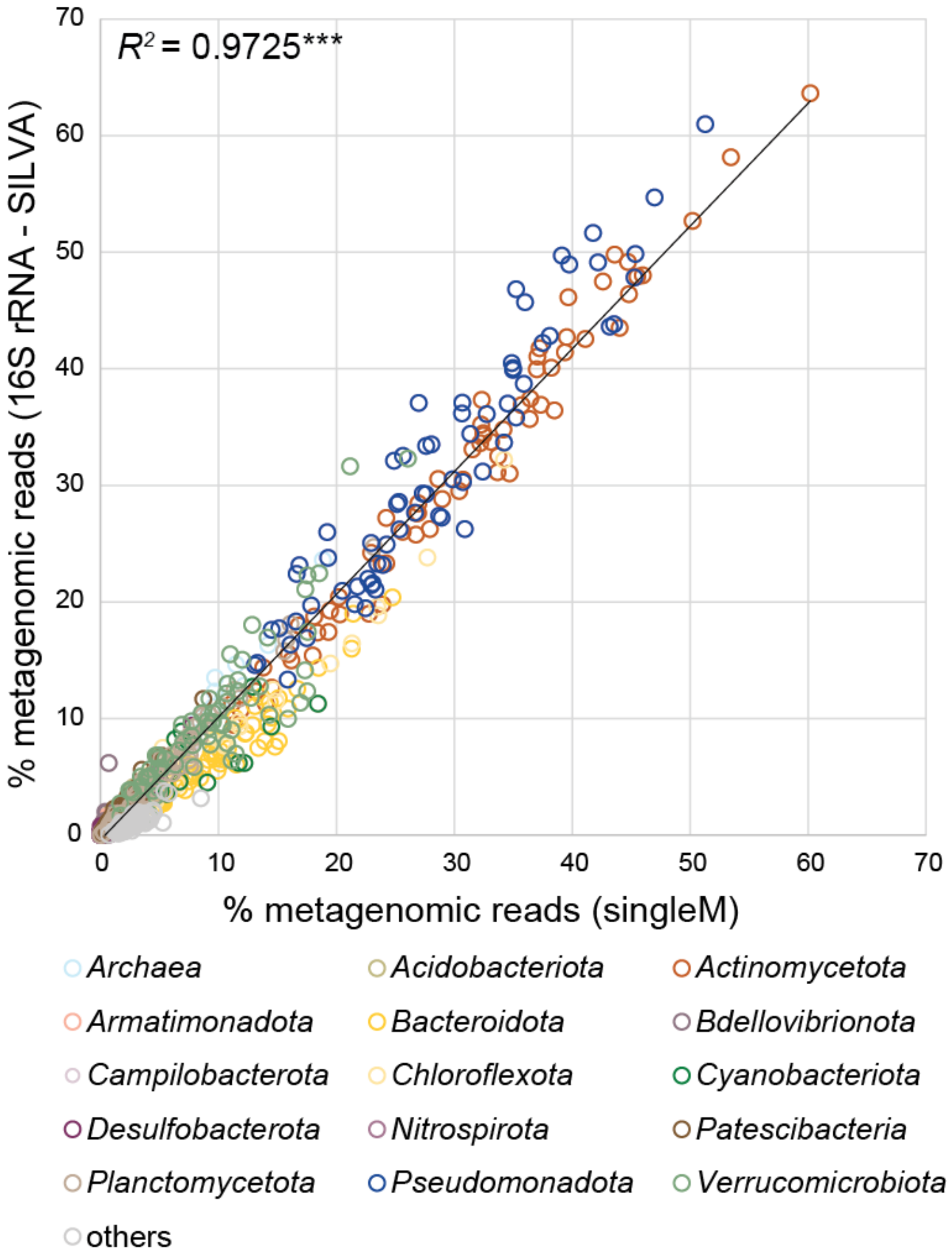

Supplementary Figure 8. Comparison of methods for taxonomic assessment of metagenomic reads *via* SingleM (59 marker genes) and copy number corrected SILVA classification of 16S rRNA genes. Raw data is given in Supplementary Data 6 and 8 and results of statistical tests can be found in Supplementary Data 4.

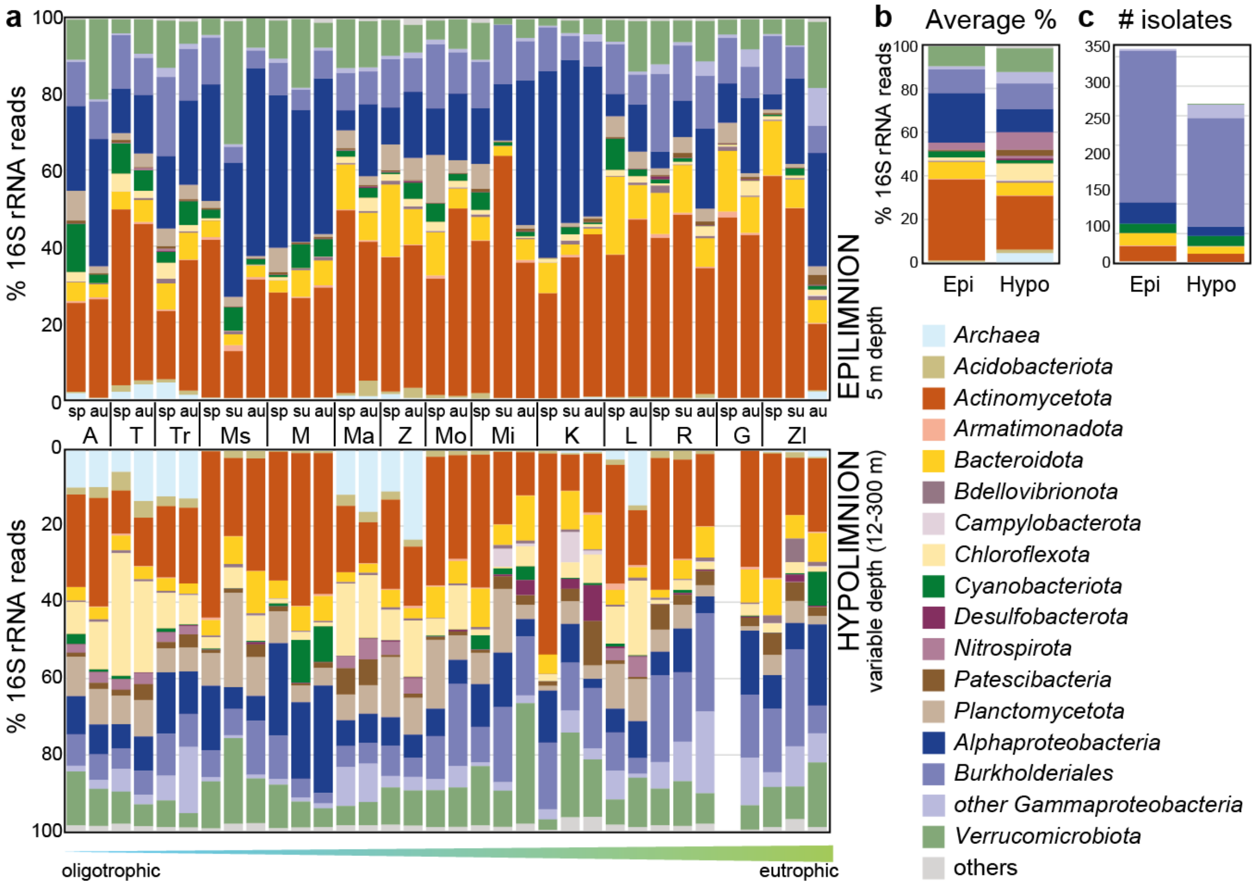

Supplementary Figure 9. Taxonomic classification of metagenomic reads using 16S rRNA genes and SILVA classification. a Taxonomy of individual samples at a phylum level for the epi- and hypolimnion. *Pseudomonadota* are shown at class level (*Alphaproteobacteria*, *Gammaproteobacteria*) with *Burkholderiales* shown separately. Taxa with <1 % in any sample are summed up in others. Lakes are sorted from oligo- to eutrophic. Abbreviations: A: Attersee, T: Thun, Tr: Traunsee, Ms: Most, M: Medard, Ma: Maggiore, Z: Zurich, Mo: Mondsee, Mi: Milada, K: Klíčava, L: Lugano, R: Římov, G: Greifensee, Zl: Žlutice, sp: spring; su: summer; au: autumn. **b** Average contribution of individual phyla to epi- (Epi) and hypolimnetic (Hypo) samples. **c** Number of axenic strains gained from epi- and hypolimnetic samples. Raw data is given in Supplementary Data 8.

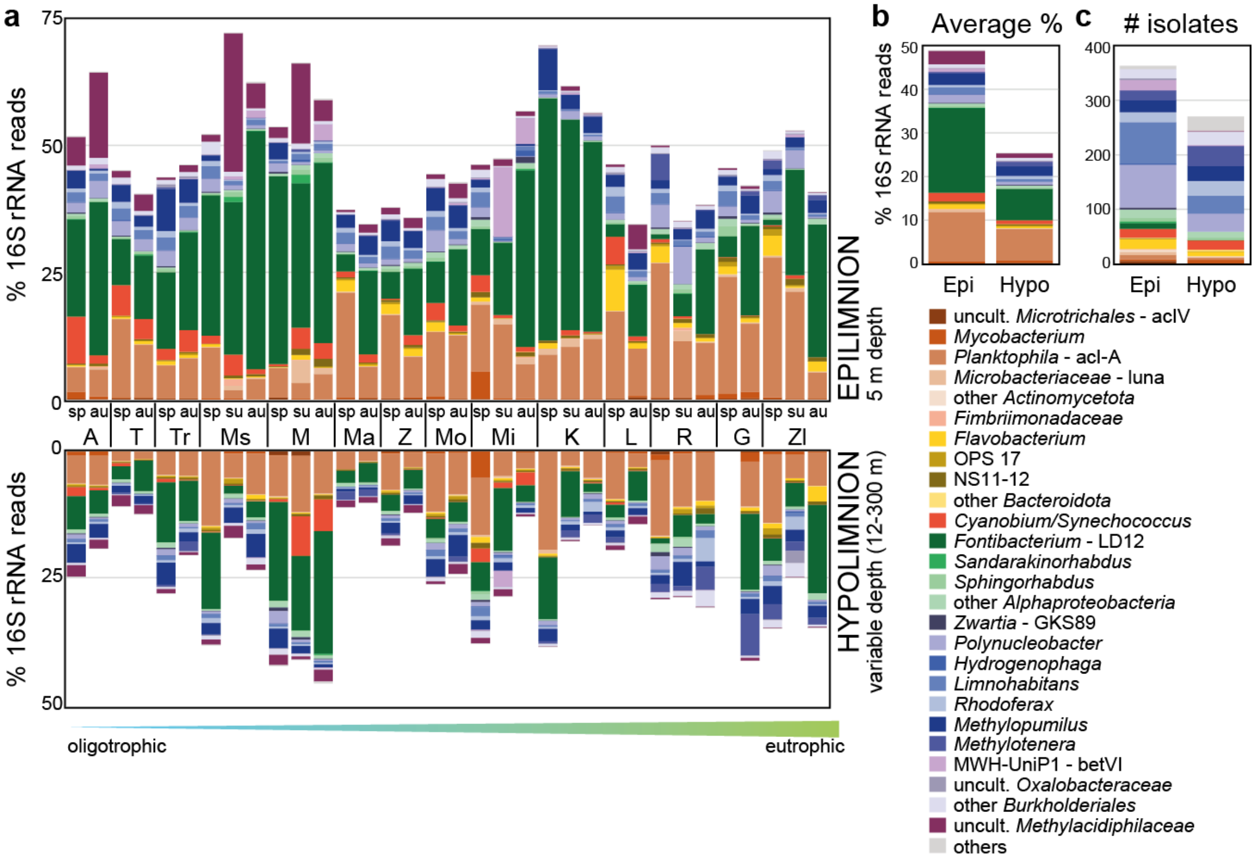

Supplementary Figure 10. Representation of the culture collection in the sampled lakes. a Summed up relative abundances of taxa with representatives in the culture collection in the epi- and hypolimnion of the sampled lakes. Metagenomic 16S rRNA reads were taxonomy-assigned with SILVA SSU database RefNR99 138.1. Lakes are sorted from oligo- to eutrophic. Abbreviations: A: Attersee, T: Thun, Tr: Traunsee, Ms: Most, M: Medard, Ma: Maggiore, Z: Zurich, Mo: Mondsee, Mi: Milada, K: Klíčava, L: Lugano, R: Římov, G: Greifensee, Zl: Žlutice, sp: spring; su: summer; au: autumn. **b** Average contribution of cultivated taxa to epi- (Epi) and hypolimnetic (Hypo) samples. **c** Number of strains isolated from the epi- and hypolimnion. Raw data is given in Supplementary Data 9.

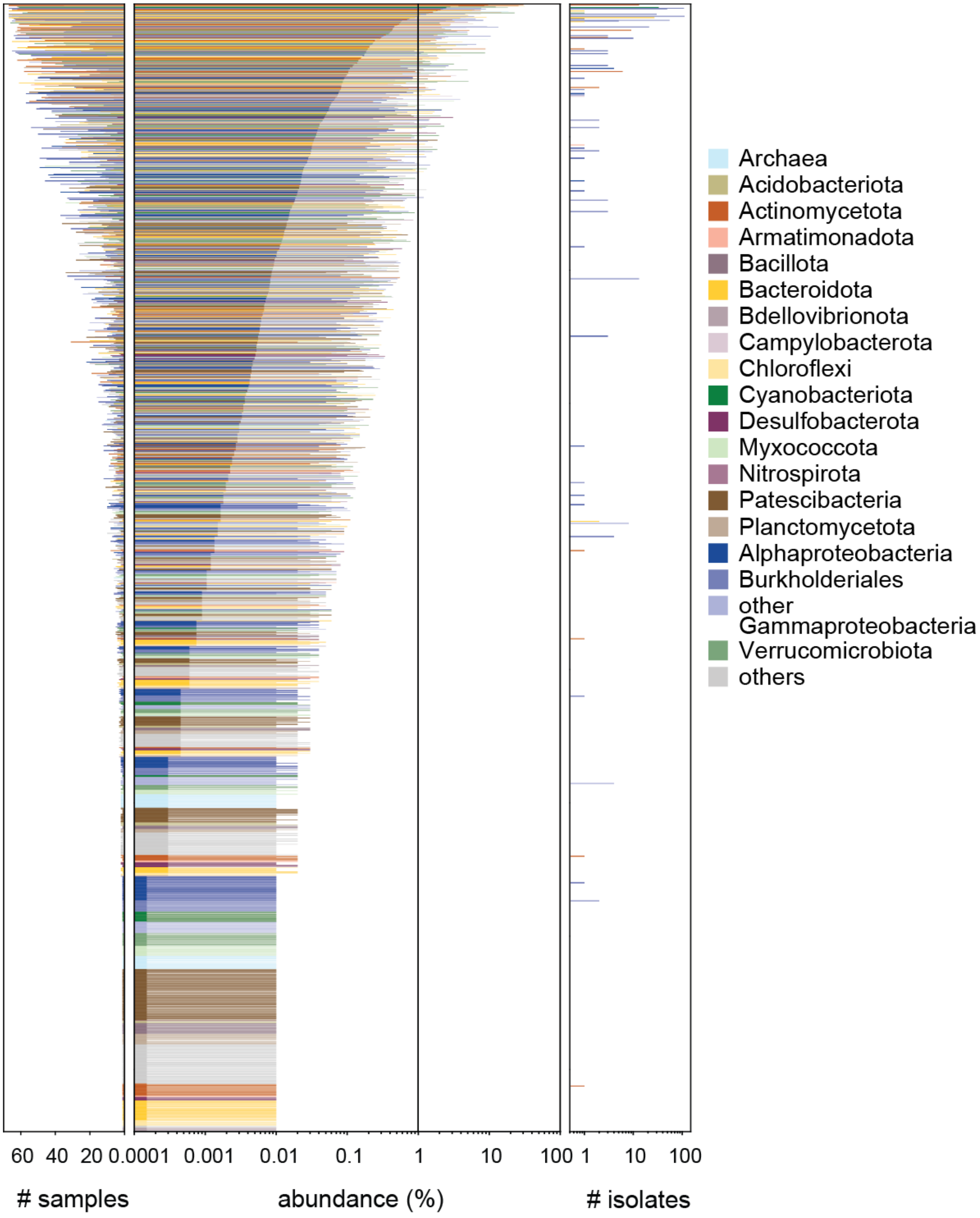

Supplementary Figure 11. Prevalence (number of samples), rank-abundance curve (% abundance), and number of axenic strains of all 1,734 genera present in any sample. Averages are shown as thick lines, maxima as thin lines**.** Metagenomic reads were taxonomy-assigned with SingleM. A figure of the most abundant 157 genera can be found as Fig. 3A. Raw data is given in Supplementary Data 7.

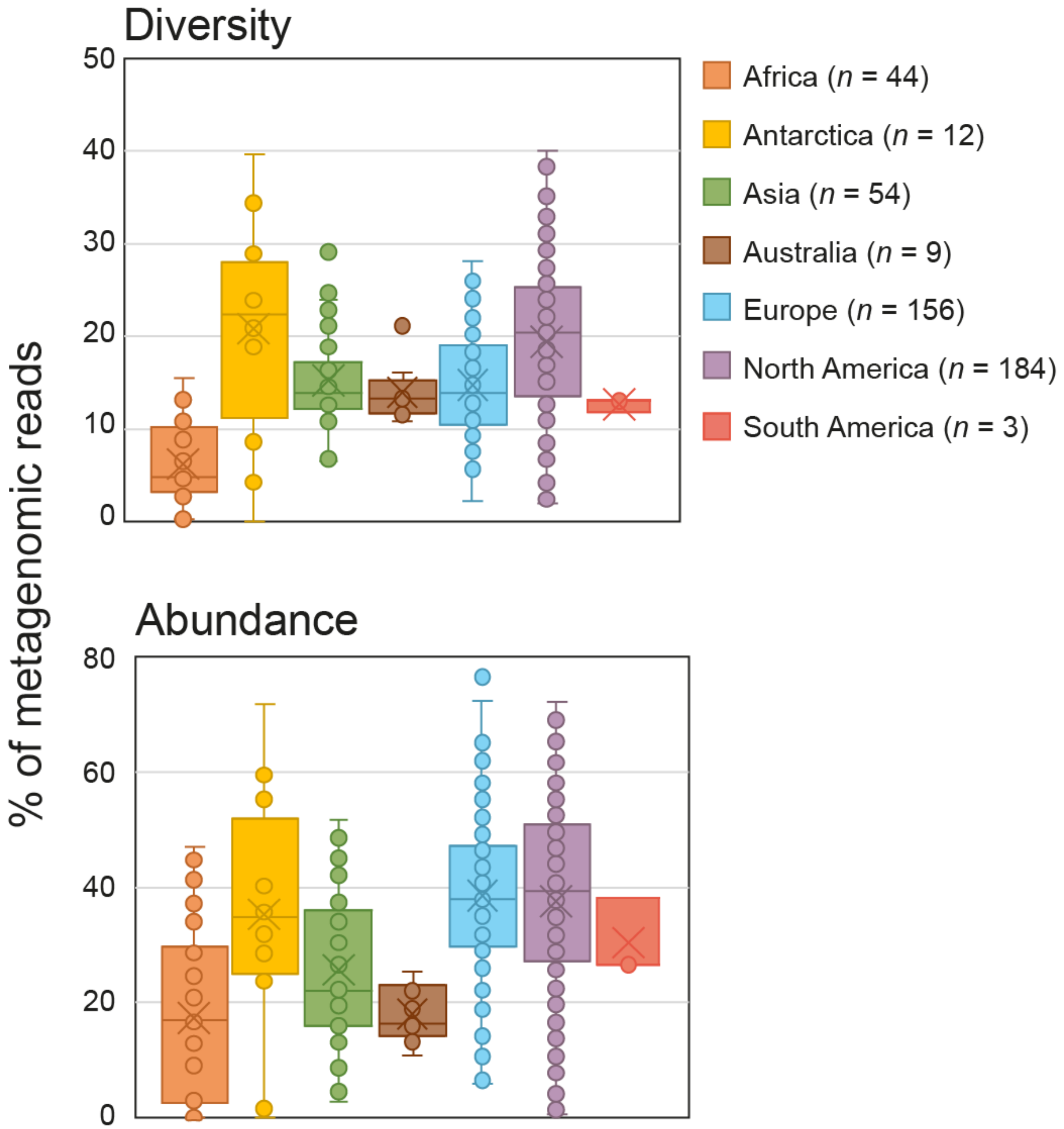

Supplementary Figure 12. Representation of the culture collection in 462 freshwater metagenomic samples from seven continents. Proportion of genera from the culture collection relative to the total number of genera (diversity) and their summed up relative abundances (abundance) in in 462 publicly available metagenomes from seven continents. Metagenomic reads were taxonomy-assigned with SingleM, raw data is given in Supplementary Data 10. Boxes indicate the 25th and 75th quantiles, medians are displayed by central lines, whiskers indicate the 5th and 95th quantiles. For reasons of simplicity, significant differences between different continents are not shown in the figure, results of statistical tests can be found in Supplementary Data 4. Raw data is given in Supplementary Data 10.

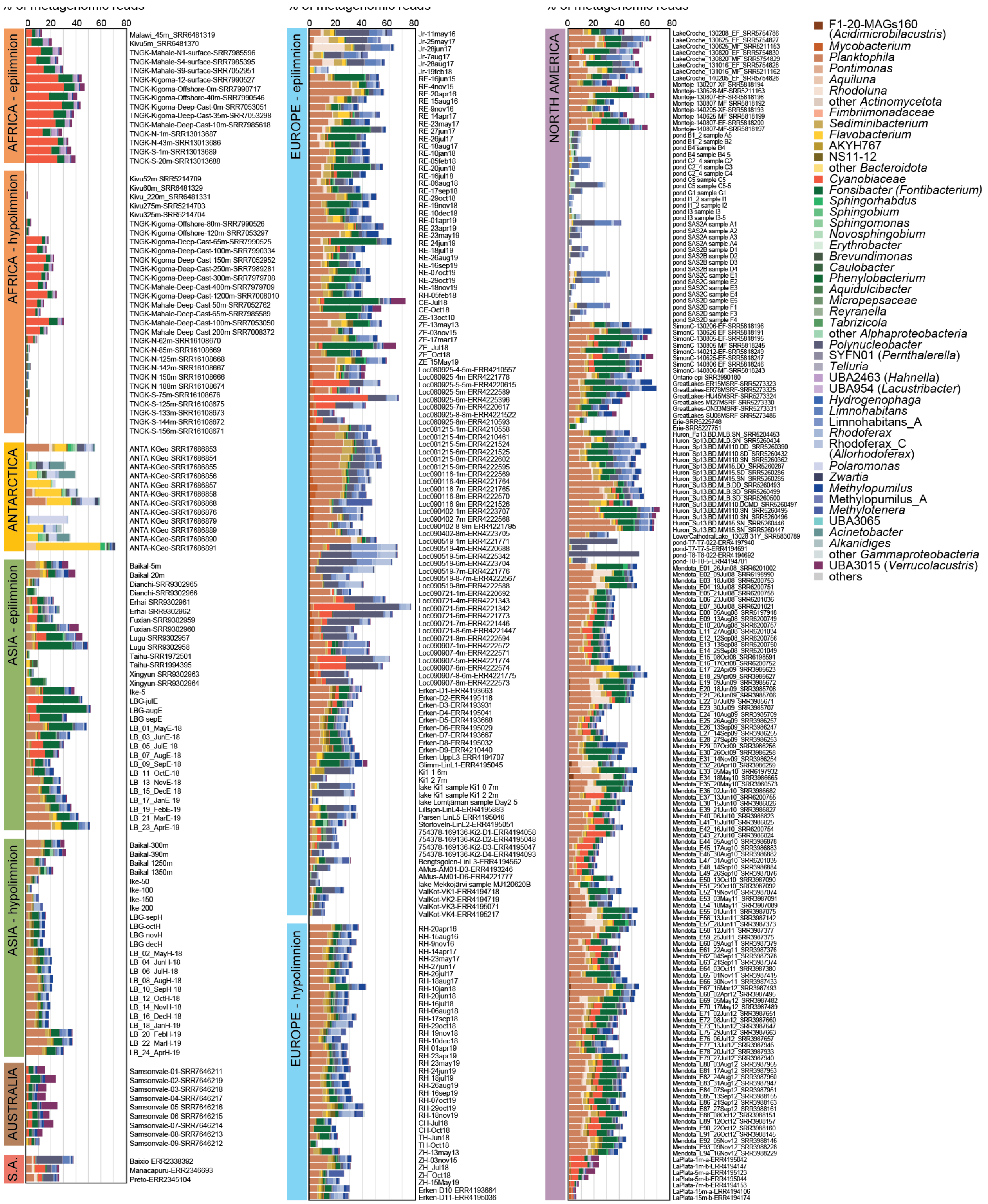

Supplementary Figure 13. Global occurrence of genera from our culture collection. Summed up relative abundances of taxa with representatives in the culture collection in the epi- and hypolimnion of freshwater metagenomic samples from seven continents (n = 462). Continents are colour-coded as in Supplementary Figure 12. Metagenomic reads were taxonomy-assigned with SingleM, raw data is given in Supplementary Data 10. S.A.: South America.

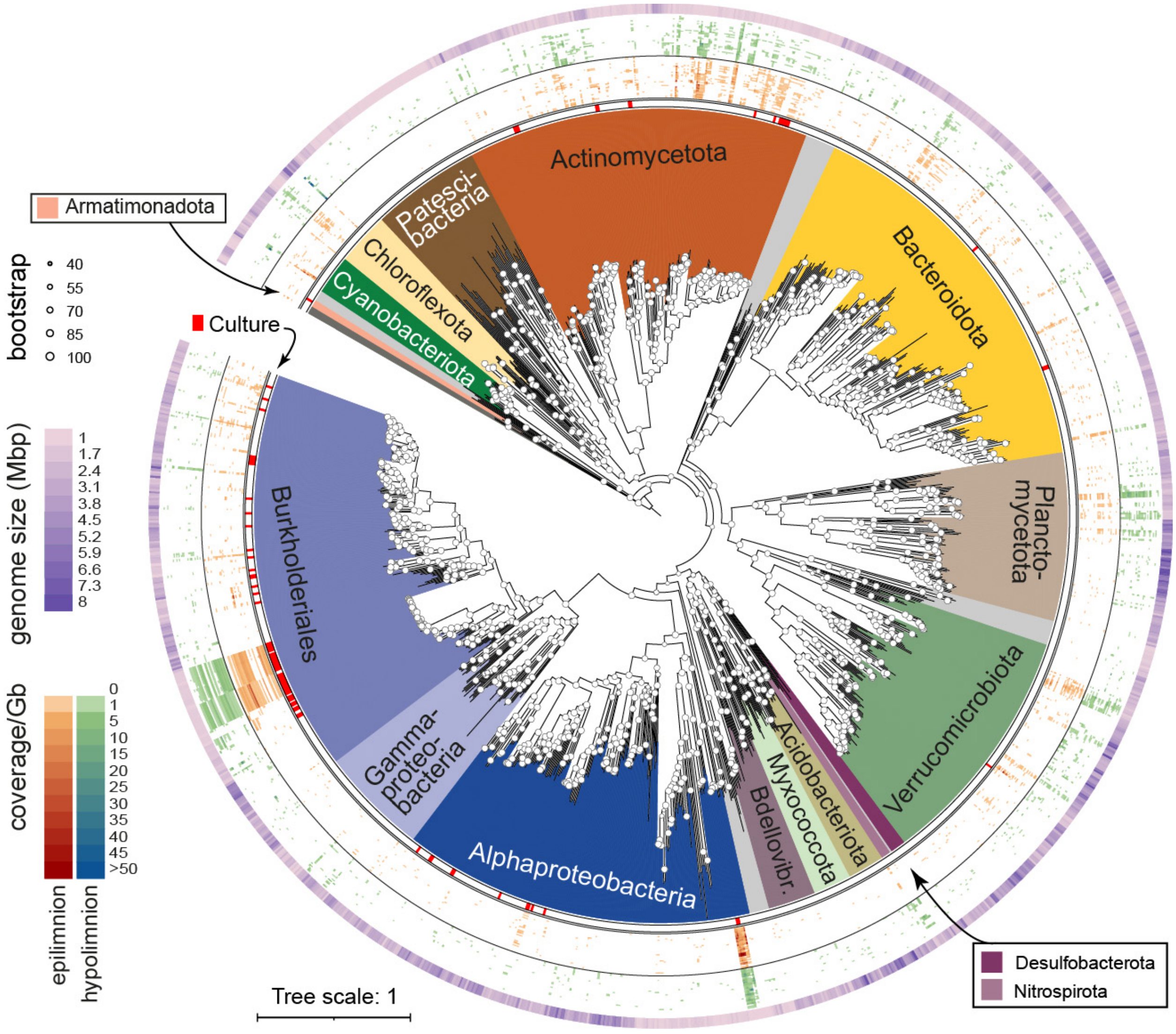

Supplementary Figure 14. Phylogenomic tree of the culture collection and MAGs assembled from the same water samples. The tree is based on 120 single copy marker protein sequences of culture genomes marked in red (n = 87) and dereplicated MAGs (95% ANI) assembled from the same water samples (n = 1,294). Metagenomic fragment recruitment (coverage/Gb) of individual genomes in the 67 metagenomes is separately shown for epi- and hypolimnetic samples in shades of orange-red and green-blue, respectively. Genome size is shown in shades of purple. Raw data is given in Supplementary Data 16.

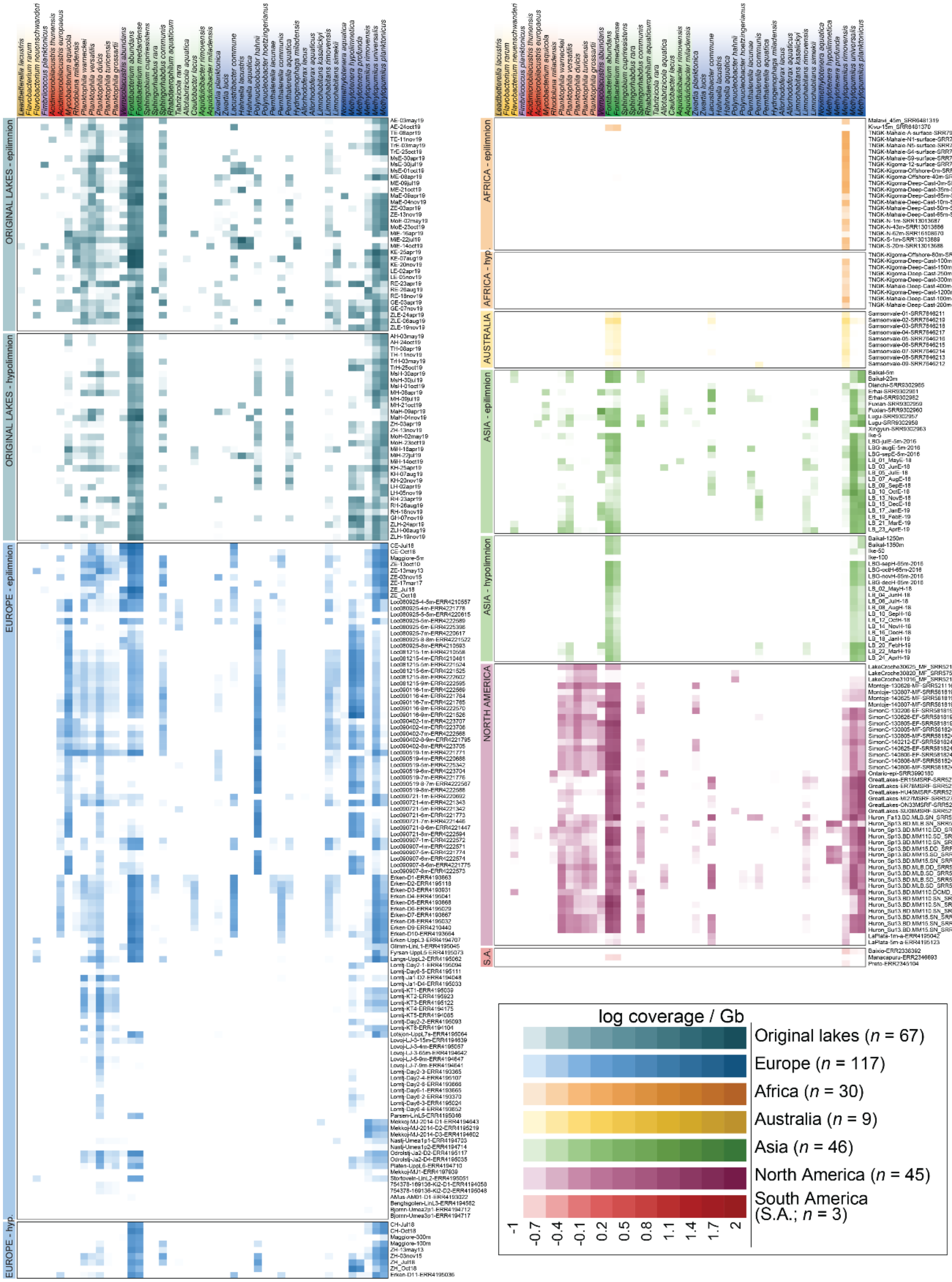

Supplementary Figure 15. Metagenomic fragment recruitment of the culture collection. Recruitment values (log coverage per Gb) are shown for the same water samples from which cultures were obtained (original lakes; $n = 67$) and publicly available datasets ($n = 250$) separately for each continent. Genomes were dereplicated on species level (95% ANI) and colour-coded as in Fig. 5. Raw data is given in Supplementary Data 16 and 17.

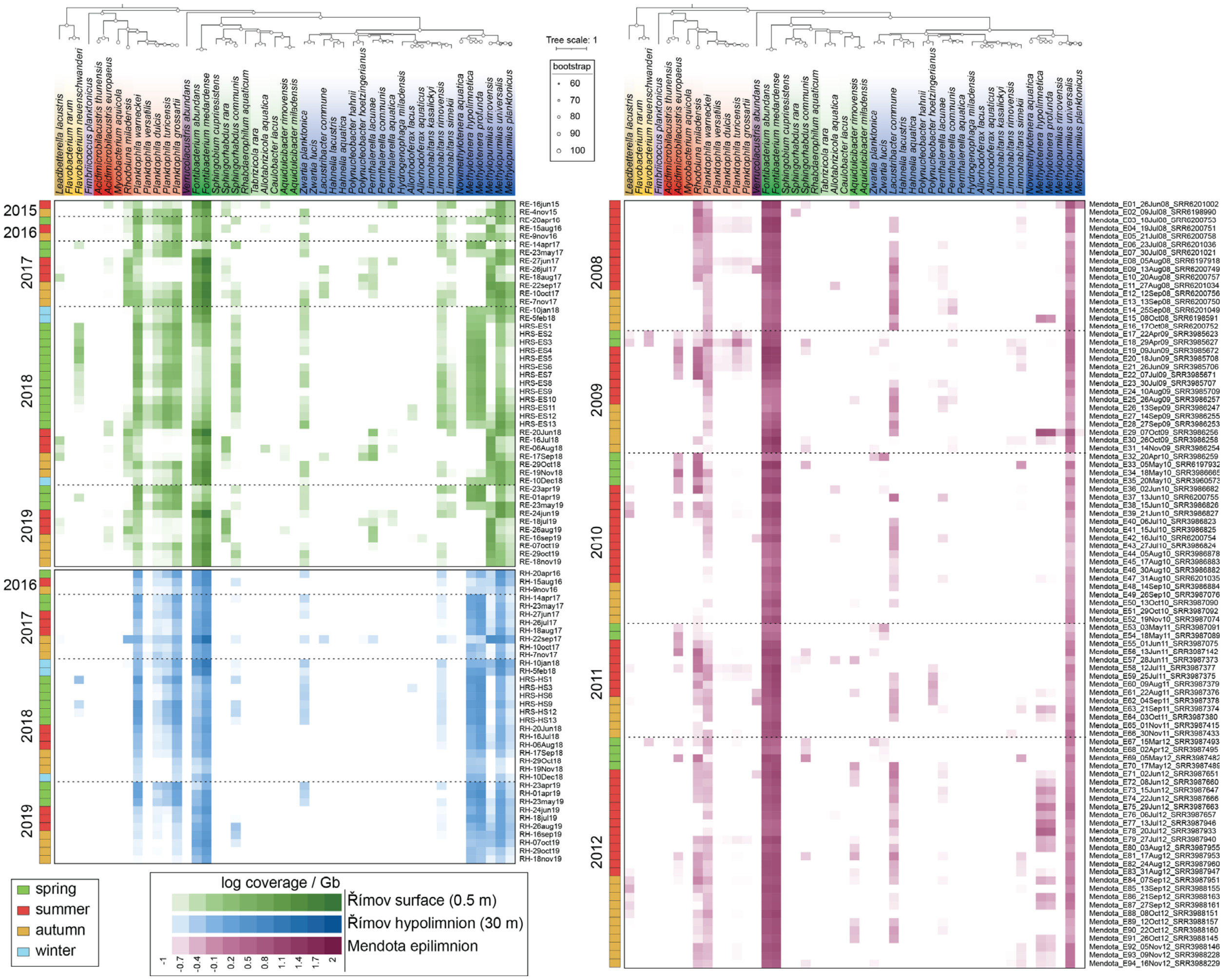

Supplementary Figure 16. Metagenomic fragment recruitment of seasonally resolved time-series samples. Left side: The Římov reservoir (CZ, 2015-2019; *n* = 81), samples were gained from the surface (0.5 m depth; shades of green) and hypolimnion (30 m depth; shades of blue). Right side: Lake Mendota (USA, 2008-2012, *n* = 94), samples were gained from the epilimnion (integrated water samples; shades of purple). Genomes were dereplicated on species level (95% ANI) and colour-coded as in Fig. 5. Different seasons are colour-coded (spring: green; summer: red; autumn: brown; winter: blue). Raw data is given in Supplementary Data 17.

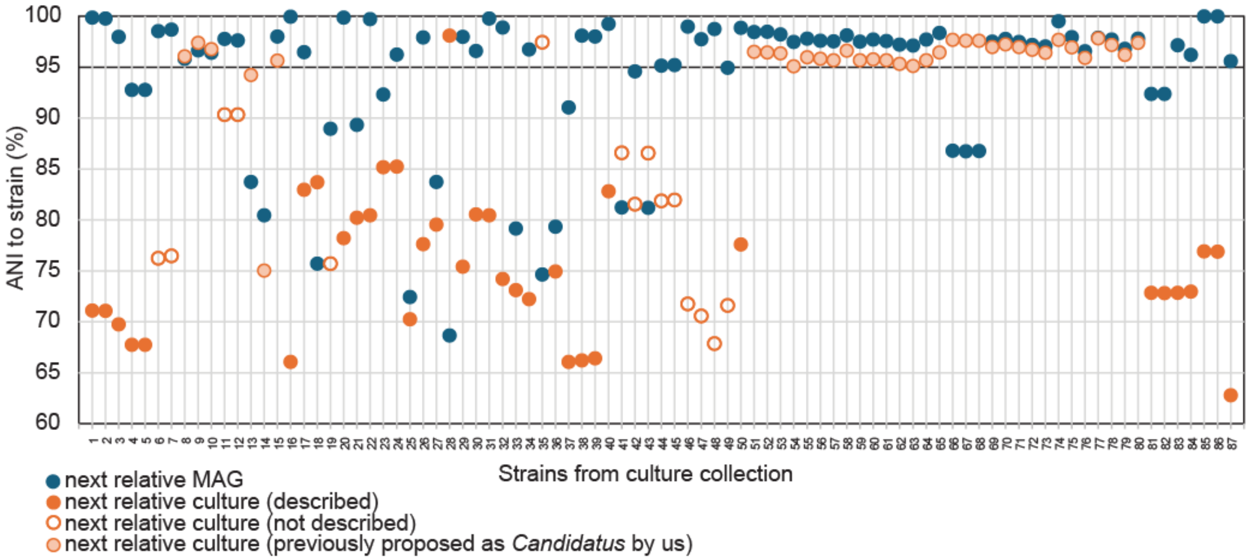

Strain code:

1 *Acidimicrobilacustris thunensis* TE-4
2 *Acidimicrobilacustris thunensis* TE-7
3 *Acidimicrobilacustris europaeus* KE-4
4 *Rhodoluna miladensis* MiE-23b
5 *Rhodoluna miladensis* MiE-24b
6 *Mycobacterium aquicola* MaE-M6b
7 *Mycobacterium aquicola* MiE-22
8 *Planktophila dulcis* MaH-2
9 *Planktophila dulcis* MsH-2
10 *Planktophila dulcis* TrE-23
11 *Planktophila grossartii* RE-3
12 *Planktophila grossartii* RH-3
13 *Planktophila turicensis* ZE-9
14 *Planktophila warneckei* RE-8
15 *Planktophila versatilis* MsE-18
16 *Fimbriicoccus planktonicus* MsE-15
17 *Leadbetterella lacustris* RE-19
18 *Flavobacterium rarum* TH-M1
19 *Flavobacterium neuenschwanderi* GE-10
20 *Caulobacter lacus* MiH-16
21 *Aquidulcibacter rimovensis* RH-10
22 *Aquidulcibacter miladensis* MiH-15
23 *Fontibacterium abundans* MiE-29
24 *Fontibacterium medardense* ME-17
25 *Rhabdaerophilum aquaticum* MsE-M23
26 *Tabrizicola rara* LH-M10
27 *Allotabrizicola aquatica* RE-M30
28 *Sphingobium cupriresistens* MiE-4
29 *Sphingorhabdus rara* RE-M21a
30 *Sphingorhabdus communis* GE-11
31 *Sphingorhabdus communis* ZE-10
32 *Hydrogenophaga miladensis* MiE-M28
33 *Limnohabitans simekii* MiE-M12
34 *Limnohabitans rimovensis* RE-1
35 *Limnohabitans kasalickyi* MaE-M4
36 *Polynucleobacter hoetzingerianus* RE-M21
37 *Pernthalerella aquatica* GE-M3
38 *Pernthalerella lacunae* MaE-M21
39 *Pernthalerella communis* MsE-6
40 *Polynucleobacter hahnii* ZE-4
41 *Allorhodoferax aquaticus* MiE-M13
42 *Allorhodoferax lacus* MsE-M18
43 *Allorhodoferax aquaticus* MsE-M22
44 *Allorhodoferax lacus* MsH-M24
45 *Allorhodoferax lacus* ZE-M1
46 *Hahnella aquatica* MiE-11
47 *Hahnella lacustris* MsE-M47
48 *Lacustribacter communis* MsE-M52
49 *Zwartia planktonica* RE-10
50 *Zwartia lucis* GE-14
51 *Methylopumilus planktonicus* KE-4b
52 *Methylopumilus planktonicus* LE-M7
53 *Methylopumilus planktonicus* MaE-M16
54 *Methylopumilus planktonicus* MaE-M22
55 *Methylopumilus planktonicus* MoH-M15
56 *Methylopumilus planktonicus* MoH-M17
57 *Methylopumilus planktonicus* MoH-M19
58 *Methylopumilus planktonicus* MoH-M36
59 *Methylopumilus planktonicus* MoH-M8
60 *Methylopumilus planktonicus* MsH-M18
61 *Methylopumilus planktonicus* MsH-M39
62 *Methylopumilus planktonicus* MsH-M42
63 *Methylopumilus planktonicus* TH-M4
64 *Methylopumilus planktonicus* ZE-M7
65 *Methylopumilus planktonicus* ZE-M8
66 *Methylopumilus rimovensis* RE-M17
67 *Methylopumilus rimovensis* RE-M20
68 *Methylopumilus rimovensis* RE-M24
69 *Methylopumilus universalis* GE-M14
70 *Methylopumilus universalis* GH-M24
71 *Methylopumilus universalis* GH-M4
72 *Methylopumilus universalis* LE-M21
73 *Methylopumilus universalis* MaE-M17
74 *Methylopumilus universalis* MH-M4
75 *Methylopumilus universalis* MH-M5
76 *Methylopumilus universalis* MiE-M1
77 *Methylopumilus universalis* RE-M9
78 *Methylopumilus universalis* RH-M37
79 *Methylopumilus universalis* ZE-M6
80 *Methylopumilus universalis* ZIE-M21
81 *Methylotenera profunda* RE-M3
82 *Methylotenera profunda* RH-M32
83 *Methylotenera hypolimnetica* RH-M31
84 *Methylotenera hypolimnetica* ZIE-M10
85 *Novimethylotenera aquatica* ME-M6
86 *Novimethylotenera aquatica* MsE-M29
87 *Verrucolacustris abundans* MiH-22

Supplementary Figure 17. Average nucleotide identities (ANI) between strains from our culture collection and closely related MAGs and previously cultivated species. The species border (95% ANI) is indicated with a solid line. Raw data is given in Supplementary Data 14.

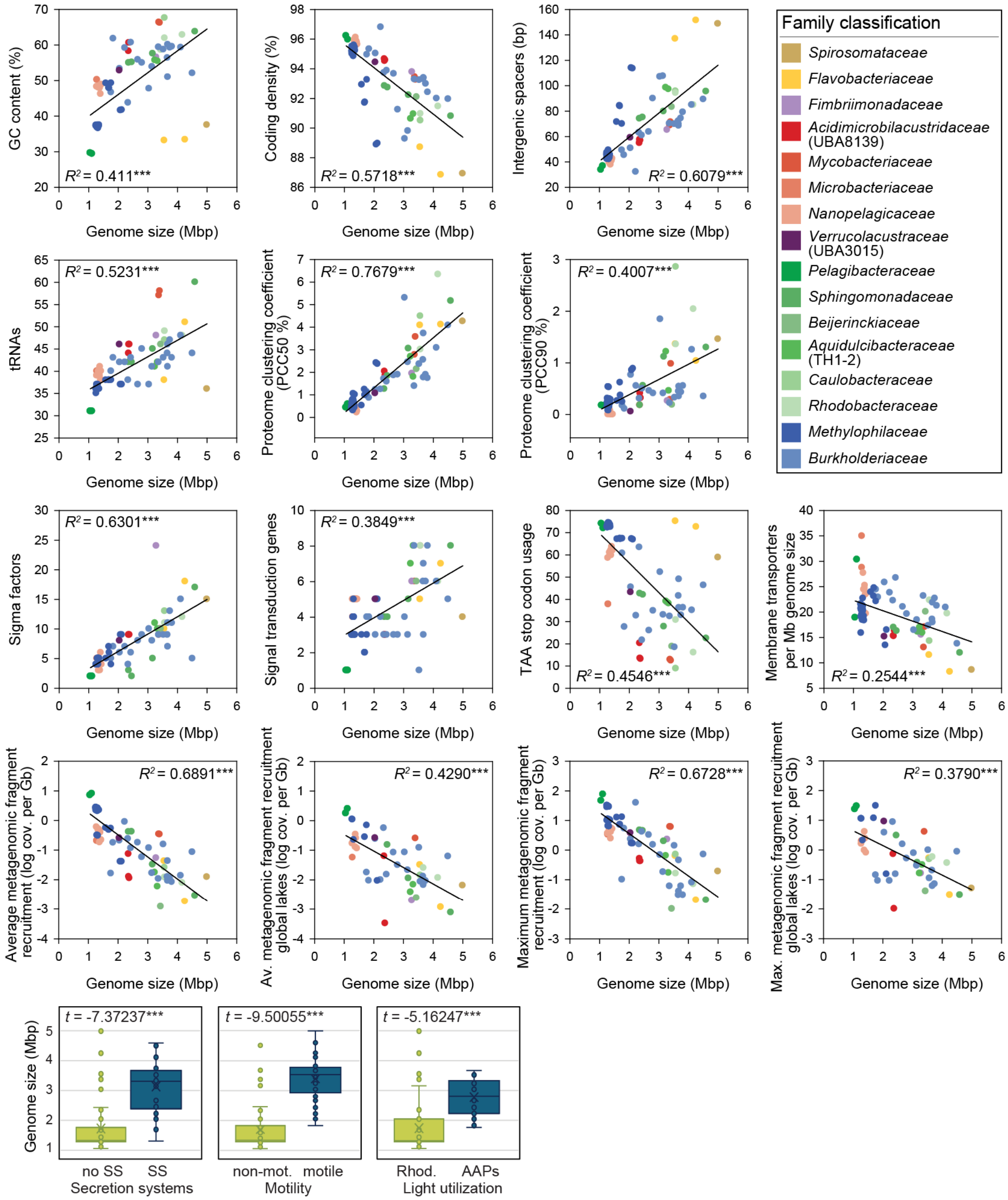

Supplementary Figure 18. Relationship between genome size and various genomic features, metabolic pathways and abundances in natural samples. Boxes indicate the 25th and 75th quantiles, medians are displayed by central lines, whiskers indicate the 5th and 95th quantiles, individual samples are shown as circles.Av. recruitment: Average metagenomic fragment recruitment in original samples ($n = 67$). Av. recruitment global: Average metagenomic fragment recruitment in metagenomes from six continents ($n = 425$). Max. recruitment: Maximum metagenomic fragment recruitment values in any of the samples. SS: Secretion systems type II, IV, or VI. Non-mot.: non-motile. Rhod: rhodopsin-containing strains. AAPs: anoxygenic aerobic phototrophs. ***: $p < 0.0001$ for either linear regression (upper four rows) or t-tests (lowest row). Results of statistical tests can be found in Supplementary Data 4.

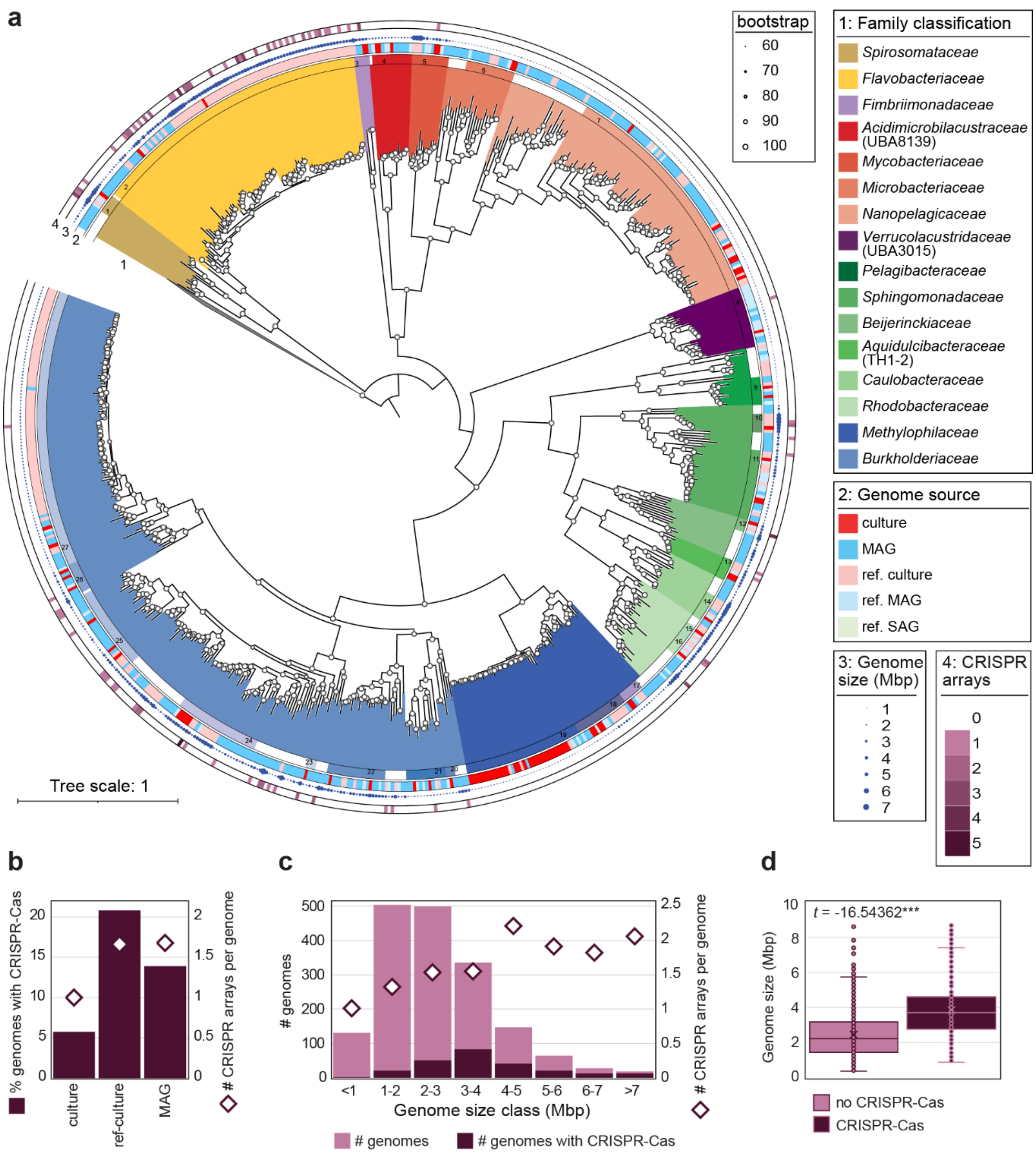

Supplementary Figure 19. Occurrence of CRISPR-Cas arrays in cultures, MAGs and closely related cultivates references. a The same phylogenomic tree as in Fig. 4 including the number of CRISPR-Cas arrays per genome (shades of purple). **b** Percentage of genomes with CRISPR-Cas arrays (purple bars) and number of CRISPR-Cas arrays per genome (purple diamonds) separately shown for cultures, reference cultures, and MAGs or, **c** different genome size classes. **d** Genome sizes of microbes with or without CRISPR-Cas arrays. Boxes indicate the 25th and 75th quantiles, medians are displayed by central lines, whiskers indicate the 5th and 95th quantiles, individual samples are shown as circles. ***: $p < 0.0001$ for significant differences in genome sizes (t-test), results of statistical tests can be found in Supplementary Data 4.

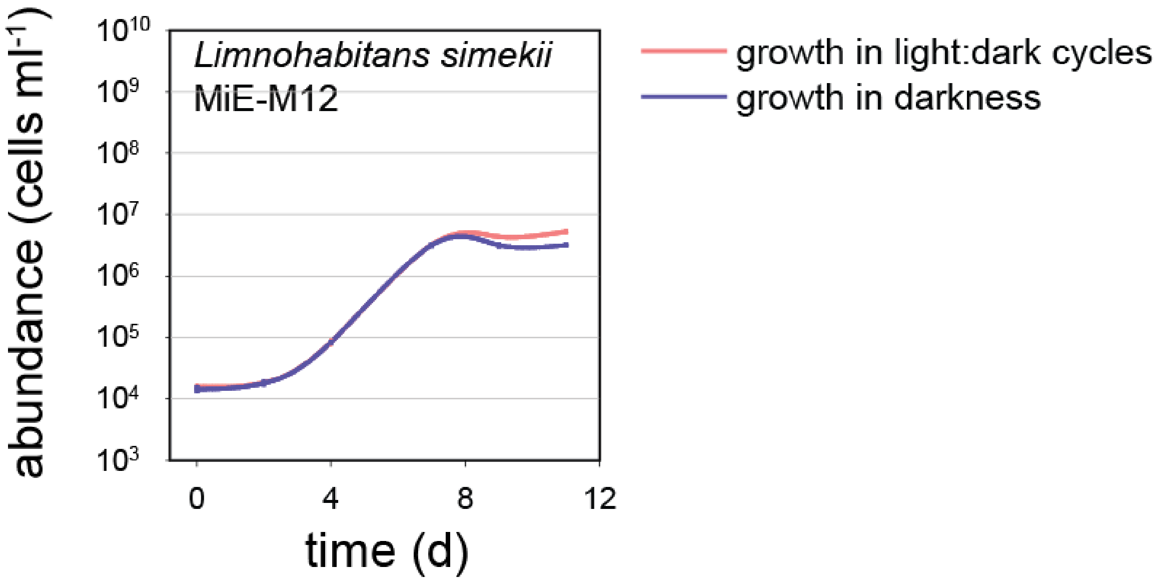

Supplementary Figure 20. Potential photoautotrophy of one strain. Growth curves of *Limnohabitans simekii* MiE-M12 in a medium without carbon sources except for vitamins grown in light:dark conditions (12:12 h; red line) and in darkness (blue line). Shown are means of three replicates +/- standard deviation. Results of statistical tests can be found in Supplementary Data 4.

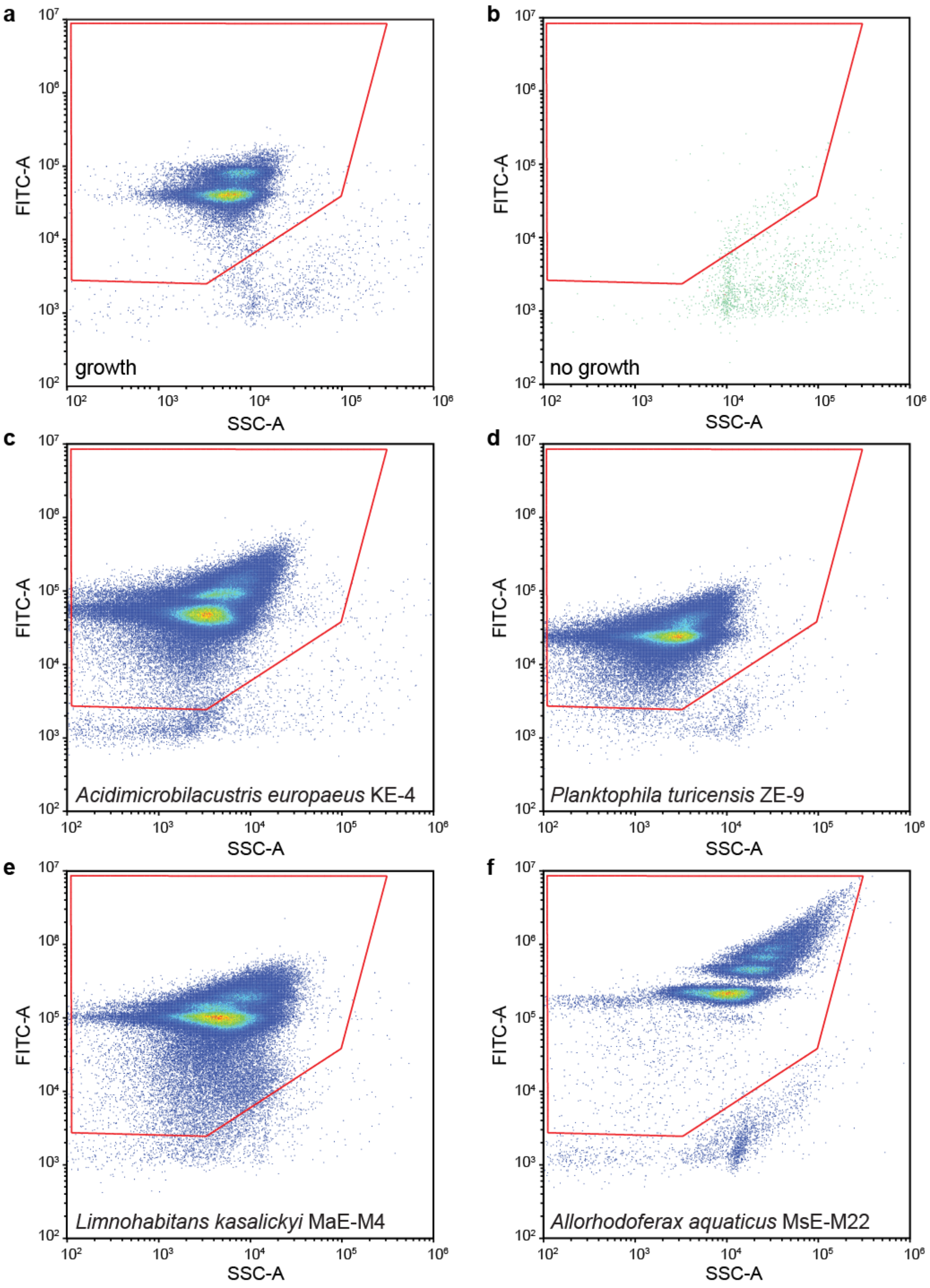

Supplementary Figure 21. Examples of flow cytometer cytograms. Cytograms show events recorded in 30 seconds, the red line indicates the gate used for quantification; x-axis: side scatter area (SSC-A), y-axis: fluorescence intensity of blue laser (488 nm; FITC-A). Examples include screening of 96-well plates (**a** growth, **b** no growth) and growth assays of different strains (**c** *A. europeaus* KE-4, **d** *P. turicensis* ZE-9, **e** *L. kasalickyi* MaE-M4, **f** *A. aquaticus* MsE-M22).

Supplementary References

1 Woodcroft, B. J. *et al.* Comprehensive taxonomic identification of microbial species in metagenomic data using SingleM and Sandpiper. *Nat. Biotechnol.* (2025). https://doi.org:10.1038/s41587-025-02738-1

2 Neuenschwander, S. M., Ghai, R., Pernthaler, J. & Salcher, M. M. Microdiversification in genome-streamlined ubiquitous freshwater Actinobacteria. *ISME J.* **12**, 185-198 (2018). https://doi.org:10.1038/ismej.2017.156

3 Linz, A. M. *et al.* Freshwater carbon and nutrient cycles revealed through reconstructed population genomes. *PeerJ* **6**, e6075 (2018). https://doi.org:10.7717/peerj.6075

4 Layoun, P. *et al.* Flexible genomic island conservation across freshwater and marine Methylophilaceae. *ISME J.* **18**, wrad036 (2024). https://doi.org:10.1093/ismejo/wrad036

5 Kavagutti, V. S. *et al.* High-resolution metagenomic reconstruction of the freshwater spring bloom. *Microbiome* **11**, 15 (2023). https://doi.org:10.1186/s40168-022-01451-4

6 Kavagutti, V. S., Andrei, A.-Ş., Mehrshad, M., Salcher, M. M. & Ghai, R. Phage-centric ecological interactions in aquatic ecosystems revealed through ultra-deep metagenomics. *Microbiome* **7**, 135 (2019). https://doi.org:10.1186/s40168-019-0752-0
