# Supplementary material for: Bringing the uncultivated microbial majority of freshwater ecosystems into culture
Source: Nat Commun. 2025 Aug 26;16:7971. doi: 10.1038/s41467-025-63266-9 (PMC12381374; doi:10.1038/s41467-025-63266-9)
Supplement: Supplementary file 2 — Description of Additional Supplementary Files [file 41467_2025_63266_MOESM2_ESM.pdf]

Supplementary Data for

Bringing the uncultivated microbial majority of freshwater ecosystems into culture

Michaela M. Salcher[1]*, Paul Layoun[1,2], Clafy Fernandes[1,2], Maria-Cecilia Chiriac[1], Paul-Adrian Bulzu[1], Rohit Ghai[1], Tanja Shabarova[1], Vojtech Lanta[3], Cristiana Callieri[4], Bettina Sonntag[5], Thomas Posch[6], Fabio Lepori[7], Petr Znachor[1], Markus Haber[1]

Corresponding author: michaelasalcher@gmail.com

Supplementary Data 1 to 21 are provided as a single Excel file with individual tabs.

Legends:

Supplementary Data 1: Media components.

Supplementary Data 2: Sampling details including metagenomic accession numbers and physico-chemical data collected during sampling.

Supplementary Data 3: Dilution-to-extinction cultivation details including taxonomy of 16S rRNA genes (SILVA) of cultures and GTDB assignment.

Supplementary Data 4: Results of statistical analyses used in this study. Significant *p*-values are highlighted in bold.

Supplementary Data 5: Raw data, maximum yield and growth rates from all growth assays with cultures.

Supplementary Data 6: Phylum-level classification of metagenomic reads with SingleM.

Supplementary Data 7: Genus-level classification of metagenomic reads with SingleM.

Supplementary Data 8: Phylum-level classification of 16S rRNA metagenomic reads with SILVA.

Supplementary Data 9: Genus/lineage-level classification of 16S rRNA metagenomic reads with SILVA.

Supplementary Data 10: Genus-level classification of 462 publicly available metagenomes with SingleM.

Supplementary Data 11: Details on genome-sequenced strains of the culture collection, type strains of novel species are highlighted in bold.

Supplementary Data 12: Details on metagenome-assembled genomes (MAGs) gained from the same water samples as cultures.

Supplementary Data 13: Details on publicly available reference genomes closely related to cultures.

Supplementary Data 14: Average nucleotide (ANI) matrices of cultures, MAGs and closely related reference genomes.

Supplementary Data 15: Average amino acid identity (AAI) matrix of cultures, MAGs and closely related reference genomes.

Supplementary Data 16: Metagenomic fragment recruitment of 67 samples taken from the same water samples as cultures.

Supplementary Data 17: Metagenomic fragment recruitment of 425 public datasets from six continents including time-series metagenomes from two lakes.

Supplementary Data 18: Selected metabolic modules (KEGG) present in the genomes.

Supplementary Data 19: Presence of CRISPR-Cas arrays in genomes from cultures, MAGs and closely related references.

Supplementary Data 20: Selected pathways related to methylotrophy present in the genomes.

Supplementary Data 21: Number and types of carbohydrate-active enzymes (CAZy) present in the genomes
