# Supplementary material for: Bringing the uncultivated microbial majority of freshwater ecosystems into culture
Source: Nat Commun. 2025 Aug 26;16:7971. doi: 10.1038/s41467-025-63266-9 (PMC12381374; doi:10.1038/s41467-025-63266-9)
Supplement: Supplementary file 4 — Reporting Summary [file 41467_2025_63266_MOESM4_ESM.pdf]

nature portfolio

Corresponding author(s): Michaela M. Salcher

Last updated by author(s): Aug 4, 2025

Reporting Summary

Nature Portfolio wishes to improve the reproducibility of the work that we publish. This form provides structure for consistency and transparency in reporting. For further information on Nature Portfolio policies, see our Editorial Policies and the Editorial Policy Checklist.

Statistics

For all statistical analyses, confirm that the following items are present in the figure legend, table legend, main text, or Methods section.

n/a	Confirmed	
☐	☒	The exact sample size (*n*) for each experimental group/condition, given as a discrete number and unit of measurement
☒	☐	A statement on whether measurements were taken from distinct samples or whether the same sample was measured repeatedly
☐	☒	The statistical test(s) used AND whether they are one- or two-sided<br>*Only common tests should be described solely by name; describe more complex techniques in the Methods section.*
☒	☐	A description of all covariates tested
☐	☒	A description of any assumptions or corrections, such as tests of normality and adjustment for multiple comparisons
☐	☒	A full description of the statistical parameters including central tendency (e.g. means) or other basic estimates (e.g. regression coefficient) AND variation (e.g. standard deviation) or associated estimates of uncertainty (e.g. confidence intervals)
☐	☒	For null hypothesis testing, the test statistic (e.g. *F*, *t*, *r*) with confidence intervals, effect sizes, degrees of freedom and *P* value noted<br>*Give P values as exact values whenever suitable.*
☒	☐	For Bayesian analysis, information on the choice of priors and Markov chain Monte Carlo settings
☒	☐	For hierarchical and complex designs, identification of the appropriate level for tests and full reporting of outcomes
☒	☐	Estimates of effect sizes (e.g. Cohen's *d*, Pearson's *r*), indicating how they were calculated

Our web collection on statistics for biologists contains articles on many of the points above.

Software and code

Policy information about availability of computer code

Data collection	Short-read paired end libraries (PE150) were sequenced on an Illumina Novaseq 6000 instrument. Long-read libraries were sequenced on a Nanopore PromethION platform.
Data analysis	Whole-genome sequencing: Raw reads obtained from Illumina Novaseq 6000 sequencing (PE150) were trimmed using BBMap v36.x (https://github.com/BioInfoTools/BBMap/) and assembled with SPAdes v3.12.0 (using kmers 29,39,49,59,69,79,89,99,109,119,129). The majority of assemblies resulted in <5 contigs that could manually be curated to a circular chromosome after repeated rounds of mapping of trimmed reads to contigs with Geneious 10 (default mapper, high sensitivity; www.geneious.com), extending contigs on both ends, identifying overlapping ends and assembling with the Geneious 10 assembler (de novo assembly, high sensitivity).<br>Metagenomic sequencing: Raw reads obtained from Illumina Novaseq 6000 sequencing (PE150) were quality controlled (Q>33), trimmed (trimq = 18 qtrim = rl), and adapters were removed using BBMap v36.x. We used SingleM (https://wwood.github.io/singlem/) for phylum- and genus-level classification (SingleM commands pipe and summarise) of quality filtered reads based on conserved regions of 59 single copy marker genes and the Genome Taxonomy Database (GTDB r214). In addition, SingleM taxonomic profiles from 462 public metagenomes were retrieved from Sandpiper 0.2.2 (https://sandpiper.qut.edu.au/) and further analyzed at the genus-level using SingleM summarise. To determine abundance of our isolate collection in these metagenomes,16S rRNA gene sequences of the isolate collection were assigned to the SILVA SSU database RefNR99 138.1 and taxonomically compared to GTDB r214 and r220 as follows: Matches to genome-sequenced representatives, matching genus names, MAGs containing 16S rRNA genes, and literature research. 16S rRNA gene classification of metagenomes was performed as follows: Metagenomes were subsampled to 20 million reads and 16S rRNA genes were predicted by using ublast and SSU-ALIGN with the RDP release 11 database clustered at 90% identity. The SILVA SSU database RefNR99 138.1 was used for taxonomic assignment. Hits were normalized by rRNA copy number following the rrnDB (https://rrndb.umms.med.umich.edu/) and manual curation (i.e., taxa with different naming in SILVA and rrnDB databases, uncultivated taxa for which we sequenced the first representatives). In case of unknown taxa in the rrnDB, the next higher taxonomic category was used for rRNA copy number estimation.

Metagenomic assembly and binning: De novo assembly of curated Illumina reads was done with MEGAHIT v1.1.4-2 using default parameters and the following k-mers: 29,49,69,89,109,119,129,149. Four samples were additionally sequenced using the Nanopore PromethION platform. For these, quality-controlled and trimmed Illumina short-read sequences were used for polishing noisy long-reads sequences by generating a Burrows Wheeler Transform (BWT), according to the ropebwt2 construction approach (https://github.com/lh3/ropebwt2). Nanopore basecalled long-reads with a Q score ≥ 8 were subjected to adapter and barcode trimming by Porechop (https://github.com/rrwick/Porechop) and further polished using the generated Illumina BWT with FMLRC2 v0.1.8 with default parameters. Polished long-reads were assembled using Flye v2.9.1-b1780 (settings: --nano-corr –meta –no-alt-contigs). For both Illumina and long-read assemblies, only contigs ≥3 kbp were further used for hybrid binning (tetranucleotide frequencies and coverage data) using MetaBAT2 with default parameters. Bins were manually curated and CheckM2 v1.0.1 was used to estimate bin completeness, contamination, and strain heterogeneity. Bins with ≥50% completeness and <5% contamination were selected for further analysis (4342 bins) and dereplicated using dRep (average nucleotide identity (ANI) >95%) resulting in 1294 representative MAGs (metagenome assembled genomes with highest dRep score). All MAGs and genomes from cultures were classified with the GTDB-Tk v2.4.0 toolkit based on the GTDB releases r214 and r220.

Phylogenomics and functional annotation of culture genomes, MAGs and publicly available reference genomes: Taxonomic classification was done with GTDB r220 as outlined above. All genomes from cultures were used to compute average nucleotide (ANI) and average amino acid identities (AAI) including the closest relatives to delineate species and genera at 95% ANI and 65% AAI, respectively. Prokka-1.11 was used for gene prediction and annotation was done with hmmsearch against InterProScan v5.46-81.0, COG, TIGRFAM v15.0, and KEGG databases. CRISPR-Cas systems were predicted with PILER-CR v1.06. Carbohydrate-active enzymes (CAZy) were predicted using hmmscan and the dbCAN CAZyme domain HMM database v9 (release date August 2022). Methylotrophic pathways were predicted with hmmscan and a published hmm database (Supplementary Dataset 2 in: https://academic.oup.com/ismej/article/13/11/2764/7475106).

A phylogenomic tree of all 87 genomes from cultures and 1294 de-replicated MAGs was calculated with GTDB-Tk using FastTree and pplacer. A detailed tree including all bacterial families with cultures was constructed by using 120 conserved proteins, of which at least 46 had to be present in the individual genomes (87 cultures, 313 MAGs and 324 closely related reference genomes). Five genomes of Patescibacteria were used as outgroup to root the tree. Protein sequences were aligned with PRANK, trimmed with BMGE (-m BLOSUM30 -t AA -g 0.5 -b 3), concatenated (https://github.com/nylander/catfasta2phyml), and a maximum likelihood tree was constructed with IQ-TREE 2 with ultrafast bootstrapping (1000 bootstrap replicates) and the best-fit model LG+F+I+G4, chosen by ModelFinder. Finally, a tree containing 87 culture genomes was constructed the same way (at least 85 markers present in individual genomes, model LG+F+I+G4).

Metagenomic fragment recruitment: All genomes from cultures (n = 87), dereplicated MAGs (n = 1,294) and closely related references (n = 324) were used for metagenomic fragment recruitment from 67 metagenomes that were collected from the same water samples used for cultivation campaigns. rRNA genes in genomes were masked prior to recruitment. MMseqs2 was used to map metagenomes (subsampled to 20 million reads) to each individual genome to obtain base coverage per gb (-minid 0.95 -mincov 0.9 -minlen 50). Additionally, we used 250 publicly available metagenomes from lakes from six continents and two seasonally resolved metagenomic multiannual time series from Lake Mendota, USA (2008-2012, n=94), and the Římov Reservoir, Czechia (2015-2019, n=81)for fragment recruitment using 47 culture genomes dereplicated at the species level (95% ANI).

Software used in this study:
BBMap v36.x
reformat.sh
bbduk.sh
bbmerge.sh
SPAdes v3.12.0
Geneious 10
SingleM
SSU-ALIGN
ublast
MEGAHIT v1.1.4-2
ropebwt2
porechop
FMLRC2 v0.1.8
Flye v2.9.1-b1780
bbwrap.sh
jgi_summarize_bam_contig_depths
MetaBAT2
CheckM2 v1.0.1
GTDB-Tk v2.4.0
dRep
HMMsearch
InterProScan v5.46-81.0
Prokka 1.11
MMseqs2
FastTree
pplacer
PRANK
BMGE v1.12
IQ-TREE v2.1.2
ModelFinder
R package cluster v2.0.7-1

For manuscripts utilizing custom algorithms or software that are central to the research but not yet described in published literature, software must be made available to editors and reviewers. We strongly encourage code deposition in a community repository (e.g. GitHub). See the Nature Portfolio guidelines for submitting code & software for further information.

Data

Policy information about availability of data

All manuscripts must include a data availability statement. This statement should provide the following information, where applicable:

- Accession codes, unique identifiers, or web links for publicly available datasets
- A description of any restrictions on data availability
- For clinical datasets or third party data, please ensure that the statement adheres to our policy

The sequence data generated in this study have been deposited in ENA under BioProject accession numbers PRJEB77526 (culture genomes) and PRJEB35640 (MAGs) and raw metagenomic reads under BioProject number PRJEB35640. Accession numbers of metagenomes are listed in Supplementary Data 2, accession numbers of culture genomes in Supplementary Data 11, and accession numbers of MAGs in Supplementary Data 12. Accession numbers of publicly available metagenomic datasets and genomes can be found in Supplementary Data 17 and 13, respectively. Novel families, genera, and species names were registered at SeqCode in register lists seqco.de/r:o5wsiwof and seqco.de/r:opjv7zsc. Phylogenomic trees are available at iTOL (https://itol.embl.de/shared/eik87zth8PG1).

Public databases used in this study:
TIGRFAMs v15.0
Pfam release 32
NCBI NR
blastn
dbCAN CAZyme domain HHM database v9
COGs
rrnDB
RDP release 11
KOfam
KEGG
SILVA SSU RefNR99 138.1
GTDB r214 and r220
Sandpiper 0.2.2

Research involving human participants, their data, or biological material

Policy information about studies with human participants or human data. See also policy information about sex, gender (identity/presentation), and sexual orientation and race, ethnicity and racism.

Reporting on sex and gender	This study does not involve human research participants.
Reporting on race, ethnicity, or other socially relevant groupings	This study does not involve human research participants.
Population characteristics	This study does not involve human research participants.
Recruitment	This study does not involve human research participants.
Ethics oversight	This study does not involve human research participants.

Note that full information on the approval of the study protocol must also be provided in the manuscript.

Field-specific reporting

Please select the one below that is the best fit for your research. If you are not sure, read the appropriate sections before making your selection.

☐ Life sciences ☐ Behavioural & social sciences ☒ Ecological, evolutionary & environmental sciences

For a reference copy of the document with all sections, see nature.com/documents/nr-reporting-summary-flat.pdf

Ecological, evolutionary & environmental sciences study design

All studies must disclose on these points even when the disclosure is negative.

Study description	The study reports the isolation and characterization of bacterial strains isolated from 14 lakes in Central Europe. Cultures were obtained by high-throughput dilution-to-extinction cultivation using artificial media that reflect natural conditions in lakes. We obtained 627 axenic strains with stable growth >1 year. Several selected strains were characterized in short-term growth experiments. 87 strains were genome-sequenced and compared to metagenome-assembled genomes (MAGs) sequenced from the same water samples. The nature of the study does not necessitate any treatment factors, interactions, design structure (factorial, nested, hierarchical) or replicates.
Research sample	Water samples were taken from 14 lakes in Central Europe covering the full spectrum of trophic states from ultra-oligotrophic

(nutrient-poor) to eutrophic (nutrient-rich). Lakes were sampled at the deepest point and water was taken from two depths that represent the epi- and hypolimnion of the respective lake. Sampling was carried out in spring and autumn 2019, and six lakes were additionally sampled in summer 2019. This sampling strategy ensured a wide range of different freshwater samples regarding nutrient content, water depth and seasons. The recovered isolates represent some of the most abundant species in freshwater systems, the recovered metagenome-assembled genomes (MAGs) reflect the total diversity of the sampled lakes. We further included publicly available freshwater metagenomes (n=462) from seven continents to assess the global distribution of our strain collection.

Total number of samples: 67

Total number of isolated bacterial strains: 627

Total number of MAGs: 1294 (dereplicated at species level)

Taxonomy of isolated bacterial strains: at least 72 genera (based on taxonomy assignment of 16S rRNA genes) from 8 phyla

Taxonomy of genome-sequenced bacterial strains: 47 species (41 newly described here) from 5 phyla

Taxonomy of MAGs: 1294 species from 31 phyla

Sampling strategy

Water samples were taken from 14 lakes in Central Europe covering the full spectrum of trophic states from ultra-oligotrophic (nutrient-poor) to eutrophic (nutrient-rich). Lakes were sampled at the deepest point and 10-50 l of water was taken from two depths that represent the epi- and hypolimnion of the respective lake. Sampling was carried out in spring and autumn 2019, and six lakes were additionally sampled in summer 2019. This sampling strategy ensured a wide range of different freshwater samples regarding nutrient content, water depth and seasons. Water samples were prefiltered with a 20 µm net and sequentially passed through filters with gradually smaller pore sizes (5 µm, 0.22 µm) until 0.22 µm filters were clogged (3-34 l per sample, depending on lake, depth, and sampling date). Duplicates or triplicates of 0.22 µm filters were collected. Biomass retained on the 0.22 µm filter was used for DNA extraction and metagenomic sequencing. A sampling volume of 3-34 l contains approx. 10^9-10^11 prokaryotic cells and is thus representative for the total bacterioplankton for metagenomic sequencing. For the isolation of microbes, 10 ml samples were filtered through 0.4 µm filters to remove larger organisms and used for dilution-to-extinction isolation with 1 cell per well in 96-deep-well plates. We did not perform any sample size calculations for this study.

Data collection

Water samples were taken from 14 lakes in Central Europe. Lakes were sampled at the deepest point and water was taken from two depths that represent the epi- and hypolimnion of the respective lake. Sampling was carried out in spring and autumn 2019, and six lakes were additionally sampled in summer 2019. Sampling was conducted by Michaela M. Salcher, Paul Layoun, Rohit Ghai, Tanja Shabarova, Vojtech Lanta, Cristiana Callieri, Bettina Sonntag, Thomas Posch, Fabio Lepori, Petr Znachor, Markus Haber, Eugen Loher, Andreja Kust, Sebastian Mayer, Patrik Pejsar and Pavel Rychtecky.

Vertical profiles of water temperature, conductivity, pH, oxygen, and chlorophyll a concentrations were recorded with a submersible probe (YSI EXO2, Yellow Springs Instruments, Yellow Springs, USA).

Two different water layers each were sampled with a Niskin bottle or Friedinger sampler for the isolation of microbes and metagenomics: the upper water layer (epilimnion, 5 m depth) and the oxygenated deep-water layer (hypolimnion, variable depth depending on the maximal depth). A total of 3-34 l of each water sample was prefiltered via 20 µm plankton nets and serially filtered through 5 µm (Sterlitech PES membrane filters, USA) and 0.22 µm (Millipore express PLUS, Germany) polysulfone filters with a peristaltic pump until filters were clogged and filters were stored at -80°C until DNA isolation. Two hundred ml of the 0.22 µm filtrate were collected for nutrient analyses (phosphorus, nitrate and dissolved organic carbon).

DNA from 0.22 µm lake water metagenome filters was extracted using the ZR Soil Microbe DNA MiniPrepTM kit (Zymo Research, Irvine, CA, USA) according to the manufacturer's instructions. Short-read paired-end DNA sequencing data were generated using the Illumina NovaSeq 6000 platform (Novogene, Hong Kong, China). Long-read Oxford Nanopore data were generated using a PromethION platform.

Timing and spatial scale

Sample name: sampling date, water depth, Location of individual samplings:

AE-03may19: 03.05.2019, 5 m depth, Attersee

AE-24oct19: 24.10.2019, 5 m depth, Attersee

AH-03may19: 03.05.2019, 150 m depth, Attersee

AH-24oct19: 24.10.2019, 150 m depth, Attersee

GE-03apr19: 03.04.2019, 5 m depth, Greifensee

GE-07nov19: 07.11.2019, 5 m depth, Greifensee

GH-07nov19: 07.11.2019, 20 m depth, Greifensee

LE-02apr19: 02.04.2019, 5 m depth, Lake Lugano

LE-05nov19: 05.11.2019, 5 m depth, Lake Lugano

LH-02apr19: 02.04.2019, 50 m depth, Lake Lugano

LH-05nov19: 05.11.2019, 50 m depth, Lake Lugano

MaE-09apr19: 09.04.2019, 5 m depth, Lake Maggiore

MaE-04nov19: 04.11.2019, 5 m depth, Lake Maggiore

MaH-09apr19: 09.04.2019, 300 m depth, Lake Maggiore

MaH-04nov19: 04.11.2019, 300 m depth, Lake Maggiore

ME-08apr19: 08.04.2019, 5 m depth, Lake Medard

ME-09jul19: 09.07.2019, 5 m depth, Lake Medard

ME-21oct19: 21.10.2019, 5 m depth, Lake Medard

ME-04may21: 04.05.2021, 5 m depth, Lake Medard

MH-08apr19: 08.04.2019, 30 m depth, Lake Medard

MH-09jul19: 09.07.2019, 30 m depth, Lake Medard

MH-21oct19: 21.10.2019, 30 m depth, Lake Medard

MiE-16apr19: 16.04.2019, 5 m depth, Lake Milada

MiE-22jul19: 22.07.2019, 5 m depth, Lake Milada

MiE-14oct19: 14.10.2019, 5 m depth, Lake Milada

MiH-16apr19: 16.04.2019, 15 m depth, Lake Milada

MiH-22jul19: 22.07.2019, 15 m depth, Lake Milada

MiH-14oct19: 14.10.2019, 15 m depth, Lake Milada
MoE-02may19: 02.05.2019, 5 m depth, Mondsee
MoE-23oct19: 23.10.2019, 5 m depth, Mondsee
MoH-02may19: 02.05.2019, 40 m depth, Mondsee
MoH-23oct19: 23.10.2019, 40 m depth, Mondsee
MsE-30apr19: 30.04.2019, 5 m depth, Lake Most
MsE-30jul19: 30.07.2019, 5 m depth, Lake Most
MsE-01oct19: 01.10.2019, 5 m depth, Lake Most
MsH-30apr19: 30.04.2019, 50 m depth, Lake Most
MsH-30jul19: 30.07.2019, 50 m depth, Lake Most
MsH-01oct19: 01.10.2019, 50 m depth, Lake Most
TE-08apr19: 08.04.2019, 5 m depth, Lake Thun
TE-11nov19: 11.11.2019, 5 m depth, Lake Thun
TH-08apr19: 08.04.2019, 180 m depth, Lake Thun
TH-11nov19: 11.11.2019, 180 m depth, Lake Thun
TrE-03may19: 03.05.2019, 5 m depth, Traunsee
TrE-25oct19: 25.10.2019, 5 m depth, Traunsee
TrH-03may19: 03.05.2019, 150 m depth, Traunsee
TrH-25oct19: 25.10.2019, 150 m depth, Traunsee
ZLE-24apr19: 24.04.2019, 5 m depth, Zlutice Reservoir
ZLE-06aug19: 06.08.2019, 5 m depth, Zlutice Reservoir
ZLE-19nov19: 19.11.2019, 5 m depth, Zlutice Reservoir
ZLH-24apr19: 24.04.2019, 15 m depth, Zlutice Reservoir
ZLH-06aug19: 06.08.2019, 15 m depth, Zlutice Reservoir
ZLH-19nov19: 19.11.2019, 5 m depth, Zlutice Reservoir
KE-25apr19: 25.04.2019, 5 m depth, Klicava Reservoir
KE-07aug19: 07.08.2019, 5 m depth, Klicava Reservoir
KE-20nov19: 20.11.2019, 5 m depth, Klicava Reservoir
KH-25apr19: 25.04.2019, 15 m depth, Klicava Reservoir
KH-07aug19: 07.08.2019, 15 m depth, Klicava Reservoir
KH-20nov19: 20.11.2019, 15 m depth, Klicava Reservoir
ZE-03apr19: 03.04.2019, 5 m depth, Lake Zurich
ZE-13nov19: 13.11.2019, 5 m depth, Lake Zurich
ZH-03apr19: 03.04.2019, 80 m depth, Lake Zurich
ZH-13nov19: 13.11.2019, 80 m depth, Lake Zurich
RE-23apr19: 23.04.2019, 0.5 m depth, Rimov Reservoir
RE-26aug19: 26.08.2019, 0.5 m depth, Rimov Reservoir
RE-18nov19: 18.11.2019, 0.5 m depth, Rimov Reservoir
RH-23apr19: 23.04.2019, 30 m depth, Rimov Reservoir
RH-26aug19: 26.08.2019, 30 m depth, Rimov Reservoir
RH-18nov19: 18.11.2019, 30 m depth, Rimov Reservoir

Data exclusions

No data was excluded from the analyses.

Reproducibility

The data generated are available in referenced public repositories, ensuring transparency and accessibility. All methods used in this study are cited, and the parameters for software applications are fully documented. Given the study's reliance on environmental samples, no attempts were made to replicate the metagenomic samples. Bacterial cultures were maintained for >1 year in 10-week cycles consisting of 1:10 transfers (0.5 ml culture to 4.5 ml medium), six week incubation, 1:1 fills (adding 5 ml fresh medium), followed by another 4 week incubation after which cultures were measured on the flow cytometer and again 1:10 transferred to start the next cycle. Glycerol stocks were prepared for all strains. Short-term growth assays were performed for 18 strains by growing them in six different media, cell densities were quantified by flow cytometry 2-3x per week for a period of 17-32 days. Growth assays were performed in triplicates with low variations between replicates.

Randomization

Randomization is not relevant to the study design as this is an exploratory study.

Blinding

Blinding is not relevant to the study design as this is an exploratory study without a priori expectations that would influence the analyses.

Did the study involve field work? ☒ Yes ☐ No

Field work, collection and transport

Field conditions

All details on field conditions for each sampling (Water temperature, conductivity, oxygen content, pH, chlorophyll a content, dissolved organic carbon, dissolved phosphorus, alkalinity and concentrations of ions) can be found in Supplementary Data 2. Weather conditions at the time of sampling were typical for each location, and no sampling occurred during weather anomalies.

Location

Attersee (469 m a.s.l., 47°52′ N 13°32′ E, Austria, 171 m max. depth)
Greifensee (435 m a.s.l., 47°22′ N 8°42′ E, Switzerland, 32 m max. depth)

	Lake Lugano (271 m a.s.l., 45°59′ N 8°58′ E, Italy, 288 m max. depth)<br>Lake Maggiore (193 m a.s.l., 45°58′ N 8°39′ E, Italy, 370 m max. depth)<br>Lake Medard (400 m a.s.l., 50°10′ N 12°35′ E, Czechia, 50 m max. depth)<br>Lake Milada (137 m a.s.l., 50°38′ N 13°56′ E, Czechia, 20 m max. depth)<br>Mondsee (483 m a.s.l., 47°49′ N 13°22′ E, Austria, 68 m max. depth)<br>Lake Most (199 m a.s.l., 50°31′ N 13°37′ E, Czechia, 75 m max. depth)<br>Lake Thun (558 m a.s.l., 46°41′ N 7°43′ E, Switzerland, 217 m max. depth)<br>Traunsee (423 m a.s.l., 47°52′ N 13°48′ E, Austria, 191 m max. depth)<br>Zlutice Reservoir (497 m a.s.l., 50°05′ N 13°07′ E, Czechia, 17 m max. depth)<br>Klicava Reservoir (299.2 m a.s.l., 50°03′ N 13°55′ E, Czechia, 34 m max. depth)<br>Lake Zurich (406 m a.s.l., 47°18′ N 8°34′ E, Switzerland, 136 m max. depth)<br>Rimov Reservoir (470 m a.s.l., 48°50′ N 14°29′ E, Czechia, 43 m max. depth)<br><br>More details on the individual lakes (trophic status, area, mixis type, water retention time, biogeographic region) can be found in Table S1 in the Supplementary Dataset.
Access & import/export	All samples were collected in compliance with local, cantonal and national laws. No permits were required for sampling of most lakes. Sampling at Lake Medard, Lake Milada, Lake Most, Rimov Reservoir, Klicava Reservoir and Zlutice Reservoir was carried out based on an agreement between the Biology Centre CAS, the Institute of Hydrobiology and the Povodí Vltavy, State Enterprise, on the basis of the legal permit issued by the Povodí Vltavy, State Enterprise for the solution of the project "Biomanipulation as a tool for improving water quality in reservoirs", No. CZ.02.1.01/0.0/0.0/16_025/0007417. The majority of coauthors are affiliated with the Biology Centre CAS.<br>Sampling at Attersee, Mondsee, and Traunsee was conducted in collaboration with the Research Institute of Limnology (University of Innsbruck, Austria) in Mondsee. Bettina Sonntag is a coauthor of the study.<br>Sampling at Greifensee and Lake Zurich was conducted in collaboration with the Limnological Station (University of Zurich, Switzerland) in Kilchberg. Thomas Posch is a coauthor of the study.<br>Sampling of Lake Thun occurred in partnership with the Canton of Bern's Laboratory for Water and Soil Protection (Switzerland).<br>Sampling of Lake Maggiore was done in collaboration with the Water Research Institute (IRSA) of the National Research Council (CNR) in Verbania, Italy. Cristiana Callieri is a coauthor of this study.<br>Sampling of Lake Lugano was done in collaboration with the University of Applied Sciences and Arts of Southern Switzerland (SUPSI) in Mendrisio, Switzerland. Fabio Leppori is a coauthor of this study.
Disturbance	No disturbances were caused by the sampling procedures.

Reporting for specific materials, systems and methods

We require information from authors about some types of materials, experimental systems and methods used in many studies. Here, indicate whether each material, system or method listed is relevant to your study. If you are not sure if a list item applies to your research, read the appropriate section before selecting a response.

Materials & experimental systems

n/a	Involved in the study
☒	☐ Antibodies
☒	☐ Eukaryotic cell lines
☒	☐ Palaeontology and archaeology
☒	☐ Animals and other organisms
☒	☐ Clinical data
☒	☐ Dual use research of concern
☒	☐ Plants

Methods

n/a	Involved in the study
☒	☐ ChIP-seq
☐	☒ Flow cytometry
☒	☐ MRI-based neuroimaging

Plants

Seed stocks	NA
Novel plant genotypes	NA
Authentication	NA

Flow Cytometry

Plots

Confirm that:

☒ The axis labels state the marker and fluorochrome used (e.g. CD4-FITC).

☒ The axis scales are clearly visible. Include numbers along axes only for bottom left plot of group (a 'group' is an analysis of identical markers).

☒ All plots are contour plots with outliers or pseudocolor plots.

☒ A numerical value for number of cells or percentage (with statistics) is provided.

Methodology

Sample preparation	Water samples and cultures were stained with SYBRgreen I (0.5 x final concentration; Lonza, Rockland, ME, USA).
Instrument	CytoFLEX S flow cytometer (Beckman Coulter; Brea, CA, USA) equipped with a blue laser (488nm, bandpass filters 525/40 and 690/50)
Software	CytoExpert version 2.4.0.28 (Beckman Coulter; Brea, CA, USA)
Cell population abundance	We used flow cytometry for quantification, we did not sort specific populations. Cytograms (pseudocolor plots) with FITC-A as y-axis and SSC-A as x-axis were used for analysis and populations within gates (see below) were used for the calculation of cell abundances.
Gating strategy	Polygon gates were drawn for bacteria starting from approx. 2 x 10^3 to 8 x 10^6 on the y-axis (FITC-A) and 10^2 to 3 x 10^5 with slopes between approx. 3 x 10^3 to 10^5 on the x-axis (SSC-A). The gates were set with positive controls (bacterial cultures) and negative controls (media only). Identical gates were used for all experiments. Examples of cytograms are provided as Supplementary Fig. 21.

☒ Tick this box to confirm that a figure exemplifying the gating strategy is provided in the Supplementary Information.
